# Integrative miRNA-mRNA profiling uncovers mechanisms of belimumab action in systemic lupus erythematosus

**DOI:** 10.3389/fimmu.2025.1553971

**Published:** 2025-03-14

**Authors:** Maria Royo, Blanca Joseph-Mullol, Sebastian Sandoval, Teresa Moliné, Cristina Solé, Josefina Cortés-Hernández

**Affiliations:** ^1^ Rheumatology Research Group, Lupus Unit, Hospital Universitari Vall d’Hebron, Institut de Recerca (VHIR), Universitat Autònoma de Barcelona, Barcelona, Spain; ^2^ Department of Pathology, Hospital Universitari Vall d’Hebron, Institut de Recerca (VHIR), Universitat Autònoma de Barcelona, Barcelona, Spain

**Keywords:** systemic lupus erythematosus, miRNA, mRNA, integrative “omics”, belimumab, mechanism of action, immune cells

## Abstract

Systemic lupus erythematosus (SLE) is a complex autoimmune disorder driven by autoreactive B cells and characterized by the production of pathogenic autoantibodies. Belimumab, an anti-BAFF monoclonal antibody, has demonstrated efficacy in reducing disease activity and corticosteroid use in SLE patients, although responses remain variable. B-cell activating factor (BAFF) is essential for B cell survival and autoantibody production, positioning it as a key target in SLE pathogenesis. MicroRNAs (miRNAs), critical regulators of gene expression and immune homeostasis, have an emerging role in SLE pathophysiology. However, their regulation in response to anti-BAFF therapies, such as belimumab, remains unexplored. This study investigates miRNA-mRNA interactions in T cells, B cells, and myeloid cells from SLE patients before and after belimumab treatment. A total of 79 miRNAs associated with treatment response and 525 miRNA-gene interactions were identified. Validation in 18 SLE responders revealed significant changes in miRNA expression in T and myeloid cells, but not in B cells. Belimumab was found to modulate B cell development by regulating genes such as BLNK, BANK1, and MEF2C, as well as the CD40/CD40L axis. In T cells, miRNAs influenced interferon signaling and inflammatory cytokines via NF-κB activation. Changes in myeloid cells, characterized by the downregulation of KLF13, CCL5, and IL4, appear to be secondary to T cell modulation. These findings provide novel insights into the miRNA-mediated regulatory networks underlying belimumab’s immunomodulatory effects in SLE. Further research is required to validate these findings and through *in vitro* experiments to better understand the role of miRNAs in guiding treatment responses.

## 1 Introduction

Systemic lupus erythematosus (SLE) is a multifaceted and heterogeneous autoimmune disorder characterized by the production of autoantibodies derived from autoreactive B cells, which are pivotal to its pathogenesis (1).

B-cell activating factor (BAFF), also referred to as B Lymphocyte Stimulator (BLyS), is a cytokine belonging to the tumor necrosis factor (TNF) family that plays a crucial role in B cell survival, maturation, and function. BAFF contributes to the pathogenesis of autoimmune diseases by promoting B cell proliferation and survival, regulating class-switch recombination, and enhancing plasma cell expansion. These processes favor the selection and persistence of autoreactive B cells, ultimately leading to increased autoantibody production and immune complex deposition. Additionally, BAFF enhances the antigen-presenting activity of B cells, stimulates the production of Th1 cytokines, and upregulates costimulatory molecules such as CD40, ICOSL, and MHC class II. These effects collectively drive Th1 activation and expansion, as well as increased chemotaxis of proinflammatory cells via the CCR6-CCL20 axis. Beyond its impact on B cells, BAFF also influences other immune cell populations. Through BAFF-R expressed on T cells and innate immune cells, BAFF supports T cell activation, proliferation, and differentiation, while promoting monocyte survival and differentiation into activated macrophages, further amplifying inflammatory responses (2).

Compelling evidence underscores the pivotal role of BAFF in lupus pathogenesis. Elevated BAFF levels in serum and tissue correlate with autoantibody production and disease activity (3, 4). In murine models, BAFF overexpression induces an SLE-like phenotype, while its inhibition delays disease onset, highlighting its role in lupus progression (5, 6). Belimumab, an anti-BAFF/BlyS monoclonal antibody, has demonstrated efficacy in clinical trials, reducing disease activity, flare rates, improving patient quality of life, and decreasing corticosteroid dependence, leading to its approval as the first targeted biologic therapy for SLE (7). However, its moderate effectiveness and patient response variability highlight the need for a deeper understanding of the molecular pathways involved (8, 9). Recent studies have identified a set of genes, including CCL4L2, CARD10, MMP15, and KLRC2, as potential predictors of response to belimumab, demonstrating an 84% specificity in cross-validation. In contrast, non-response has been associated with disruptions in critical pathways, such as cell cycle checkpoints, the PI3K/Akt/mTOR pathway, and TGF-beta signaling.

MicroRNAs (miRNAs) are endogenous, small (~22 nt) noncoding RNAs that regulate mRNA stability and translation. They influence a wide range of physiological and pathological processes including differentiation, proliferation and apoptosis. Recent studies have linked dysregulated miRNA expression to several human diseases, including autoimmune disorders such as SLE, with the potential to serve as valuable diagnostic and therapeutic biomarkers (10). In SLE, altered miRNA expression in T cells and B cells contributes to immune hyperactivation (11). Specifically, downregulation of miR-145, miR-142, and miR-125a impairs T-cell regulation, while upregulation of miR-7, miR-21, and miR-22 in B-cells enhances their activation by inhibiting PTEN, thereby promoting autoantibody production (12). Additionally, IL-21 further amplifies this effect by increasing the expression of miR-7 and miR-22, strengthening T-B cell interactions (12). In plasmacytoid dendritic cells (pDCs), reduced levels of miR-361-5p, miR-128-3p, and miR-181-2-3p correlate with elevated type 1 interferon production, a key driver of lupus pathogenesis (13). Although miRNAs are recognized for their role in immune regulation and their potential as biomarkers in SLE, their role in predicting responses to anti-BLyS therapy has not yet been explored.

To explore the mechanisms underlying anti-BLyS therapy in SLE, this study investigated miRNA-mRNA interaction networks and biological pathways in immune cellular subsets associated with clinical improvement. Differentially expressed miRNAs and mRNAs were identified in CD3^+^CD19^-^ T cells, CD3^+^CD19^+^ B cells, and CD11c^+^ cells from SLE patients pre- and post-treatment, enabling the generation of a comprehensive transcriptomic profile. This integrative analysis reveals novel mRNA and miRNA expression patterns, as well as regulatory networks, offering new insights into the molecular response to BLyS-targeted therapy.

## 2 Materials and methods

### 2.1 Study design and subjects

Patients requiring belimumab treatment for active SLE as part of standard care at the Lupus Unit of Vall Hebron University Hospital in Barcelona, between January 2021 and December 2022, were recruited for this study. Eligible participants were adults meeting the ACR/EULAR 2019 SLE criteria (14), on stable doses of antimalarials and/or immunosuppressives for at least 12 weeks and corticosteroids for 2 weeks before inclusion. All patients had completed 6 months of belimumab therapy with a clinical response, defined as achieving LLDAS (15) by week 24, which was further sustained for up to 1 year. Exclusion criteria included concomitant rheumatic or oncologic diseases, pregnancy, prior B-cell therapy within 12 months, active infection, renal or neurological involvement, failure to achieve LLDAS, or unavailable PBMC samples. The study adhered to the Declaration of Helsinki and Good Clinical Practice principles, and it was approved by the Vall d’Hebron Ethics Committee (PR(AG)441/2024). All patients provided written informed consent. Patients received weekly subcutaneous belimumab (200 mg) alongside conventional therapy. During follow-up, patients underwent regular clinical and laboratory assessments every three months for up to one year. Disease activity was assessed using the SLE Disease Activity Index (SLEDAI-2K) (14) and the Physician’s Global Assessment (PGA) scoring (0-3 scale). Clinical response was defined based on the Lupus Low Disease Activity State (LLDAS) (15). These criteria include: SLEDAI-2K ≤4, with no new activity in major organ systems and no hemolytic anemia or gastrointestinal activity; no new features of disease activity compared with the previous assessment; SELENA-SLEDAI PGA ≤1; current prednisolone (or equivalent) dose ≤7.5 mg/day and well-tolerated standard maintenance doses of immunosuppressive drugs and approved biologic agents, excluding investigational drugs.

Although SLE is highly heterogeneous, we minimized variability by selecting a clinically homogeneous population. The screening cohort included 10 patients, while the validation cohort consisted of an additional 18 patients, with samples collected before and after belimumab treatment. Additionally, we included a cohort of SLE patients who were not treated with belimumab, all of whom were treated homogeneously with mycophenolate (n=5). These participants were selected based on the same inclusion/exclusion criteria.

### 2.2 Biological samples

Peripheral blood mononuclear cells (PBMCs) and serum were collected pre- and post-belimumab treatment (6 months). PBMCs were isolated via Ficoll-Hypaque gradient centrifugation (Vacutainer CPT, BD Biosciences), cryopreserved in heat-inactivated fetal bovine serum (FBS, Gibco) with 10% DMSO (ThermoFisher Scientific, Waltham, MA, USA) and stored in liquid nitrogen for a maximum period of two months before processing. Serum samples were stored at −20°C.

### 2.3 Flow cytometry analysis

PBMC immune subsets were analyzed using a 17-color flow cytometry panel. Staining was performed with specific antibodies (**Supplementary Table S1**), followed by incubation with Brilliant Stain Buffer, washing, and gating strategies to exclude non-viable cells and doublets. Cells were sorted into three immune subsets: CD3^+^CD19^-^ (T cells), CD3^-^CD19^+^ (B cells), and CD11c^+^CD3^-^CD19^-^ (myeloid cells) using a Cytek Aurora Spectrum Cytometer. After sorting, RNA and miRNA extraction was performed immediately. Quantification of different immune cell types was performed using FlowJo software (version 10.10) with further details provided in the **Supplementary Material**. The gating strategy is illustrated in **Supplementary Figure S1**.

### 2.4 RNA and miRNA extraction

RNA and miRNA were extracted using the RNeasy Mini Kit, following the manufacturer’s instructions. Quality and concentration were assessed using the Bioanalyzer PicoChip (details in **Supplementary Material** and RIN values in **Supplementary Table S2**). Extraction was performed on the same day as cell sorting, and samples were stored at -80°C until microarray processing to ensure optimal preservation, consistent distribution and to minimize batch effects (details in **Supplementary Material**).

### 2.5 mRNA and miRNA microarray

Gene and miRNA expression profiling used Clariom S assay and GeneChip miRNA 4.0 arrays, respectively (Thermo Fisher Scientific, Inc.). After hybridization and washing, the arrays were scanned by an Affymetrix Microarray Scanner (Applied Biosystems, Grand Island, NY, USA). Raw data of HTA 2.0 were extracted and normalized by Affymetrix^®^ Transcriptome Analysis Console (TAC) Software (Thermo Fisher Scientific, Inc.). miRNA QC Tool software (Thermo Fisher Scientific, Inc.) was used for miRNA 4.0 array data summarization, normalization, and quality control. The array data has been deposited in the GEO database at NCBI under the accession number GSE283865.

### 2.6 Microarray analysis

Differential expressions were assessed using linear models with empirical Bayes moderation. Quality control procedures included principal component analysis (PCA), heatmaps, and clustering. Enrichment analysis of biological processes and pathways was performed using the Gene Ontology (GO) and Reactome databases, focusing on adjusted p-values <0.05 (details in **Supplementary Material**).

### 2.7 Validation of differential mRNA and miRNA expression by qRT-PCR

The most significantly differentially expressed mRNAs and miRNAs were validated using qRT-PCR. cDNA templates were synthesized using either the High-Capacity RNA-to-cDNA Kit or the TaqMan Advanced miRNA cDNA Synthesis Kit (Applied Biosystems, Foster City, CA), according to the manufacturer’s instructions. mRNA and miRNA expression levels were quantified via RT-qPCR on the QuantStudio 5 Pro PCR instrument (Applied Biosystems, Foster City, CA) using the respective TaqMan assays or TaqMan Advanced miRNA assays, along with TaqMan Fast Master Mix (Applied Biosystems, Foster City, CA) (**Supplementary Table S3**). Data normalization was performed using GAPDH for mRNA or miR-8069 for miRNA, both identified through microarray data as the most suitable endogenous controls for gene and miRNA expression analysis.

### 2.8 Target gene prediction for miRNAs

The multiMiR Bioconductor package (16–18) was used to identify the target genes of the selected miRNAs subsets. This package enables the retrieval of miRNA-target interactions from various databases, including both predicted targets (DIANA microT, ElMMo, MicroCosm, miRanda, miRDB, PicTar, PITA, and TargetScan) and validated targets (miRecords, miRTarBase, and TarBase). Target prediction was performed for a subset of miRNAs in each comparison, selected based on the following statistical criteria: miRNAs with a raw p-value less than 0.05 and an absolute log fold change (logFC) greater than 0.5.

### 2.9 miRNA-mRNA interaction analysis

Integrative analysis of mRNA and miRNA data was performed using the DIABLO method from the mixOmics R package. This method extends Generalised Canonical Correlation Analysis (19), which generalizes Partial Least Squares (PLS) for multiple matching datasets. In its sparse version (sPLS-DA), variable selection is applied to identify the most predictive or discriminative features, aiding in the classification of the samples (20).

### 2.10 miRNA-mRNA network and pathway enrichment analysis

ClusterProfiler (21) was used to analyze genes from one 114 miRNA-mRNA interactions to identify enriched biological terms from the Reactome Pathway Database (22) and GO. Networks connecting miRNA-mRNA interactions and enriched biological terms were created using Cytoscape (23). For the network of enriched biological terms, only Reactome pathways related to the ‘Immune System’ category and Gene Ontology terms related to the ‘Immune System Process’ were considered.

### 2.11 Statistical analysis

Statistical analysis was performed using GraphPad Prism software version 6.0.1. The results are presented as means ± SEM and were analyzed using one-way ANOVA followed by Tukey’s test to detect differences between groups. Categorical variables were expressed as counts and percentages of patients (%) and were compared using Fisher’s exact test. Sample size estimation was performed using the pwr package in R, assuming a large effect size (d = 1.2), 80% statistical power, and a significance level of 0.05. A p value < 0.05 was considered statistically significant for each of the experiments.

## 3 Results

### 3.1 Peripheral blood leukocyte subset distribution in SLE following belimumab treatment

Ten consecutive SLE patients treated with belimumab, classified as LLDA responders at 6 months with available PBMC samples, were included in the study. Demographic and baseline clinical characteristics are shown in **Table 1**. After 6 months of therapy, there was a significant improvement in disease activity, with the mean SLEDAI-2K score decreasing to 2.4 ± 1.1 and a PGA score to 0.3 ± 0.2 at week 24 (both p < 0.001, **Table 1**).

**Table 1:** Baseline demographic and clinical characteristics of screening cohort.

Characteristics	SLE patients (n=10)	
	Baseline	6-month treatment
Age, mean (SD), years	43 (14.7)	43 (14.7)
Female, n (%)	10 (100)	10 (100)
Duration of SLE disease, mean (SD), years	17.2 (12.6)	17.2 (12.6)
SLE disease activity		
Total SLEDAI-2K score, mean (SD)	13.7 (2.4)	2.4 (1.0)
PGA, mean (SD)	2.2 (0.5)	0.34 (0.21)
Clinical manifestations, (%)		
Musculoskeletal	7 (70)	0 (0)
Mucocutaneous	9 (90)	0 (0)
Cardiorespiratory	5 (50)	0 (0)
Hematological	4 (40)	3 (30)
Renal involvement, n (%)	0 (0%)	0 (0)
Immunological profile		
Anti-dsDNA antibodies positive, n (%)	9 (90)	5 (50)
Low complement (C3 and/orC4), n (%)	8 (80)	3 (30)
Treatment at baseline, n		
Glucocorticoids, n (%) Daily prednisone dose, mean (SD), mg/day	10 (100) 11.25 (7.6)	8 (80) 3.2 (1.3)
Antimalarial agents, n (%)	9 (90)	9 (90)
Immunosuppressants, n (%)	10 (100)	10 (100)
Mycophenolate mofetil	8 (80)	8 (80)
Azathioprine	1 (10)	1 (10)
Calcineurin inhibitors	1 (10)	0 (0)

SLE, Systemic lupus erythematosus; SLEDAI-2K, Systemic Lupus Erythematosus Disease Activity Index 2000; PGA, physician’s global assessment of disease activity. Reference ranges are as follows: anti-double-stranded DNA antibodies, <15 IU per milliliter; serum C3 (mg/dL), 85 to 110; serum C4 (mg/dL), 10 to 40.

**Figure 1:**
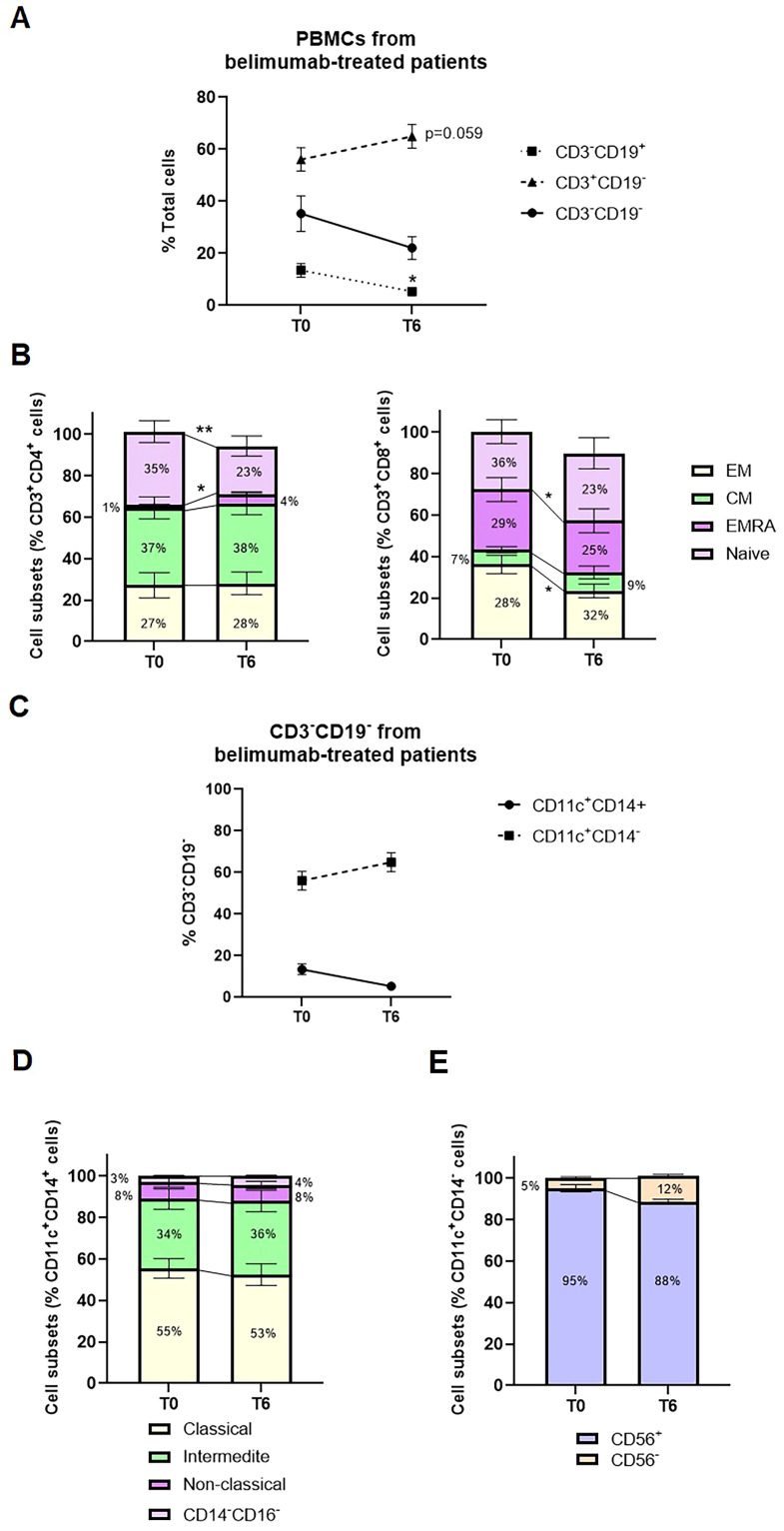
Median change from baseline to six months of belimumab treatment in PBMC subsets from SLE patients. **(A)** Changes in the percentage of CD3^-^CD19^+^ cells, CD3^+^CD19^-^ cells, and CD3^-^CD19^-^ cells between baseline (T0) and after six months of treatment (T6). Statistical analysis was performed using a one-way ANOVA with Student’s t-test. *p < 0.05. **(B)** Frequencies of T-helper and T-cytotoxic subsets, including Effector Memory (EM), Central Memory (CM), Effector Memory RA (EMRA), and Naïve subsets. Statistical analysis was performed using a one-way ANOVA with Student’s t-test. *p < 0.05, **p < 0.005. **(C)** Changes in the percentage of CD11c^+^CD14^+^ and CD11c^+^CD14^-^ cells within the CD3^-^CD19^-^ subset during belimumab treatment. **(D)** Frequencies of monocyte subsets categorized as Classical (CD14^+^CD16^+^), Non-classical (CD14^-^CD16^+^), Intermediate (CD14^+^CD16^-^), and Double Negative (CD14^-^CD16^-^). **(E)** Frequencies of CD56^+^ and CD56^-^ cells within the CD11c^+^CD14^-^ subset.

First, peripheral blood immunophenotyping was performed on PBMCs using flow cytometry, followed by cell sorting for subsequent miRNA analyses. Belimumab treatment resulted in a significant reduction in the percentage of CD3-CD19+ B cells (**Figure 1A**, p=0.013), without notable changes in B cell subtypes (**Supplementary Figure S2**). CD3+CD19− T cells showed a modest post-treatment increase (**Figure 1A**, p = 0.059, 56.1% vs. 64.9%). Whitin T-helper cells, there was a significant increase in effector memory T-helper cells re-expressing CD45RA (TEMRA) and a concurrent decrease in naïve T-helper cells (p = 0.031 and p = 0.006, respectively; **Figure 1B**). Among cytotoxic T cells, a pronounced reduction was observed in effector cells, particularly in the TEMRA and effector memory (TEM) subtypes, with fold change decreases of 1.16 and 1.5, respectively (**Figure 1B**).

Analysis of CD3−CD19− cells showed a predominant population of CD14+CD11c+ cells (70–85%), with a smaller subset of CD14−CD11c+ cells (15–30%). CD14+CD11c+ cells were mainly monocytes, further classified into classical (CD14+CD16−, 49–71%), intermediate (CD14+CD16+, 20–37%), and non-classical (CD14+CD16+, 3–13%) subpopulations, alongside a small proportion of dendritic cells (CD14−CD16−, <5%). In contrast, the CD14−CD11c+ subset was predominantly composed of CD56+ cells, representing to natural killer cells (85–95%). No significant changes in these cell types were observed post-treatment (**Figures 1C, D**).

### 3.2 mRNA gene expression profiling in distinct immune subsets associated with belimumab response

**Figure 2:**
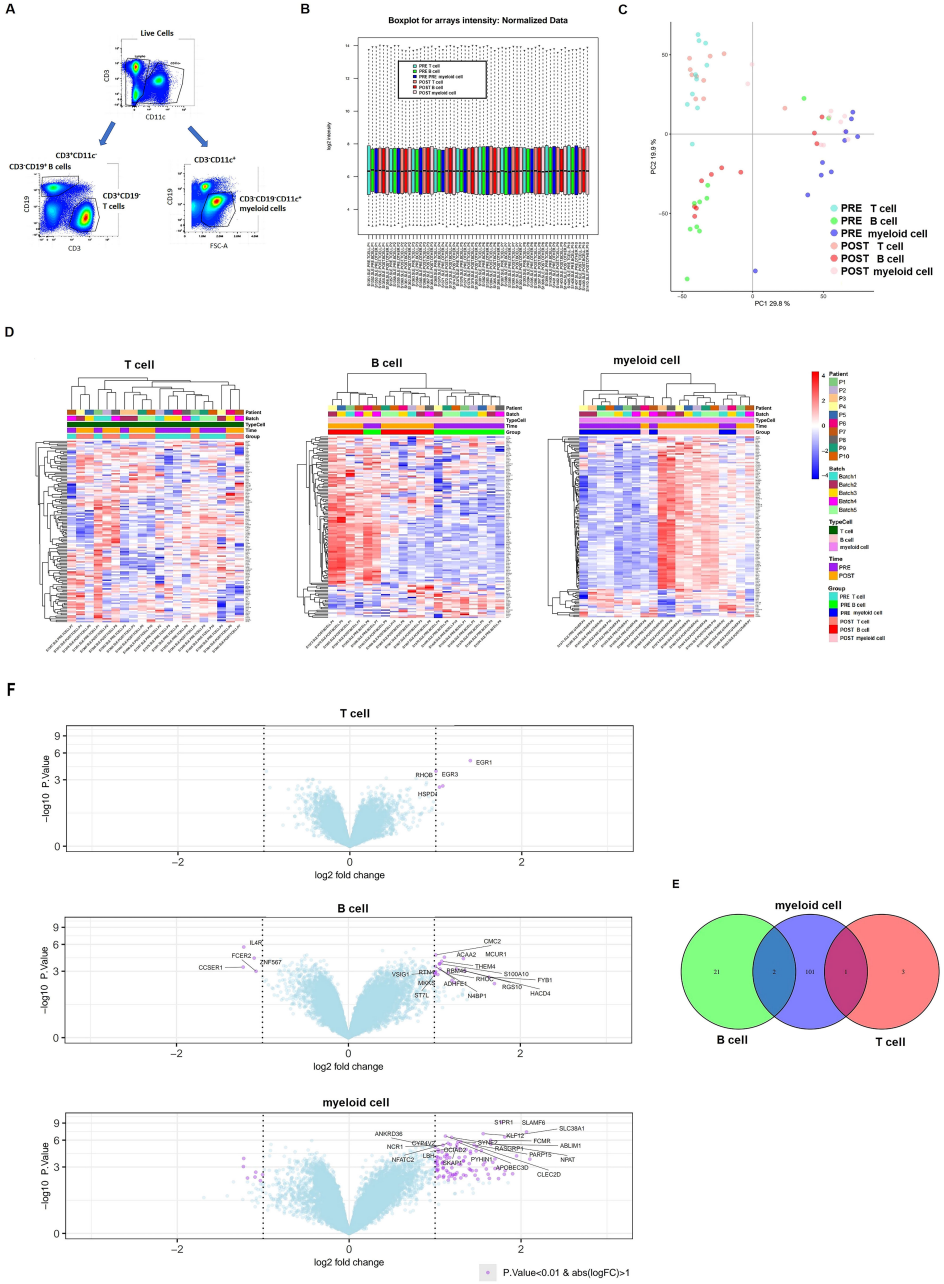
Differential expression of mRNA after six months of belimumab treatment in each immune subset. **(A)** Gating strategy used to identify the three immune subsets for transcriptomic analysis: CD3^+^CD19^-^ T cells, CD3^-^CD19^+^ B cells, and CD3^-^CD19^-^CD11c^+^ myeloid cells. **(B)** Boxplot of microarray intensity values after normalization. **(C)** Principal Component Analysis (PCA) distinguishing immune subsets (T cells, B cells, and myeloid cells) before (pre-treatment) and after (post-treatment) belimumab therapy. **(D, E)** Heatmap plots **(D)** and volcano plots **(E)** for each immune subset, showing differentially expressed genes (DEGs) pre- and post-belimumab treatment. In the volcano plots, the most significantly different genes are highlighted in purple (p-value < 0.01 and fold change > 1). **(F)** Venn diagram of DEGs identified in comparisons between pre- and post-treatment in T cells, B cells, and myeloid cells, illustrating the common genes among the subsets (p-value < 0.01 and fold change > 1).

We next analyzed mRNA expression differences across sorted CD3^–^CD19+ B cell, CD3+CD19− T cell and CD11c+CD3-CD19- myeloid cell subsets following belimumab treatment (**Figure 2A**). High-quality RNA was extracted from these subsets, enabling detailed transcriptome-wide profiling of over 20,800 full-length transcripts. Rigorous quality control measures ensured consistent and uniform distribution across all samples (**Figure 2B**, **Supplementary Table S2**).

Principal component analysis (PCA) initially grouped samples by cell subset, but incorporating adjustments for sample pairing improved the distinction between pre- and post-treatment samples (**Figure 2C**). Differential gene expression was assessed using a linear model with empirical Bayes moderation of variance, incorporating a fixed effect to account for patient variability. Using p-value < 0.01, this analysis identified 1,832 genes significantly associated with belimumab treatment, with the majority (62.9%; 1,153 genes) showing differential expression in the myeloid cell subset.

Post-treatment analysis revealed 625 differentially expressed genes in B cells (346 upregulated, 279 downregulated) and 1,153 genes myeloid cells (663 upregulated, 490 downregulated) (**Supplementary Table S4**, **S5**). In contrast, only 54 genes exhibited differential expression in T cells (**Supplementary Table S6**). Heatmaps demonstrated distinct expression patterns in these subsets between pre- and post-treatment samples (**Figure 2D**). Volcano plots illustrated the most significantly differentially expressed genes using a fold change > 1 and p-value < 0.01 (**Figure 2D**).

Venn diagram analysis showed no overlapping differentially expressed genes across all three cell subsets (**Figure 2E**). However, three genes were shared between subsets: CMC1 and THEM4 were commonly altered in B cells and myeloid cells, while HSPD1 was shared between T cells and myeloid cells.

### 3.3 Distinct miRNA expression profiles in immune cell subsets following belimumab therapy

**Figure 3:**
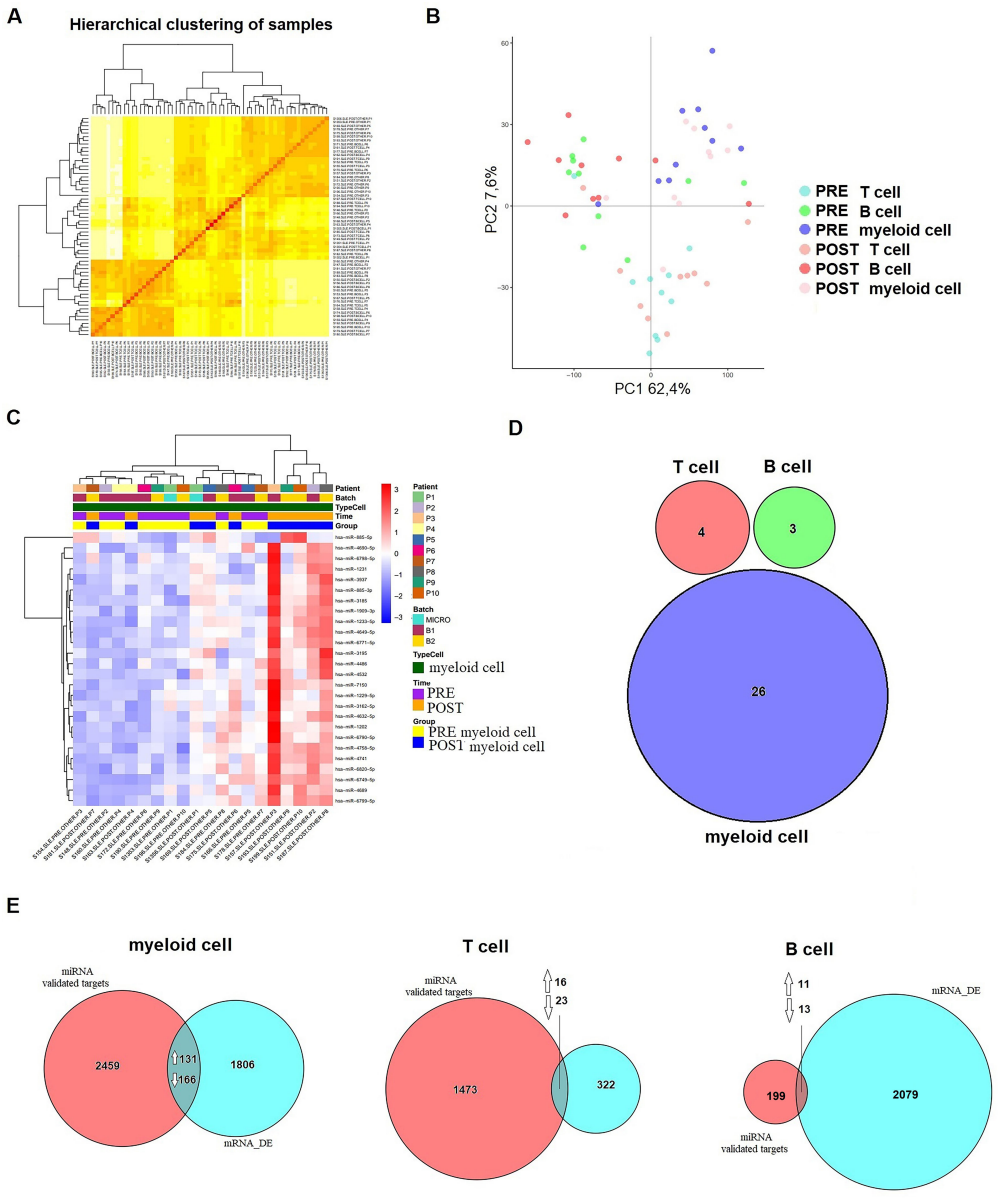
Differential expression of miRNA after six months of belimumab treatment in each immune subset. **(A)** Hierarchical clustering of samples demonstrating consistency and robust data across the samples. **(B)** Principal Component Analysis (PCA) distinguishing immune subsets (T cells, B cells, and myeloid cells) before (pre-treatment) and after (post-treatment) belimumab therapy. **(C)** Heatmap of myeloid cells showing clear differentiation based on treatment time (p < 0.05, |logFC| > 0.5). **(D)** Venn diagram comparing T cells, B cells, and myeloid cells revealed no common miRNAs among the subsets. **(E)** Analysis of validated targets of the identified miRNAs using databases such as miRecords, miRTarBase, and TarBase (validated mRNA targets) identified common genes between these targets and the DEGs from our microarray data (mRNA_DE). A Venn diagram illustrates these intersections. Arrows indicate whether the common genes are up- or down-regulated.

To evaluate treatment-induced changes in miRNA expression, we conducted microarray screening on T cells, B cells, and myeloid cells. After data filtering and normalizing, intensity distributions across samples demonstrated high consistency, ensuring robust results (**Figure 3A**). PCA revealed that the primary variation among samples was attributable to the cellular origin of the immune cell subsets (**Figure 3B**).

The differential expression analysis included 2,578 mature miRNA sequences and was performed using a linear model with empirical Bayes variance moderation. Using a raw p-value threshold of <0.01, we identified differentially expressed miRNAs within each immune subset before and after belimumab treatment. In T cells, 24 miRNAs were differentially expressed with 8 up-regulated and 16 down-regulated (**Supplementary Table S7**). In B cells, 19 miRNAs exhibited differential expressions, including 8 up-regulated and 11 down-regulated (**Supplementary Table S8**). The myeloid cell subset showed the most significant changes, with 36 differentially expressed miRNAs, comprising 24 up-regulated and 12 down-regulated miRNAs (**Supplementary Table S9**).

A heatmap revealed distinct expression patterns within the myeloid cell subset, where 26 miRNAs demonstrated significant changes (p < 0.05, |logFC| > 0.5) (**Figure 3C**). Comparison across immune subsets using a Venn diagram highlighted the lack of substantial overlap in differentially expressed miRNAs between pre- and post-treatment samples (**Figure 3D**).

### 3.4 miRNA target prediction and functional pathway analysis

To explore the genes targeted by differentially expressed miRNAs (p < 0.05, |logFC| > 0.5) and their corresponding changes in mRNA expression, we used the multiMiR Bioconductor package, focusing on validated targets from databases such as miRecords, miRTarBase, and TarBase. Only targets with robust experimental evidence (e.g., luciferase assays) were included in the analysis.

This approach identified 2,756 target genes in myeloid cells, 1,512 in T cells, and 223 in B cells. Venn diagram comparisons with differentially expressed genes (p < 0.05, |logFC| > 1) revealed overlaps across cell subsets. Specifically, in myeloid cells, 131 upregulated and 166 downregulated genes overlapped with miRNA targets. In T cells, 16 upregulated and 23 downregulated genes were identified, while B cells exhibited 11 upregulated and 13 downregulated overlapping genes (**Figure 3E**). Reactome pathway analysis highlighted enriched pathways associated with treatment response. Key pathways included metabolism in myeloid cells (p = 0.051), signal transduction in T cells (p = 0.016), and immune functions in B cells (p = 0.034).

### 3.5 Integrative analysis identified miRNA-mRNA interaction networks for belimumab treatment

**Figure 4:**
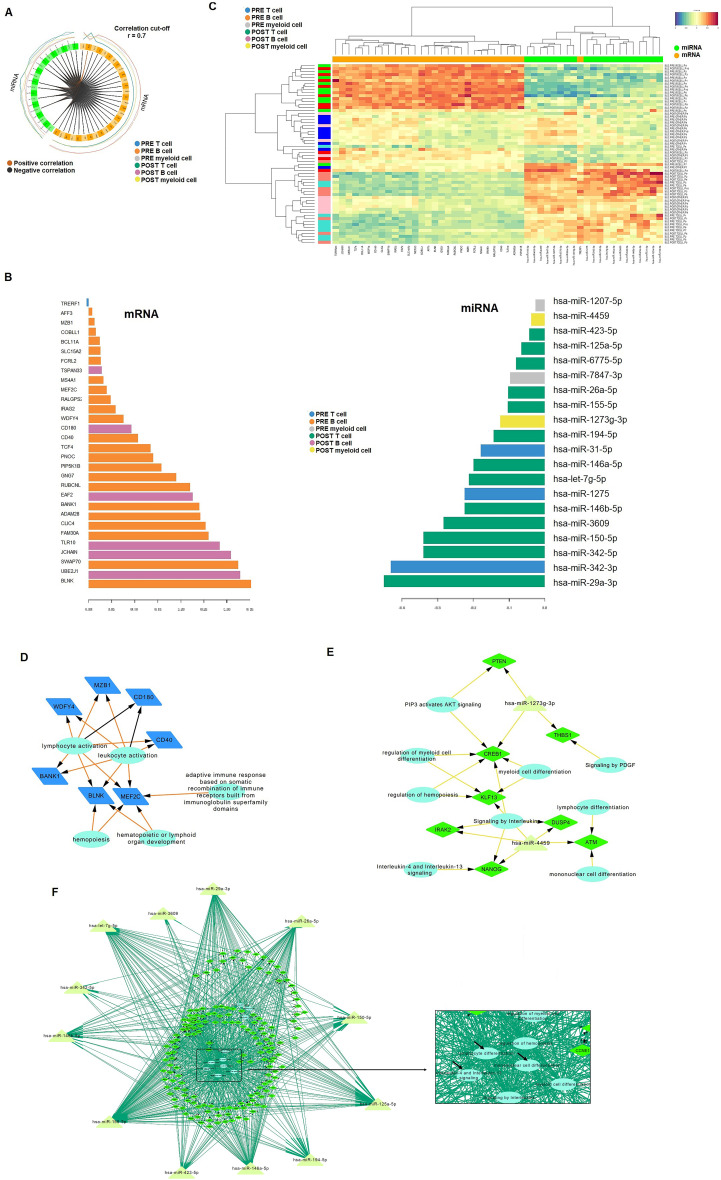
Integrative analysis of mRNA and miRNA data. **(A)** CircloPlot illustrating the correlation between differentially expressed genes (DEGs) and identified miRNAs from the two microarray datasets. **(B)** Principal Component 2 highlighting the most significant differences between samples based on treatment response. In the B cell subset, only mRNA was found to be relevant, while in T cells and myeloid cells, miRNAs were identified as significant contributors. **(C)** Heatmap showcasing that mRNA expression was predominantly associated with B cells, whereas miRNA expression was linked to T cells and myeloid cells. **(D-F)** Regulatory networks of DEGs (diamond) with relevant miRNAs (triangles) were constructed for B cells **(D)**, myeloid cells **(E)**, and T cells **(F)**. Reactome pathway analysis was incorporated into the networks, with biological pathways represented as circles.

We used DIABLO, a multivariate omics integration tool, to identify molecular interactions associated with belimumab treatment. Optimal feature selection for the mRNA and miRNA datasets was achieved using grid searches and leave-one-out cross-validation, yielding 50-40 mRNA features and 20 miRNA features per component, respectively. The explained variance was 14.4%, 11.3%, 9.4%, and 2.4% for mRNAs, and 9.4%, 4.9%, 2.0%, and 4.4% for miRNAs. A CircosPlot showed correlations among omics blocks in component 2, which exhibited the most distinct separation (**Figure 4A**, r > |0.70|).

Component 2 primarily captured the characteristics of B cells in the mRNA block and T cells/myeloid cells in the miRNA block (**Figure 4B**). The heatmap further highlighted that mRNA expression was predominantly associated with B cells, while miRNA expression was linked to T cells and myeloid cells (**Figure 4C**). Pre-treatment, 24 differentially expressed (DE) genes were identified in B cells, which decreased to six post-treatment. In T cells, 30 miRNAs emerged as top biomarkers post-treatment, compared to only three in pre-treatment (miR-342-3p, miR-1275, and miR-31-5p). In myeloid cells, miR-4459 and miR-1207-5p were upregulated post-treatment, whereas miR-1207-5p and miR-7847-3p were enriched pre-treatment.

Based on these results, we constructed an mRNA-miRNA-pathway network to visualize pathway interactions within each immune cell subset. Pre-treatment, genes such as MZB1, WDFY4, CD180, CD40, MEF2C, BLNK, and BANK1 were associated with lymphocyte activation and adaptive immune responses in B cells (**Figures 4D**). Post-treatment, CD180 remained significant in B cells, while a novel network of 7 genes and 2 miRNAs emerged in myeloid cells (**Figures 4E**). T cells exhibited a complex post-treatment network involving over 100 genes, associated with immune-related pathways, including interleukin signaling (e.g., IL-4 and IL-13), lymphocyte differentiation and mononuclear cell differentiation (**Figures 4F**).

### 3.6 Validation of integrative analysis to uncover the mechanism of action of belimumab in each immune cell type

**Figure 5:**
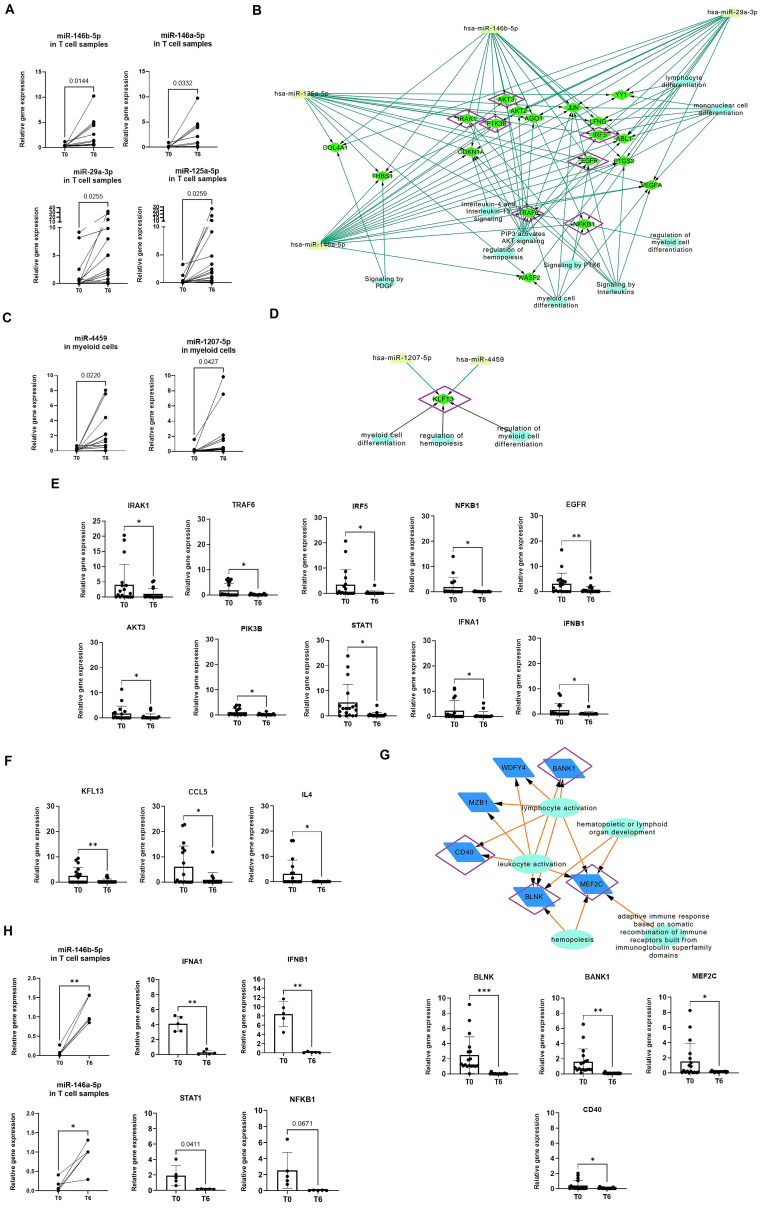
Validation of miRNA and gene expression using qPCR in a validation cohort for each immune subset. **(A, B)** miRNA expression was analyzed at baseline (T0) and after treatment (T6) in a new cohort of sorted T cells **(A)** and myeloid cells **(B)**. A Student’s t-test was performed to compare T0 with T6. *p < 0.05, **p < 0.005. **(C, D)** Networks were constructed using only the validated miRNAs and their common gene targets, which were identified as differentially expressed in the microarray analysis. For T cells **(C)**, 18 candidate genes were identified for validation, while for myeloid cells, only *KLF13* was found as a common target **(D)**. Genes validated by qPCR-RT are marked in purple. Reactome pathway analysis was incorporated into the networks, with biological pathways represented as circles. **(E, F)** Relative expression of candidate and related genes validated by qPCR-RT in T cells **(E)** and myeloid cells **(F)**. Relative gene expression was calculated using GAPDH as the control gene. Student’s t-test was used to compare T0 and T6. *p < 0.05, **p < 0.005. **(G)** Network of differentially validated genes from B cell subsets, with validated genes marked in purple. Student’s t-test was used to compare T0 and T6. *p < 0.05, **p < 0.005, ***p < 0.001. **(H)** Relative expression of miR-146a, miR-146b, IFNA1, IFNB1, and STAT1, which were found to be significantly changed in T cells isolated from a cohort of SLE patients in remission following immunosuppressive therapy (n = 5). No significant changes were observed in NFKB1, although a strong trend was observed with a p-value near 0.05. *p < 0.05, **p < 0.005, ***p < 0.001..

To validate the differentially expressed miRNAs identified in the mRNA-miRNA networks, qRT-PCR was performed on a new validation cohort of 18 belimumab-treated responder patients (**Table 2**). In T cells, miR-125a-5p, miR-146b-5p, miR-146a-5p, and miR-29a-3p were confirmed to be upregulated post-treatment (fold changes of 15.2, 5.9, 16.8, and 5.1, respectively, **Figure 5A**). In myeloid cells, miR-1207-5p and miR-4459 showed significant upregulation post-treatment, with fold increases of 11.1 (p = 0.043) and 16.9 (p = 0.022), respectively (**Figure 5B**). Clinical data from pre- and post-treatment samples (anti-dsDNA titers, complement values, and SLEDAI and PGA scores) were correlated with miRNA expressions; however, no significant correlations were found (**Supplementary Figure S3**). Using these validated miRNAs, we constructed *de novo* mRNA-miRNA-pathway networks, which highlighted common mRNA targets specific to each immune cell subset (**Figures 5C, D**). These candidate mRNA genes were further validated by qRT-PCR. In T cells, post-treatment validation revealed significant downregulation of several genes from pathway networks, including *IRAK1, TRAF6, IRF5* and *NFKB1* (fold decrease of 4.22, 12.7, 15.5 and 36.5, respectively). *EGFR* was also downregulated along with *PIK3B* and *AKT3* (fold decrease of 4.4, 4.8 and 3.6, respectively, **Figure 5E**). Related genes from this network were also studied such as interferon, MAPKs/JAKs/STATs or survival genes, but only *STAT1, IFNA* and *IFNB* were found significantly downregulated (fold decrease of 12.7, 4.2 and 7.25, respectively, **Figure 5E**). These findings suggest that belimumab modulates inflammation through the regulation of IFN and NF-κB signaling pathways in T cells.

**Table 2:** Baseline demographic and clinical characteristics of validation cohort.

Characteristics	SLE patients (n=18)	
	Baseline	6-month treatment
Age, mean (SD), years	44 (9.4)	44 (9.4)
Female, n (%)	15 (83)	15 (83)
Duration of SLE disease, mean (SD), years	12.5 (4.6)	12.5 (4.6)
SLE disease activity		
Total SLEDAI-2K score, mean (SD)	12.8 (2.6)	2.5 (1.5)
PGA, mean (SD)	2.02 (0.43)	0.30 (0.18)
Clinical manifestations, (%)		
Musculoskeletal	13 (72)	0 (0)
Mucocutaneous	15 (83)	0 (0)
Cardiorespiratory	5 (28)	0 (0)
Hematological	6 (33)	3 (30)
Renal involvement, n (%)	0 (0)	0 (0)
Immunological profile		
Anti-dsDNA antibodies positive, n (%)	17 (94)	12 (66)
Low complement (C3 and/orC4), n (%)	13 (72)	6 (33)
Treatment at baseline, n		
Glucocorticoids, n (%) Daily prednisone dose, mean (SD), mg/day	18 (100) 10.35 (9.2)	16 (88.9) 4.2 (2.2)
Antimalarial agents, n (%)	16 (89)	13 (72)
Immunosuppressants, n (%)	17 (94)	17 (94)
Mycophenolate mofetil	15 (83)	15 (83)
Azathioprine	2 (11)	2 (11)

SLE, Systemic lupus erythematosus; SLEDAI-2K, Systemic Lupus Erythematosus Disease Activity Index 2000; PGA, physician’s global assessment of disease activity. Reference ranges are as follows: anti-double-stranded DNA antibodies, <15 IU per milliliter; serum C3 (mg/dL), 85 to 110; serum C4 (mg/dL), 10 to 40.

In myeloid cells, *KLF3* was downregulated post-treatment (fold change of 6.47, **Figure 5F**). KLF3 is known to regulate the production of cytokines, including *CCL5, CXCL10, IL4, TNFA*, and *IFNG.* We confirmed the downregulation of *CCL5* and *IL4*, with fold decreases of 6.5 and 8.8, respectively (**Figure 5F**). This supports the role of *KLF13* in modulating cytokine expression in myeloid cells.

Finally, in B cells, post-treatment validation confirmed the significant downregulation of *BLNK, BANK1, MEF2C* and *CD40* (p=0.005, 0.002, 0.026 and 0.049, respectively, **Figure 5G**). These genes are intricately involved in B cell development and the regulation of BCR, BAFFR, and NF-κB pathways.

To determine whether the observed changes in miRNA and mRNA expression were specifically associated with belimumab treatment, we included a cohort of SLE patients in remission following immunosuppressive therapy (n = 5, **Supplementary Table S10**). In this group, no significant differences were observed in miR-125a-5p and miR-29a-3p expression in isolated T cells, nor in miR-1207-5p and miR-4459 expression in myeloid cells (**Supplementary Figure S4**).However, a significant upregulation of miR-146b-5p and miR-146a-5p was detected in T cells (p = 0.0016 and 0.016, respectively; **Figure 5H**). Gene expression analysis further revealed a significant decrease in IFNA1, IFNB1, and STAT1 in T cells (p = 0.0021, 0.0024, and 0.0458, respectively, **Figure 5H**), with a trend toward a reduction in NFKB1 expression (p = 0.0671). Notably, no significant changes were detected in myeloid cells or B cells.

## 4 Discussion

This study provides the first systematic investigation of miRNAs involved in the mechanism of action belimumab, using an integrative analysis of miRNA and mRNA expression profiles across distinct PBMC subsets from SLE patients who responded to treatment. Our analysis identified 1,832 genes and 79 miRNAs significantly associated with treatment response, revealing 525 miRNA-gene interactions. Pathway enrichment analysis highlights transcriptional signatures related to both innate and adaptive immunity.

Previous studies using flow and mass cytometry consistently report an overall decrease in B cells after belimumab treatment, with no significant effects on T cells. After six months of treatment, these studies show a rapid and sustained reduction in naïve and total B cells, while plasma cells and switched memory B cells remain stable (24–26). In our study, we observed a general reduction in total B-cell counts but no significant changes in the proportions of different B-cell subsets after six months. Notably, we observed a significant increase in effector memory T-helper cells and a reduction in effector memory cytotoxic T cells, which contrasts with prior findings. Additionally, myeloid cell analysis revealed no significant changes in their subsets over time.

A key finding of our study was the identification of miRNA profiles specific to distinct PBMC subsets in response to belimumab. Using integrative analysis and subsequent validation by qRT-PCR, we identified differentially expressed miRNAs in T cells and myeloid cells, while no significant changes were observed in B cells. Instead, transcriptomic analysis revealed downregulation of B-cell-related genes such as *BLNK, MEF2C, BANK1*, and the *CD40* co-receptor, critical for B-cell development and survival (27, 28).

T cells play a central role in SLE pathogenesis by amplifying inflammation through the secretion of pro-inflammatory cytokines, facilitating autoantibody production, and driving disease progression via the accumulation of autoreactive memory T cells (29). Increasing evidence indicates that miRNAs regulate aberrant T cell behavior in SLE, modulating key pathogenic mechanisms such as activation of the interferon pathway, DNA hypomethylation, CD40L surface expression, Th17/Treg imbalance, and IL-2 reduction (30). In our study, we identified four miRNAs (miR-146b-5p, miR-146a-5p, miR-125a-5p, and miR-29a-3p) upregulated in T cells following belimumab treatment.

The miR-146 family, comprising miR-146a and miR-146b, plays a critical role in maintaining immune homeostasis and regulating both innate and adaptive immune responses (31, 32). These miRNAs are induced by lipopolysaccharides (LPS) via the NF-κB signaling pathway and act as negative regulators of pathway activity through Toll-like receptor 4 (TLR4). In SLE patients, miR-146a is consistently downregulated in PBMCs and CD4+ T cells, with its expression inversely correlated with disease activity and interferon scores (33). Our study demonstrated that post-treatment T cells exhibited a decrease in interferon-related gene expression (*INFA, INFB, STAT1*), which correlated with the upregulation of miR-146a. The significance of miR-146a in SLE pathogenesis is further supported by studies demonstrating that miR-146a-deficient mice develop an SLE-like phenotype, characterized by hyperresponsive T cells, increased expression of activation markers such as CD69 and CD44, and enhanced cytokine production, including IFN-γ and IL-17. Moreover, *in vitro* and *in vivo* studies have demonstrated that miR-146a overexpression inhibits T cell activation by targeting key proteins in the type I and II interferon and NF-κB signaling pathways, including IRF-5, STAT-1, TRAF6, and IRAK1 (34–36). Consistently, our study observed downregulation of *TRAF6, NFKB1, IRAK1*, and *IRF5* in T cells post-treatment. Interestingly, treatment with mycophenolic acid, a widely used immunosuppressive therapy for SLE, has been shown to increase miR-146a levels (37). In line with these findings, miRNA expression analysis in SLE patients treated with mycophenolate mofetil revealed an upregulation of miR-146a and miR-146b following treatment, accompanied by a significant downregulation of IFNA, IFNB, and STAT1, as well as a trend toward downregulation of NFKB1, all of which are known targets of miR-146a. These results suggest that miR-146a and its target genes are more closely linked to clinical response rather than being specific to belimumab treatment, highlighting miR-146a as a potential biomarker for therapeutic response in SLE (38).

We also observed upregulation of miR-125a-5p and miR-29a-3p in T cells, both of which target EGFR, a key regulator of T cell survival and proliferation. EGFR activation triggers downstream signaling pathwayssuch as PI3K/Akt, MAPK, and JAK/STAT (39). The reduced expression of EGFR and its downstream effectors, including *AKT3, PI3KB, STAT1* and *IRF5*, suggests that belimumab may also inhibit T cell proliferation and cytokine release through this pathway. Additionally, miR-125a-5p and miR-29a-3p may contribute to the suppression of NF-κB and interferon signaling by targeting TRAF6/IRF5 (40) or by regulating T-box transcription factors (41), further enhancing the effects of miR-146.Belimumab also influenced myeloid cells by increasing the levels of miR-1207-5p and miR-4459, both of which target *KLF13*, a key regulator of cytokine signaling in response to toll-like receptor (TLR) ligands (42) and CCL5 production, a chemokine critical for immune cell recruitment and activation (43). In our study, the downregulation of *KLF13* and *CCL5* in myeloid cells, alongside reduced *IL4* expression, suggests a shift away from an inflammatory phenotype and suppression of Th2 cell differentiation. Th2 cells contribute to SLE pathogenesis by promoting autoreactive IgE plasma cell generation and activating plasmacytoid dendritic cells, which sustain inflammation (44–46). miRNA analysis of the non-belimumab-treated cohort showed no significant changes in miR-125a-5p, miR-29a-3p, miR-1207-5p, and miR-4459 expression in T cells and myeloid cells, respectively. These findings suggest that, in addition to its well-established effects on B cells, belimumab specifically modulates key immune pathways in T cells and myeloid cells, further supporting its targeted role in immune regulation in SLE.

**Figure 6:**
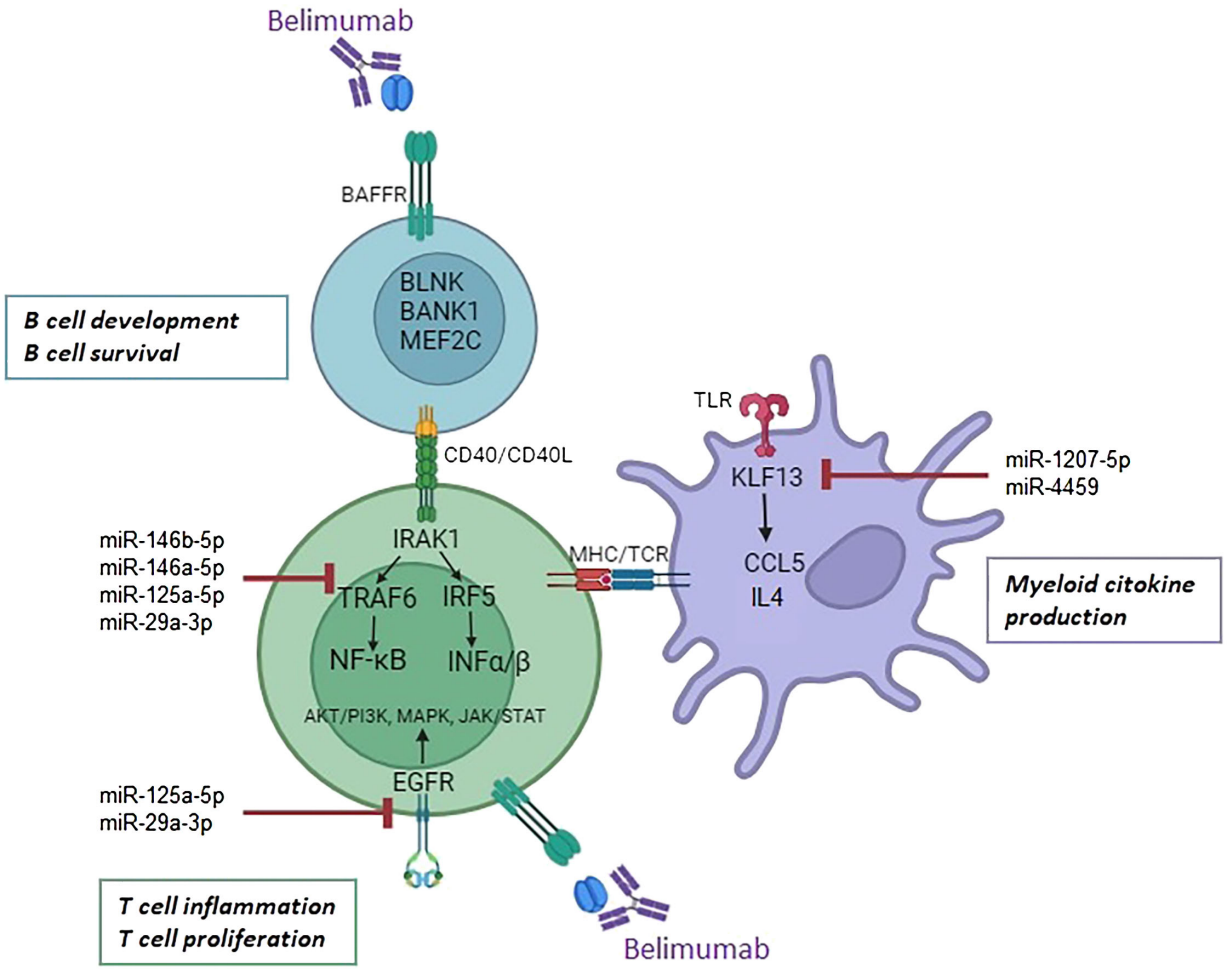
Proposed mechanism of action of belimumab in B cells, T cells, and myeloid cells. Belimumab impacts B cell development and survival through modulation of *BLNK, BANK1, MEF2C*, and the *CD40* receptor. Its effects may regulate miRNA expression in T cells, targeting the interferon and NF-ĸB pathways via the downregulation of *IRAK1, TRAF6, IRF5*, and *EGFR*. In myeloid cells, miR-1207-5p and miR-4459 target *KLF13*, leading to the downregulation of inflammatory cytokines *CCL5* and *IL4*.

Our findings align with previous studies investigating belimumab’s molecular mechanisms, which highlight its ability to restore the Treg/Th17 balance by reducing serum levels IL-21 and downregulate multiple immune pathways, including those related to B cell activation, type I and type II interferon signaling, and IL-6/STAT3 (47, 48). Additionally, Belimumab has been shown to reduce neutrophil activation, further enhancing its therapeutic effect. By targeting BAFF, belimumab suppresses autoreactive B cell survival and autoantibody production, modulating T cells, neutrophils, and dendritic cells (49). Collectively, these findings highlight the multi-faceted mechanisms of belimumab in modulating both innate and adaptive immune responses, reinforcing its role as a pivotal therapy in SLE management. In this study, we integrated mRNA and miRNA transcriptomic analyses to investigate interactions among B cells, T cells, and myeloid cells. Our results demonstrate that belimumab modulates key B cell genes such as *BLNK, BANK1, MEF2C*, as well as the *CD40* receptor, influencing T cell interactions. The CD40/CD40L axis is crucial for B cell differentiation into plasmablasts, with NF-κB signaling acting as a downstream mediator through TRAF protein recruitment (50). Belimumab also reshapes T cell epigenetic regulation, influencing miRNAs involved in interferon signaling and pro-inflammatory cytokines via NF-κB activation. Furthermore, T cells in SLE patients express BAFFR, suggesting direct effects of belimumab on T cells (51). Finally, the observed changes in myeloid cells may be secondary to T cell modulation, as T cells orchestrate both humoral and cellular immune responses (52) (**Figure 6**).

This study has some limitations, including a small sample size and a focus on SLE responders excluding patients with renal involvement. Further research is needed to investigate these effects in non-responders, in lupus nephritis patients, and through the longitudinal evaluation of miRNAs expression. Additionally, validating the observed immune cell interactions *in vitro* is crucial. Despite these limitations, our study is the first to perform transcriptomic analysis of isolated immune cell subsets, highlighting the role of miRNAs in modulating immune responses in SLE. These findings suggest that miRNAs may serve as valuable biomarkers for assessing therapeutic response and could be promising targets for optimizing treatment outcomes.

## Data Availability

The datasets presented in this study can be found in online repositories. The names of the repository/repositories and accession number(s) can be found in the article/**Supplementary Material**. The microarray data is deposited in the GEO database at NCBI under the accession number GSE283865.

## References

[1] Pan L Lu M-P Wang J-H Xu M Yang S-R. Immunological pathogenesis and treatment of systemic lupus erythematosus. World J Pediatr. (2020) 16:19–30. doi: 10.1007/s12519-019-00229-3 30796732 PMC7040062

[2] Davidson A. The rationale for BAFF inhibition in systemic lupus erythematosus. Curr Rheumatol Rep. (2012) 14:295–302. doi: 10.1007/s11926-012-0258-2 22535567 PMC3389191

[3] Cheema GS Roschke V Hilbert DM Stohl W. Elevated serum B lymphocyte stimulator levels in patients with systemic immune-based rheumatic diseases. Arthritis Rheumatol. (2001) 44:1313–9. doi: 10.1002/1529-0131(200106)44:6<1313::AID-ART223>3.0.CO;2-S 11407690

[4] Steri M Orrù V Idda ML Pitzalis M Pala M Zara I. Overexpression of the cytokine BAFF and autoimmunity risk. N Engl J Med. (2017) 376:1615–26. doi: 10.1056/NEJMoa1610528 PMC5605835 28445677

[5] Mackay F Woodcock SA Lawton P Ambrose C Baetscher M Schneider P. Mice transgenic for BAFF develop lymphocytic disorders along with autoimmune manifestations. J Exp Med. (1999) 190:1697–710. doi: 10.1084/jem.190.11.1697 PMC2195729 10587360

[6] Gross JA Dillon SR Mudri S Johnston J Littau A Roque R. TACI-Ig neutralizes molecules critical for B cell development and autoimmune disease. Immunity. (2001) 15:289–302. doi: 10.1016/S1074-7613(01)00183-2 11520463

[7] Blair HA Duggan ST. Belimumab: A review in systemic lupus erythematosus. Drugs. (2018) 78:355–66. doi: 10.1007/s40265-018-0872-z 29396833

[8] Navarra SV Guzmán RM Gallacher AE Hall S Levy RA Jimenez RE. Efficacy and safety of belimumab in patients with active systemic lupus erythematosus: a randomized, placebo-controlled, phase 3 trial. Lancet. (2011) 377:721–31. doi: 10.1016/S0140-6736(10)61354-2 21296403

[9] Furie R Petri M Zamani O Cervera R Wallace DJ Tegzová D. A Phase 3, randomized, placebo-controlled study of belimumab, a monoclonal antibody that inhibits BLyS, in patients with systemic lupus erythematosus. Arthritis Rheumatol. (2011) 63:3918–30. doi: 10.1002/art.30613 PMC5007058 22127708

[10] Ceribelli A Satoh M Chan EKL. MicroRNAs and autoimmunity. Curr Opin Immunol. (2012) 24:686–91. doi: 10.1016/j.coi.2012.07.011 PMC3508200 22902047

[11] Choi D Kim J Yang JW Kim JH Park S Shin JI. Dysregulated microRNAs in the pathogenesis of systemic lupus erythematosus: a comprehensive review. Int J Biol Sci. (2023) 19:2495–514. doi: 10.7150/ijbs.74315 PMC10197884 37215992

[12] Wu XN Ye YX Niu JW Li Y Li X You X. Defective PTEN regulation contributes to B cell hyperresponsiveness in systemic lupus erythematosus. Sci Transl Med. (2014) 6:246ra99. doi: 10.1126/scitranslmed.3009131 25101889

[13] van den Hoogen LL Rossato M Lopes AP Pandit A Bekker CPJ Fritsch-Stork RDE. MicroRNA downregulation in plasmacytoid dendritic cells in interferon-positive systemic lupus erythematosus and antiphospholipid syndrome. Rheumatol (Oxford). (2018) 57:1669–74. doi: 10.1093/rheumatology/key159 29873766

[14] Fanouriakis A Kostopoulou M Alunno A Aringer M Bajema I Boletis JN. 2019 update of the EULAR recommendations for the management of systemic lupus erythematosus. Ann Rheum Dis. (2019) 78:736–45. doi: 10.1136/annrheumdis-2019-215089 30926722

[15] Franklyn K Lau C Navarra SV Louthrenoo W Lateef A Homijoyo L. Definition and initial validation of a Lupus Low Disease Activity State (LLDAS). Ann Rheum Dis. (2016) 75:1615–21. doi: 10.1136/annrheumdis-2015-207726 26458737

[16] Bioconductor. Bioconductor project (2024). Available online at: https://www.bioconductor.org (Accessed November 26, 2024).

[17] Gentleman RA Carey VJ Huber W Irizarry RA Dudoit S. Bioinformatics and Computational Biology Solutions using R and Bioconductor. New York: Springer (2005).

[18] MultiMiR. MultiMiR Database (2024). Available online at: http://multimir.ucdenver.edu (Accessed November 26, 2024).

[19] Smyth GK. Linear models and empirical Bayes methods for assessing differential expression in microarray experiments. Stat Appl Genet Mol Biol. (2004) 3:Article 3. doi: 10.2202/1544-6115.1027 16646809

[20] Lê Cao KA Boitard S Besse P. Sparse PLS discriminant analysis: biologically relevant feature selection and graphical displays for multiclass problems. BMC Bioinf. (2011) 12:253. doi: 10.1186/1471-2105-12-253 PMC3133555 21693065

[21] Yu G Wang LG Han Y He QY. clusterProfiler: an R package for comparing biological themes among gene clusters. OMICS. (2012) 16:284–7. doi: 10.1089/omi.2011.0118 PMC3339379 22455463

[22] Milacic M Beavers D Conley P Gong C Gillespie M Griss J. The reactome pathway knowledgebase 2024. Nucleic Acids Res. (2024) 52:D672–8. doi: 10.1093/nar/gkad1025 PMC10767911 37941124

[23] Shannon P Markiel A Ozier O Baliga NS Wang JT Ramage D. Cytoscape: a software environment for integrated models of biomolecular interaction networks. Genome Res. (2003) 13:2498–504. doi: 10.1101/gr.1239303 PMC403769 14597658

[24] Stohl W Hiepe F Latinis KM Thomas M Scheinberg MA Clarke A. Belimumab reduces autoantibodies, normalizes low complement levels, and reduces select B cell populations in patients with systemic lupus erythematosus. Arthritis Rheumatol. (2012) 64:2328–37. doi: 10.1002/art.34400 PMC3350827 22275291

[25] Furie RA Wallace DJ Aranow C Fettiplace J Wilson B Mistry P. Long-term safety and efficacy of belimumab in patients with systemic lupus erythematosus: A continuation of a seventy-six-week phase III parent study in the United States. Arthritis Rheumatol. (2018) 70:868–77. doi: 10.1002/art.40439 PMC6001779 29409143

[26] Ramsköld D Parodis I Lakshmikanth T Sippl N Khademi M Chen Y. B cell alterations during BAFF inhibition with belimumab in SLE. EBioMedicine. (2019) 40:517–27. doi: 10.1016/j.ebiom.2018.12.035 PMC6412067 30593436

[27] Tsukada S Baba Y Watanabe D. Btk and BLNK in B cell development. Adv Immunol. (2001) 77:123–62. doi: 10.1016/s0065-2776(01)77016-2 11293115

[28] Kong NR Davis M Chai L Winoto A Tjian R. MEF2C and EBF1 co-regulate B cell-specific transcription. PloS Genet. (2016) 12:e1005845. doi: 10.1371/journal.pgen.1005845 26900922 PMC4762780

[29] Gómez Hernández G Morell M Alarcón-Riquelme ME. The role of BANK1 in B cell signaling and disease. Cells. (2021) 10:1184. doi: 10.3390/cells10051184 34066164 PMC8151866

[30] Tenbrock K Rauen T. T cell dysregulation in SLE. Clin Immunol. (2022) 239:109031. doi: 10.1016/j.clim.2022.109031 35526790

[31] Lai N-S Koo M Yu C-L Lu M-C. Immunopathogenesis of systemic lupus erythematosus and rheumatoid arthritis: the role of aberrant expression of non-coding RNAs in T cells. Clin Exp Immunol. (2017) . 187:327–36. doi: 10.1111/cei.12903 PMC5290235 27880973

[32] Testa U Pelosi E Castelli G Labbaye C. miR-146 and miR-155: two key modulators of immune response and tumor development. Noncoding RNA. (2017) 3:22. doi: 10.3390/ncrna3030022 29657293 PMC5831915

[33] Mortazavi-Jahromi SS Aslani M Mirshafiey A. A comprehensive review on miR-146a molecular mechanisms in a wide spectrum of immune and non-immune inflammatory diseases. Immunol Lett. (2020) 227:8–27. doi: 10.1016/j.imlet.2020.07.008 32810557

[34] Zhu X Zhang Y Yin Z Ye Z Qin Y Cheng Z. Three-dimensional chromosomal landscape revealing miR-146a dysfunctional enhancer in lupus and establishing a CRISPR-mediated approach to inhibit the interferon pathway. Arthritis Rheumatol. (2024) 76:384–95. doi: 10.1002/art.42703 37728419

[35] Li B Wang X Choi IY Wang Y-C Liu S Pham AT. miR-146a modulates autoreactive Th17 cell differentiation and regulates organ-specific autoimmunity. J Clin Invest. (2017) 127:3702–16. doi: 10.1172/JCI94012 PMC5617680 28872459

[36] Tang Y Luo X Cui H Ni X Yuan M Guo Y. MicroRNA-146A contributes to abnormal activation of the type I interferon pathway in human lupus by targeting key signaling proteins. Arthritis Rheumatol. (2009) 60:1065–75. doi: 10.1002/art.24436 19333922

[37] Yang L Boldin MP Yu Y Liu CS Ea CK Ramakrishnan P. miR-146a controls the resolution of T cell responses in mice. J Exp Med. (2012) 209:1655–70. doi: 10.1084/jem.20112218 PMC3428948 22891274

[38] Tang Q Yang Y Zhao M Liang G Wu H Liu Q. Mycophenolic acid upregulates miR-142-3P/5P and miR-146a in lupus CD4+ T cells. Lupus. (2015) 24:935–42. doi: 10.1177/0961203315570685 25661834

[39] Sabbah DA Hajjo R Sweidan K. Review on epidermal growth factor receptor (EGFR) structure, signaling pathways, interactions, and recent updates of EGFR inhibitors. Curr Top Med Chem. (2020) 20:815–34. doi: 10.2174/1568026620666200303123102 32124699

[40] Gong ZT Xiong YY Ning Y Tang RJ Xu JY Jiang WY. Nicorandil-pretreated mesenchymal stem cell-derived exosomes facilitate cardiac repair after myocardial infarction via promoting macrophage M2 polarization by targeting miR-125a-5p/TRAF6/IRF5 signaling pathway. Int J Nanomed. (2024) 19:2005–24. doi: 10.2147/IJN.S441307 PMC10926597 38469055

[41] Steiner DF Thomas MF Hu JK Yang Z Babiarz JE Allen CDC. MicroRNA-29 regulates T-box transcription factors and interferon-γ production in helper T cells. Immunity. (2011) 35:169–81. doi: 10.1016/j.immuni.2011.07.009 PMC3361370 21820330

[42] Wang A Fairhurst A-M Liu K Wakeland B Barnes S Malladi VS. KLF13 promotes SLE pathogenesis by modifying chromatin accessibility of key proinflammatory cytokine genes. Commun Biol. (2024) 7:1446. doi: 10.1038/s42003-024-04471-y 39506084 PMC11541912

[43] Huang B Ahn Y-T McPherson L Clayberger C Krensky AM. Interaction of PRP4 with Kruppel-like factor 13 regulates CCL5 transcription. J Immunol. (2007) 178:7081–7. doi: 10.4049/jimmunol.178.11.7081 PMC2674583 17513757

[44] Charles N Hardwick D Daugas E Illei GG Rivera J. Basophils and the T helper 2 environment can promote the development of lupus nephritis. Nat Med. (2010) 16:701–7. doi: 10.1038/nm.2159 PMC2909583 20512127

[45] Kim CJ Lee CG Jung JY Ghosh A Hasan SN Hwang SM. The transcription factor Ets1 suppresses T follicular helper type 2 cell differentiation to halt the onset of systemic lupus erythematosus. Immunity. (2018) 49:1034–48.e1038. doi: 10.1016/j.immuni.2018.10.012 30566881

[46] Ko H Kim CJ Im S-H. T helper 2-associated immunity in the pathogenesis of systemic lupus erythematosus. Front Immunol. (2022) 13:866549. doi: 10.3389/fimmu.2022.866549 35444658 PMC9014558

[47] Prete M Lone P Frassanito MA Desantis V Marasco C Cicco S. Belimumab restores Treg/Th17 balance in patients with refractory systemic lupus erythematosus. Lupus. (2018) . 27:1926–35. doi: 10.1177/0961203318797425 30180771

[48] Moysidou G-S Garantziotis P Sentis G Nikoleri D Malissovas N Nikoloudaki M. Moleclar basis for the disease-modifying effects of belimumab in systemic lupus erythematosus and molecular predictors of early response: blood transcriptome analysis implicates the innate immunity and DNA damage response pathways. Ann Rheum Dis. (2024) 13:262–73. doi: 10.1136/ard-2024-226051. ard-2024-226051. 39919899

[49] Zhang Y Tian J Xiao F Zheng L Zhu X Wu L. B cell-activating factor and its targeted therapy in autoimmune diseases. Cytokine Growth Factor Rev. (2022) 64:57–70. doi: 10.1016/j.cytogfr.2021.11.004 34916133

[50] Elgueta R Benson MJ de Vries VC Wasiuk A Guo Y Noelle RJ. Molecular mechanism and function of CD40/CD40L engagement in the immune system. Immunol Rev. (2009) 229:111–26. doi: 10.1111/j.1600-065X.2009.00782.x PMC3826168 19426221

[51] Sagrero-Fabela N Ortíz-Lazareno PC Salazar-Camarena D Cruz A Cerpa-Cruz S Muñoz-Valle JF. BAFFR expression in circulating T follicular helper (CD4+CXCR5+PD-1+) and T peripheral helper (CD4+CXCR5–PD-1+) cells in systemic lupus erythematosus. Lupus. (2024) 32:1201–12. doi: 10.1177/09612033231189804 37460408

[52] Lee H-G Cho M-J Choi J-M. Bystander CD4+ T cells: crossroads between innate and adaptive immunity. Exp Mol Med. (2020) 52:1255–63. doi: 10.1038/s12276-020-0483-6 PMC8080565 32859954

