# Supplementary material for: Integrative miRNA-mRNA profiling uncovers mechanisms of belimumab action in systemic lupus erythematosus
Source: Front Immunol. 2025 Mar 14;16:1553971. doi: 10.3389/fimmu.2025.1553971 (PMC11949941; doi:10.3389/fimmu.2025.1553971)
Supplement: Supplementary file 1 [file DataSheet1.pdf]

Supplementary Material

Integrative miRNA-mRNA Profiling Uncovers Mechanisms of Belimumab Action in Systemic Lupus Erythematosus

Maria Royo[1], Blanca Joseph-Mullol[1], Sebastian Sandoval[1], Teresa Moliné[2], Cristina Solé[1,*], Josefina Cortés-Hernández[1]

[1] Rheumatology Research group, Lupus Unit, Hospital Universitari Vall d'Hebron, Institut de Recerca (VHIR), Universitat Autònoma de Barcelona, 08035 Barcelona, Spain

[2] Department of Pathology, Hospital Universitari Vall d'Hebron, Institut de Recerca (VHIR), Universitat Autònoma de Barcelona, 08035 Barcelona, Spain

INDEX

1 Supplementary Data 3
2 References..5
3 Supplementary Figures 6
4 Supplementary Tables...11

1 Supplementary Data

1.1 Flow Cytometry Analysis

An 17-color flow cytometry panel was developed to identify immune cell population in PBMCs samples from patients. Antibodies include anti-CD3-PE (SK7), anti-CD4-FITC (RPA-T4), ANTI-CD8-APC (RPA-T8), anti-CD19-PE-Cy7 (SJ25C1), anti-CD56-BB700 (NCAM16.2), anti-CD11c-BV650 (B-ly6), anti-CD68-PECF594 (Y1/82A), anti-CCR7-BV421 (150503), anti-IgD-R71 (IA6-2), anti-CD27-BV76 (L128) (all BD Bioscienes), anti-CD45RA-PerCPCy55 (HI100), anti-CD38-APC-Fire810 (HIT2), anti-CD127-BV711 (A019D5), anti-CD25-BV510 (M-A251), anti-CD16-PacificBlue (3G8), anti-CD14-PE-Fire640 (63D3) (all BioLegend), and Live Dead Blue (BD Biosciences). PBMCs were incubated with antibodies using Brilliant Stain Buffer and 5% FSB for 20min at room temperature. Cells were washed twice with PBS, centrifuged, and passed through a 70 μm filter. Cells were sorted on Cytek Aurora Spectrum Cytometry (Cytek Biosciences B.V., Amsterdam, The Netherlands). Intact cells were gated according to FSC-A and SSC-A. Doublets were excluded by serial FSC-H/FSC-W and SSC-H/SSC-W gates. Non-viable cells were excluded based on Live Dead Blue staining. Cells were sorted through a 100-micron nozzle at 20 psi into eppendorf containing RNA later and they were immediately frozen and stored at −80 °C. Flow cytometric quantification of cell populations was performed using FlowJo 10.10.

1.2 RNA and miRNA Extraction

Immune cell subpopulations were isolated using Cytek Aurora Spectrum Cytometry, as described previously. RNA and miRNA were extracted using the miRNeasy Kit (Qiagen, Basel, Switzerland), which is specifically designed for the efficient isolation of both total RNA and miRNA. This method ensures optimized recovery of miRNA for downstream analysis.

Initially, 700 µL of QIAzol was used to lyse the cells, followed by the addition of 140 µL of chloroform. The samples were then centrifuged at 4ºC for 15 minutes at 12,000 × g to separate the aqueous phase. Both RNA and miRNA were purified using a mini column, with washing steps and elution volumes optimized to achieve high concentrations and quality.

To further optimize miRNA extraction, the RNeasy MinElute Cleanup Kit (Qiagen) was employed, allowing the separation and purification of an miRNA-enriched fraction alongside a total RNA (>200 nt) fraction. This ensures that both miRNA and RNA of appropriate size are available for downstream analyses.

The integrity and quantity of RNA and miRNA are crucial for accurate results in downstream experiments. To assess this, samples were evaluated using the Bioanalyzer PicoChip system (ThermoFisher), which employs capillary electrophoresis to separate RNA molecules by size. The system generates an electropherogram and RNA Integrity Number (RIN), with only samples showing a minimum RIN of 8 being considered acceptable for the study (Table S2). Additionally, the Agilent 2100 Bioanalyzer was used to confirm the presence of small RNA bands, detected within the size range of 25–200 base pairs, confirming miRNA extraction quality.

1.3 Microarray Analysis

For mRNA expression profiling, Affymetrix Human Transcriptome Array 2.0 (HTA 2.0) (Thermo Fisher Scientific, Inc.) was used. For miRNA expression profiling, Affymetrix GeneChip miRNA 4.0

Array (Thermo Fisher Scientific, Inc.) was used. This microarray contains 30,424 total mature miRNA probe sets, including 2,578 mature human miRNAs and miRNAs from 202 other organisms. After hybridization and washing, the arrays were scanned by an Affymetrix Microarray Scanner (Applied Biosystems, Grand Island, NY, USA). Raw data of HTA 2.0 were extracted and normalized by Affymetrix® Transcriptome Analysis Console (TAC) Software (Thermo Fisher Scientific, Inc.). miRNA QC Tool software (Thermo Fisher Scientific, Inc.) was used for miRNA 4.0 array data summarization, normalization, and quality control. Resultant array data will be deposited in NCBI's Gene Expression Omnibus (https://www.ncbi.nlm.nih.gov/geo) per GEO guidelines.

The identification of differentially expressed genes and miRNAs between pre- and post-treatment samples was performed using the statistical language R (version 4.3.0, Copyright © 2018 The R Foundation for Statistical Computing). The analysis utilized libraries developed for microarray analysis within the Bioconductor Project [16]. To identify differentially expressed genes, we applied a linear model with empirical Bayes moderation of the variance, a method specifically developed for microarray data analysis by Gordon K. Smyth [17]. To account for patient-specific effects, a fixed effect was included in the linear model.

Quality control analyses were conducted before and after array normalization, including principal component analysis (PCA), heatmaps depicting the distances between arrays, and hierarchical clustering. Additionally, quality assessment was performed using the Array Quality Metrics Bioconductor package [1]. All samples passed quality control. Array normalization was carried out using the RMA method [2], identifying 6,631 small RNA sequences and RNA genes. From the filtered miRNA analysis, we detected 2,578 mature miRNA sequences, 1,491 snoRNAs from the Ensembl database [3], 155 H/ACA box, 319 C/D box, and 31 scaRNA classes from the snoRNABase [4]. The array also included 10 probe sets targeting 5.8s rRNA, which were used as a reference. Only sequences corresponding to mature miRNAs were included in the differential expression analysis.
The biological significance of mRNAs was analysed using gene set enrichment analysis (GSEA) based on the Gene Ontology (GO) and Reactome Pathway Knowledge Base [22], with the reference set consisting of all genes analysed in the study. GO terms and Reactome pathways with an adjusted p-value below 0.05 were considered significant. For miRNAs, biological significance was assessed using enrichment analysis on GO and Reactome Pathway Knowledge Base to identify key pathways associated with the target genes of the selected miRNAs. Only validated miRNA-target interactions with strong experimental evidence (validated by luciferase assay or western blot) were considered. The analysis was performed using the clusterProfiler package v4.4.1 from R/Bioconductor, with GO and Reactome terms selected based on an adjusted p-value below 0.05.

We carefully considered the potential for batch effects in our study. To minimize this risk, we randomly mixed samples from different time points and patient groups before conducting microarray experiments. Additionally, we employed robust bioinformatics approaches to detect and correct any potential batch effects during data analysis. Principal component analysis (PCA) was performed both before and after data normalization to assess the influence of various factors, including patient, time point, cell type, and batch. No major batch effects or biases were identified, except for the patient factor, which was accounted for as a fixed effect in the linear model, given the use of paired samples. This rigorous approach ensured that observed differences in gene expression were driven by biological variation rather than technical artifacts.

To control the false discovery rate (FDR) and reduce the likelihood of false positives, we applied multiple testing correction using the Benjamini-Hochberg method in our differential expression analysis. Given the large number of miRNAs and mRNAs analysed, proper adjustment for multiple

comparisons was crucial. The Benjamini-Hochberg correction allowed us to maintain statistical rigor by controlling the expected proportion of false discoveries among significant results. This method is particularly valuable in high-dimensional transcriptomic analyses, where numerous comparisons increase the risk of Type I errors. By implementing this correction, we ensured that our findings remained statistically valid and biologically meaningful

2 References

[1] Gentleman RA, Carey VJ, Huber W, Irizarry RA, Dudoit S. Bioinformatics and Computational Biology Solutions using R and Bioconductor. Springer, New York, (2005).

[2] Takano H, Miyashita S. Relaxation modes in random spin systems. J Phys Soc Jpn. 1995;64:3688–3698. doi: 10.1143/JPSJ.64.3688.

[3] Yates A, Akanni W, Amode MR et al. Ensembl 2016. Nucleic Acids Res. (2016) 44(D1): D710-6. doi: 10.1093/nar/gkv1157.

[4] Xie J, Zhang M, Zhou T et al. Sno/scaRNAbase: a curated database for small nucleolar RNAs and cajal body-specific RNAs. Nucleic Acids. Res (2007) 35:D183-7. doi: 10.1093/nar/gkl873.

3 Supplementary Figures

Figure S1. Gating strategy used to analyse immune cell subsets in isolated PBMCs. The gating process was applied to identify, differentiate and quantify various immune cell populations based on specific surface markers.

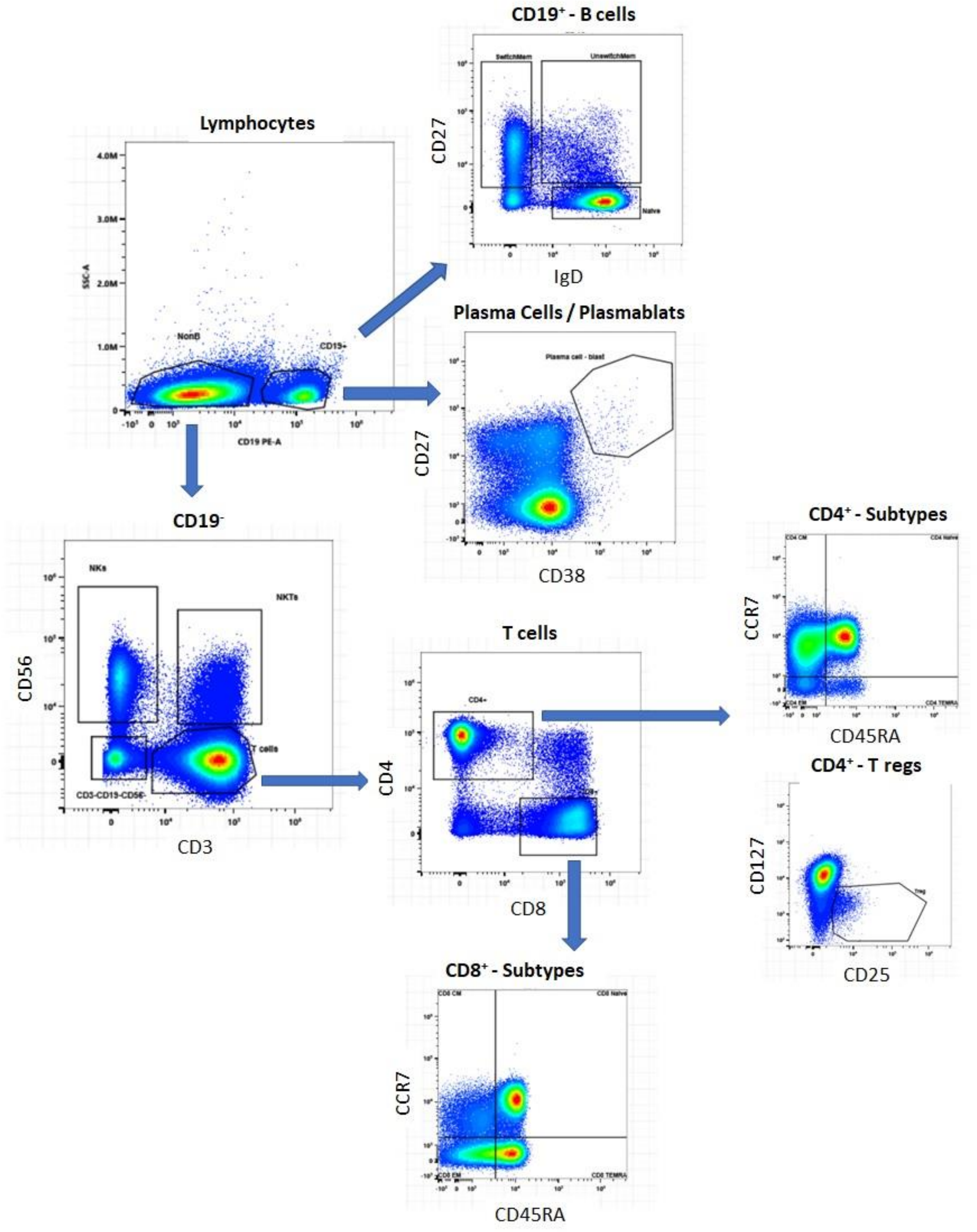

Figure S2. Median change from baseline to six months of belimumab treatment in B cell subsets from SLE patients. The frequencies of naïve B cells, switched and non-switched B cells, and double-negative (DN) B cells were analyzed. Statistical comparisons were performed using one-way ANOVA followed by Student's t-test. No significant differences were observed.

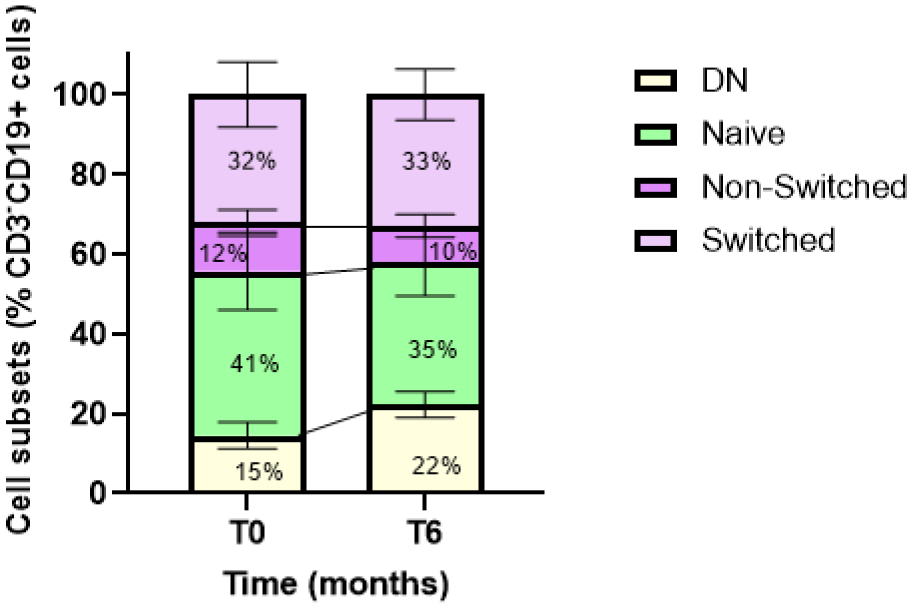

Figure S3. Correlation analysis between clinical data and miRNA expression in pre- and post-treatment samples. No significant correlations were observed between miRNA expression levels and clinical parameters, including anti-dsDNA titers, complement values, SLEDAI, or PGA scores. Each graph includes the correlation coefficient (r) and the corresponding p-value for each analysis.

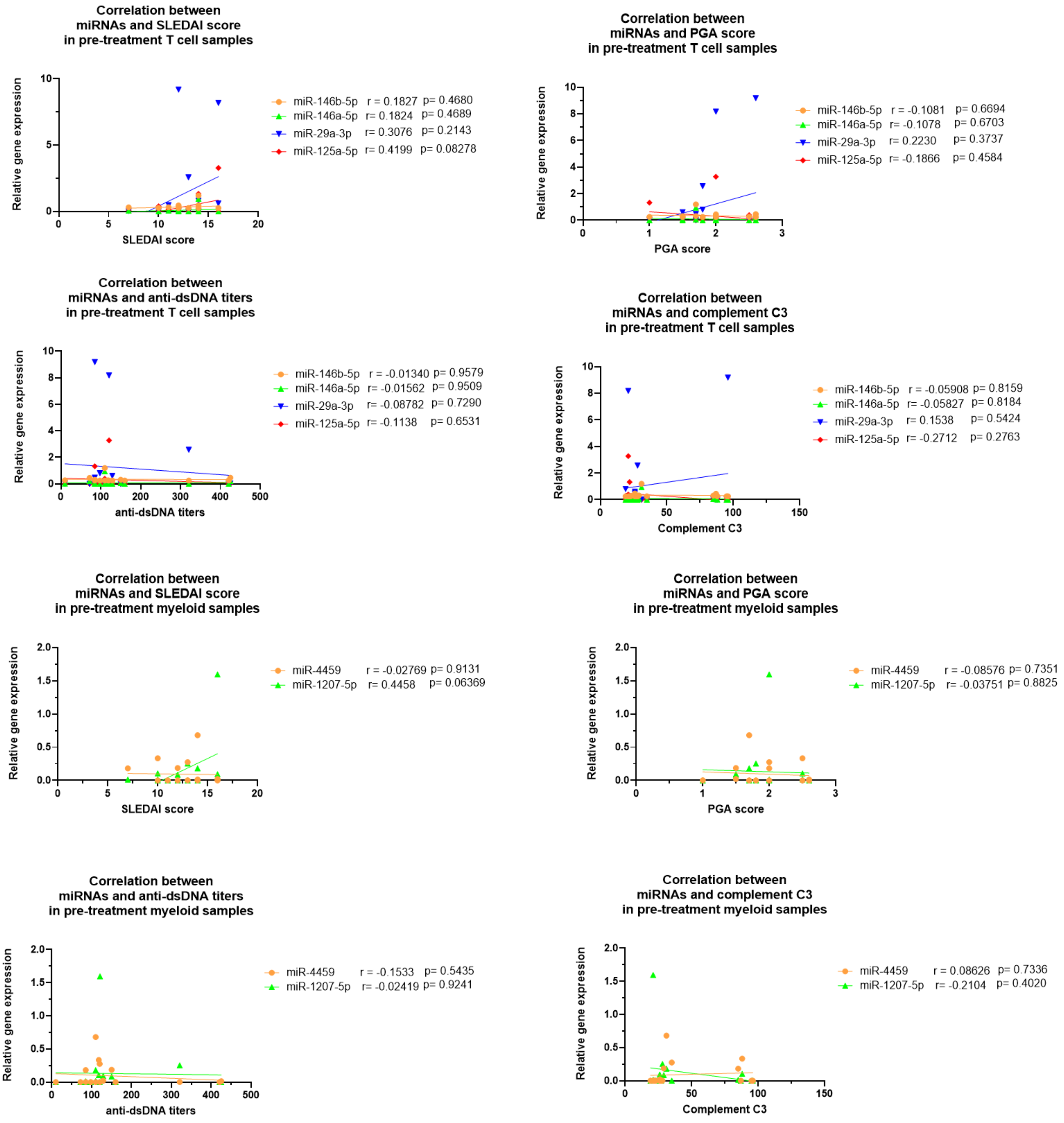

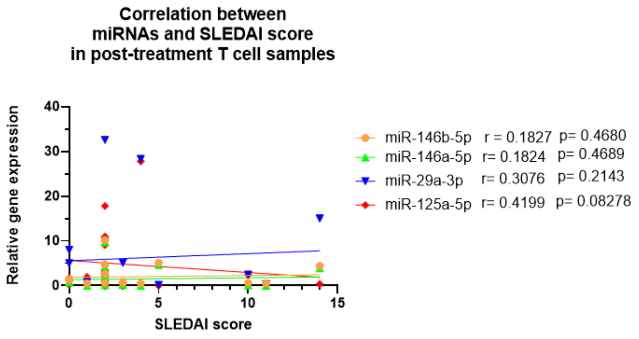
Correlation between
miRNAs and SLEDAI score
in post-treatment T cell samples
Relative gene expression
SLEDAI score
miR-146b-5p r = 0.1827 p= 0.4680
miR-146a-5p r= 0.1824 p= 0.4689
miR-29a-3p r= 0.3076 p= 0.2143
miR-125a-5p r= 0.4199 p= 0.08278

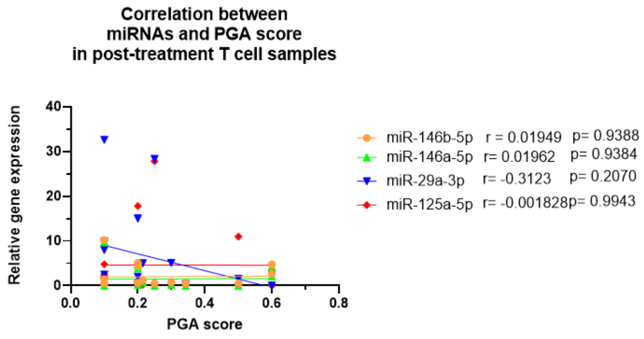
Correlation between
miRNAs and PGA score
in post-treatment T cell samples
Relative gene expression
PGA score
miR-146b-5p r = 0.01949 p= 0.9388
miR-146a-5p r= 0.01962 p= 0.9384
miR-29a-3p r= -0.3123 p= 0.2070
miR-125a-5p r= -0.001828p= 0.9943

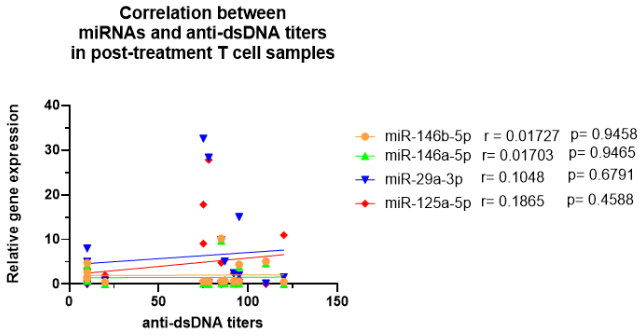
Correlation between
miRNAs and anti-dsDNA titers
in post-treatment T cell samples
Relative gene expression
anti-dsDNA titers
miR-146b-5p r = 0.01727 p= 0.9458
miR-146a-5p r= 0.01703 p= 0.9465
miR-29a-3p r= 0.1048 p= 0.6791
miR-125a-5p r= 0.1865 p= 0.4588

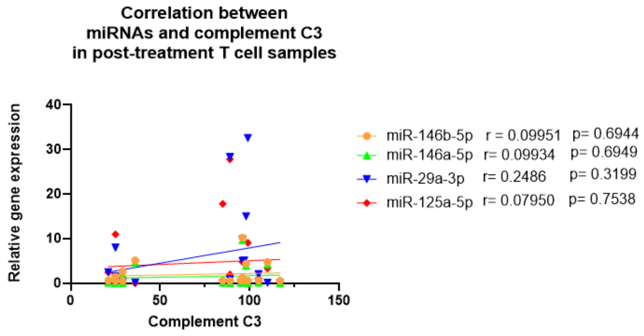
Correlation between
miRNAs and complement C3
in post-treatment T cell samples
Relative gene expression
Complement C3
miR-146b-5p r = 0.09951 p= 0.6944
miR-146a-5p r= 0.09934 p= 0.6949
miR-29a-3p r= 0.2486 p= 0.3199
miR-125a-5p r= 0.07950 p= 0.7538

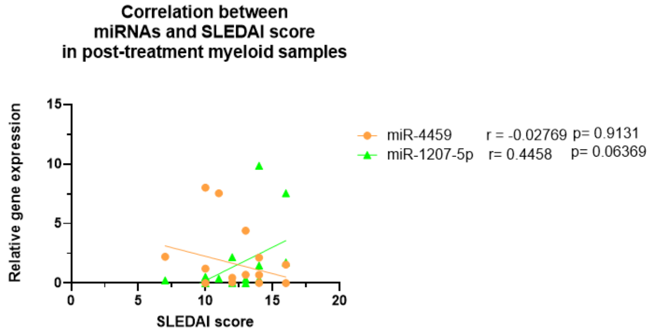
Correlation between
miRNAs and SLEDAI score
in post-treatment myeloid samples
Relative gene expression
SLEDAI score
miR-4459 r = -0.02769 p= 0.9131
miR-1207-5p r= 0.4458 p= 0.06369

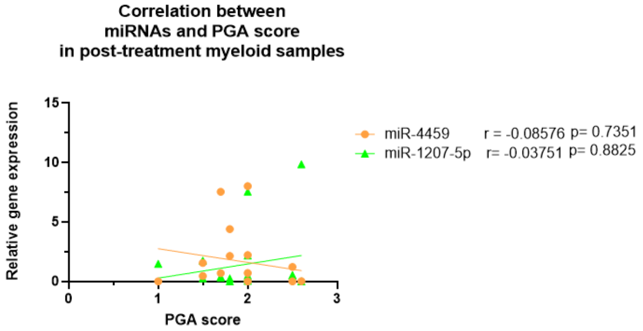
Correlation between
miRNAs and PGA score
in post-treatment myeloid samples
Relative gene expression
PGA score
miR-4459 r = -0.08576 p= 0.7351
miR-1207-5p r= -0.03751 p= 0.8825

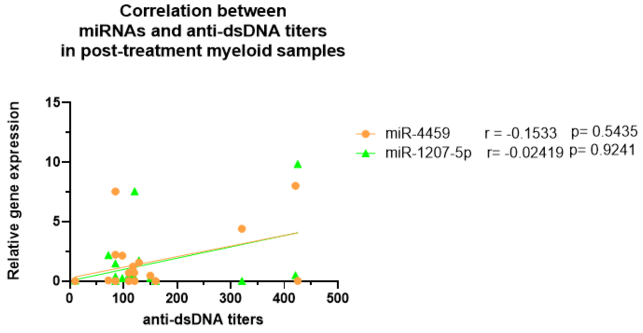
Correlation between
miRNAs and anti-dsDNA titers
in post-treatment myeloid samples
Relative gene expression
anti-dsDNA titers
miR-4459 r = -0.1533 p= 0.5435
miR-1207-5p r= -0.02419 p= 0.9241

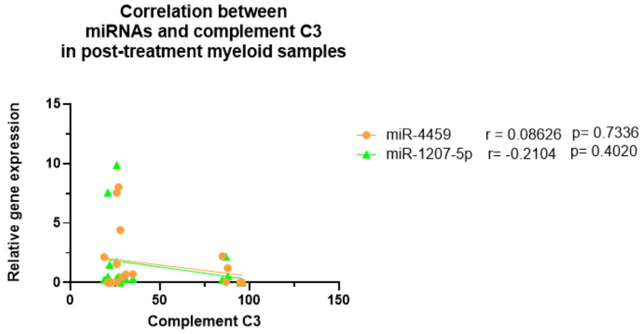
Correlation between
miRNAs and complement C3
in post-treatment myeloid samples
Relative gene expression
Complement C3
miR-4459 r = 0.08626 p= 0.7336
miR-1207-5p r= -0.2104 p= 0.4020

Figure S4. miRNA expression levels in T and myeloid samples from a cohort of SLE patients not treated with belimumab. No significant differences were observed between pre- and post-treatment samples. P-values are shown in each graph.

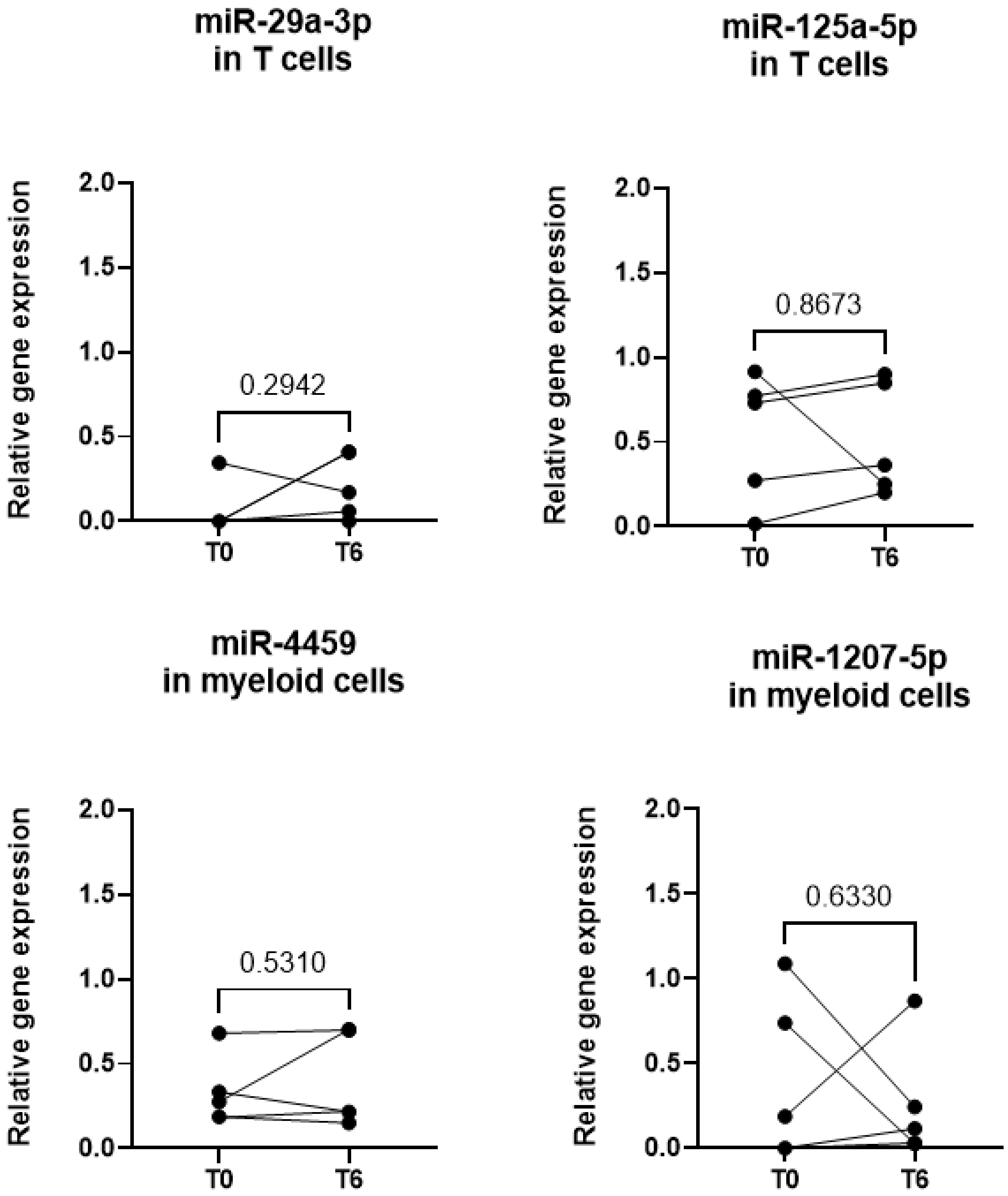

4 Supplementary Tables

Supplementary Table 1. Flow cytometry antibodies for PBMCs characterization.

Antibody	Fluorophore	Supplier
**CD3**	PE	BD Biosciences
**CD4**	FITC	BD Biosciences
**CD8**	APC	BD Biosciences
**CD19**	PECy7	BD Biosciences
**CD56**	BB700	BD Biosciences
**CD11c**	BV650	BD Biosciences
**CD68**	PECF594	BD Biosciences
**CCR7**	BV421	BD Biosciences
**CD45RA**	PerCPCy55	BioLegend
**IgD**	R718	BD Biosciences
**CD27**	BV786	BD Biosciences
**CD138**	A647	BD Biosciences
**CD38**	APC-Fire 810	BioLegend
**CD127**	BV711	BioLegend
**CD25**	BV510	BioLegend
**CD16**	Pacific Blue	BioLegend
**CD14**	PE	BioLegend

Supplementary Table 2. RNA Integrity Number (RIN) values for all the samples used in the microarray analysis.

Sample time	Patient	T cells	B cells	Myeloid cells
Pre-treatment	1	8.3	8.6	8.1
Post-treatment		8.2	8.0	8.8
Pre-treatment	**2**	8.8	8.6	8.0
Post-treatment		8.7	8.3	8.6
Pre-treatment	**3**	8.6	8.1	8.0
Post-treatment		8.0	8.7	8.3
Pre-treatment	**4**	8.3	8.1	8.7
Post-treatment		8.7	8.8	8.8
Pre-treatment	**5**	8.6	8.2	8.4
Post-treatment		8.2	8.3	8.4
Pre-treatment	**6**	8.4	8.6	8.1
Post-treatment		8.4	8.4	8.3
Pre-treatment	**7**	8.1	8.9	8.9
Post-treatment		8.7	8.7	8.2
Pre-treatment	**8**	8.8	8.4	8.6
Post-treatment		8.2	8.6	8.4
Pre-treatment	**9**	8.8	8.1	8.9
Post-treatment		8.9	8.1	8.3
Pre-treatment	**10**	8.8	8.2	8.7
Post-treatment		8.7	8.2	8.0

Supplementary Table 3. Primer IDs used in Taqman RT-qPCR from Applied Biosystems.

miRNAs	Assay ID (TaqMan)
hsa-miR-8069	480611_mir
hsa-miR-125a-5p	477884_mir
hsa-miR-146b-5p	483144_mir
hsa-miR-146a-5p	478399_mir
hsa-miR-29a-3p	478587_mir
hsa-miR-1207-5p	477873_mir
hsa-miR-4459	480823_mir

Gene	Assay ID (TaqMan)
**GADPH**	Hs02786624_g1
**IRAK1**	Hs00155570_m1
**IRF5**	Hs00158114_m1
**NFKB1**	Hs00765730_m1
**TRAF6**	Hs00939742_g1
**EGFR**	Hs01076090_m1
**PIK3B**	Hs00898499_m1
**AKT3**	Hs00987343_m1
**STAT1**	Hs01013996_m1
**IFNA**	Hs03044218_g1
**IFNB**	Hs01077958_s1
**KLF13**	Hs00429818_m1
**CCL5**	Hs00982282_m1
**CXCL10**	Hs00171042_m1
**IL4**	Hs00174122_m1
**TNFA**	Hs00174128_m1
**IFNG**	Hs00989291_m1
**BLNK**	Hs00179459_m1
**BANK1**	Hs01009378_m1
**MEF2C**	Hs00231149_m1
**CD40**	Hs01002915_g1
**MAPK1**	Hs01046830_m1
**JAK1**	Hs01026983_m1
**AKT2**	Hs01086099_m1
**JUN**	Hs01103582_s1
**STAT3**	Hs00374280_m1
**STAT5**	Hs00559637_g1

Supplementary Table 4. List of differentially expressed genes in B cells between post-treatment and pre-treatment with belimumab.

Affymetrix ID	Gene Symbol	Entrez	logFC	AveExpr	t	P.Value	adj.P.Val	B
TC1600007312.hg.1	IL4R	3566	-1,21759567	9,38850392	-5,35432441	2,34E-06	0,03786196	4,35476158
TC1400010704.hg.1	CRIP1	1396	0,7613047	9,50843003	5,19272274	4,10E-06	0,03786196	3,88176368
TC1100013140.hg.1	TAF10	6881	0,39749906	9,80788132	5,03230654	7,11E-06	0,04378379	3,41488451
TC0100016917.hg.1	CSRP1	1465	0,66874703	8,96248741	4,86577561	1,25E-05	0,05790257	2,93362245
TC1600011570.hg.1	CMC2	56942	1,01527246	7,5115922	4,6757686	2,38E-05	0,07535926	2,38962595
TC1100006477.hg.1	PHRF1	57661	-0,48394531	7,96650302	-4,66731037	2,45E-05	0,07535926	2,36555206
TC1900009991.hg.1	JAK3	3718	-0,50954022	8,43962113	-4,59849192	3,08E-05	0,08129834	2,17016859
TC1800009289.hg.1	ACAA2	10449	1,11711084	6,91865068	4,49364501	4,36E-05	0,09952561	1,8742579
TC1900007950.hg.1	ZNF567	163081	-1,09626846	6,2222115	-4,42134109	5,54E-05	0,09952561	1,67151968
TC0600010867.hg.1	MCUR1	63933	1,33829276	6,72900112	4,39038149	6,13E-05	0,09952561	1,58505939
TC2200007035.hg.1	UQCR10	29796	0,71603768	9,66777767	4,38673877	6,21E-05	0,09952561	1,5749006
TC0800007102.hg.1	PTK2B	2185	-0,50757412	9,1627659	-4,33256703	7,41E-05	0,09952561	1,42418686
TC1900010696.hg.1	AKT2	208	-0,78657643	7,92810572	-4,32082242	7,70E-05	0,09952561	1,39160214
TC1200007832.hg.1	NABP2	79035	0,61533095	7,54649682	4,281912	8,74E-05	0,09952561	1,28388405
TC0100018389.hg.1	CDK11A	728642	-0,54586871	9,69877367	-4,28133567	8,76E-05	0,09952561	1,28229133
TC1100006911.hg.1	SPON1	10418	-0,43206031	5,1448857	-4,27834546	8,85E-05	0,09952561	1,274029
TC0600014294.hg.1	ZNF318	24149	-0,91281975	7,30630174	-4,26376054	9,28E-05	0,09952561	1,23376061
TC0500008767.hg.1	PKD2L2	27039	-0,2849879	3,81485704	-4,2396558	0,0001003	0,09952561	1,16732449
TC0200013594.hg.1	COA5	493753	0,86395621	8,56791606	4,23342685	0,00010235	0,09952561	1,15018037
TC0100008938.hg.1	LMO4	8543	0,75689966	6,70408586	4,1959089	0,00011556	0,10238044	1,04712847
TC0100015817.hg.1	THEM4	117145	1,08522569	6,74179435	4,18613116	0,00011926	0,10238044	1,02033145
TC1500009455.hg.1	MYO5C	55930	-0,32532715	6,47510244	-4,17933261	0,00012191	0,10238044	1,00171401
TC1200009131.hg.1	DYNLL1	8655	0,74132728	8,07875431	4,15646104	0,00013123	0,1051929	0,93917125
TC1700011424.hg.1	SMARCD2	6603	-0,54272781	8,73806262	-4,13904361	0,00013879	0,1051929	0,89163673
TC0300007495.hg.1	SPCS1	28972	0,54114044	7,85854073	4,11949024	0,00014778	0,1051929	0,83837086
TC1700009713.hg.1	STX8	9482	0,46641919	7,41749177	4,11896349	0,00014803	0,1051929	0,83693738
TC0200011980.hg.1	ITSN2	50618	-0,65478285	10,0350522	-4,08146687	0,00016691	0,11112569	0,7350919
TC1200010109.hg.1	LDHB	3945	0,92828028	8,30879817	4,07867033	0,00016841	0,11112569	0,72751186
TC0300014050.hg.1	NPHP3	27031	0,72699555	6,15374316	4,0500119	0,00018453	0,1175657	0,64996093
TC0100015819.hg.1	S100A10	6281	1,06383512	8,76990434	4,01588767	0,00020569	0,124243	0,55792723
TC0100009938.hg.1	MLLT11	10962	0,61462354	5,46352783	4,00374354	0,00021377	0,124243	0,52525634
TC1500006756.hg.1	KLF13	51621	-0,37355768	5,53113828	-3,99678026	0,00021854	0,124243	0,50654294

TC0200010112.hg.1	RBM45	129831	1,05852496	7,05518894	3,99194364	0,00022191	0,124243	0,49355329
TC2100008559.hg.1	CFAP298	56683	0,65198432	8,97137349	3,97306171	0,00023557	0,12522333	0,44290921
TC0300011485.hg.1	FOXP1	27086	-0,59328032	10,55457	-3,96072723	0,00024493	0,12522333	0,40988431
TC0X00008828.hg.1	EMD	2010	0,77847355	7,16841877	3,95115248	0,00025245	0,12522333	0,38428028
TC1900006683.hg.1	YJU2	55702	-0,38284346	8,22417578	-3,93875314	0,00026251	0,12522333	0,3511646
TC1100008805.hg.1	C11orf97	643037	-0,25210077	4,16971726	-3,93277246	0,0002675	0,12522333	0,33520856
TC0100016658.hg.1	ARPC5	10092	0,61018884	10,041914	3,92987817	0,00026994	0,12522333	0,32749075
TC0600014267.hg.1	ATF6B	1388	-0,39485955	10,395853	-3,92026002	0,00027824	0,12522333	0,30186198
TC1000011005.hg.1	P4HA1	5033	0,57373086	4,70421254	3,91349877	0,00028421	0,12522333	0,28386304
TC0800007013.hg.1	CHMP7	91782	-0,54891605	7,58302836	-3,90924354	0,00028803	0,12522333	0,27254262
TC1000011661.hg.1	KCNIP2	30819	-0,68018954	6,18095248	-3,90386273	0,00029294	0,12522333	0,25823589
TC0200014697.hg.1	ACVR1	90	0,775364	7,30592734	3,87173089	0,00032396	0,12522333	0,17299299
TC0300007479.hg.1	PHF7	51533	-0,49344443	5,25931729	-3,87063218	0,00032507	0,12522333	0,17008403
TC1100007800.hg.1	TKFC	26007	-0,39494198	7,81984453	-3,8689292	0,00032681	0,12522333	0,16557596
TC1700012277.hg.1	CDK5RAP3	80279	-0,75383467	7,73045133	-3,86726002	0,00032852	0,12522333	0,16115828
TC0100007886.hg.1	NDUFS5	4725	0,61540807	10,0276307	3,86328937	0,00033262	0,12522333	0,15065306
TC1700006911.hg.1	ARHGAP44	9912	-0,53519366	4,49906847	-3,8623273	0,00033362	0,12522333	0,14810845
TC1700011435.hg.1	ICAM2	3384	0,62083923	7,64325483	3,85731804	0,00033888	0,12522333	0,13486418
TC0800012435.hg.1	ZFAND1	79752	0,84841419	7,07749324	3,84307765	0,00035428	0,12815835	0,09725758
TC0300007359.hg.1	NDUFAF3	25915	0,45115166	8,80943068	3,83731213	0,0003607	0,12815835	0,08205056
TC0200016584.hg.1	HSPE1	3336	0,68852349	10,2511581	3,82899302	0,00037016	0,12849899	0,06012739
TC0X00007716.hg.1	P2RY10	27334	-0,72705076	6,70169402	-3,81917392	0,00038164	0,12849899	0,03428066
TC0800012308.hg.1	ADHFE1	137872	1,01193296	7,80589862	3,81611311	0,00038529	0,12849899	0,0262302
TC2200007904.hg.1	ATP6V1E1	529	0,49964747	7,30735342	3,81100111	0,00039146	0,12849899	0,01279173
TC0300013051.hg.1	PDCD10	11235	0,86612219	9,07273479	3,80693523	0,00039643	0,12849899	0,00210952
TC1900009636.hg.1	KRI1	65095	-0,38927056	5,55480234	-3,79094426	0,00041659	0,13004217	-0,03984977
TC1000007698.hg.1	CISD1	55847	0,87642228	6,91683647	3,78896479	0,00041915	0,13004217	-0,04503783
TC0900010019.hg.1	ZBTB5	9925	-0,41890675	4,95577127	-3,78285955	0,00042715	0,13004217	-0,06103088
TC1000007272.hg.1	CREM	1390	0,9825923	6,47030724	3,779306	0,00043187	0,13004217	-0,07033383
TC0400008137.hg.1	CCSER1	401145	-1,22415767	4,76269729	-3,77594833	0,00043638	0,13004217	-0,07912004
TC0600008272.hg.1	FBXO9	26268	0,5299667	8,21661651	3,76090476	0,00045715	0,13406842	-0,11843843
TC0400010965.hg.1	GRSF1	2926	0,6404646	7,75686601	3,74168302	0,00048507	0,13487022	-0,16856425
TC2200009278.hg.1	RBX1	9978	0,74246391	9,84897339	3,71948392	0,00051936	0,13487022	-0,22629525
TC1700010716.hg.1	GHDC	84514	-0,36513955	6,94365378	-3,71682994	0,00052361	0,13487022	-0,23318568
TC1900009310.hg.1	MATK	4145	-0,32776073	7,79303093	-3,71667688	0,00052386	0,13487022	-0,23358299
TC0400009919.hg.1	MRFAP1L1	114932	0,38315801	8,96747029	3,71507433	0,00052644	0,13487022	-0,23774235

TC2000007191.hg.1	DYNLRB1	83658	0,61722434	8,74209129	3,71394001	0,00052828	0,13487022	-0,2406859
TC0X00009100.hg.1	TRAPPC2	6399	0,89340856	7,35500658	3,7133494	0,00052924	0,13487022	-0,24221834
TC0100015598.hg.1	TXNIP	10628	-0,49345962	11,6780021	-3,70967249	0,00053525	0,13487022	-0,25175599
TC1400006718.hg.1	PSME1	5720	0,39988331	9,86597437	3,7085582	0,00053708	0,13487022	-0,25464545
TC2200007001.hg.1	HSCB	150274	0,4618698	6,77607662	3,70613874	0,00054108	0,13487022	-0,26091779
TC1100011549.hg.1	PHOX2A	401	-0,31622459	4,53480068	-3,70339771	0,00054565	0,13487022	-0,26802133
TC1100013100.hg.1	ATP5MG	10632	0,37138857	9,96937354	3,6999108	0,00055151	0,13487022	-0,27705402
TC1500010878.hg.1	INTS14	81556	0,82614389	6,84116696	3,69445884	0,0005608	0,13487022	-0,29116844
TC0200011426.hg.1	NEU4	129807	-0,42084814	6,60509736	-3,69371603	0,00056208	0,13487022	-0,29309065
TC1200009172.hg.1	ORAI1	84876	-0,55652247	6,17176194	-3,68022525	0,00058577	0,13807025	-0,32796752
TC1900011004.hg.1	SLC1A5	6510	0,59640712	7,56983765	3,67767258	0,00059036	0,13807025	-0,33455947
TC0300011525.hg.1	RYBP	23429	0,6802559	8,10454891	3,66873571	0,00060671	0,14011844	-0,35761938
TC1000012011.hg.1	RGS10	6001	1,25631441	10,1715026	3,65576227	0,0006312	0,14199567	-0,39104362
TC1500008023.hg.1	ZFAND6	54469	0,66877602	9,23120645	3,64932822	0,0006437	0,14199567	-0,40759746
TC0900011378.hg.1	CDK5RAP2	55755	0,74870316	8,36208646	3,64494811	0,00065234	0,14199567	-0,41885818
TC1600010227.hg.1	N4BP1	9683	1,06485059	9,52772968	3,643353	0,00065552	0,14199567	-0,42295726
TC0200011130.hg.1	DGKD	8527	-0,59331996	8,39942197	-3,64083838	0,00066055	0,14199567	-0,42941742
TC0400012638.hg.1	UFSP2	55325	0,60291327	5,54764269	3,64064394	0,00066095	0,14199567	-0,42991685
TC0100016548.hg.1	ABL2	27	-0,59721031	6,38642435	-3,63442608	0,00067356	0,14304341	-0,44588039
TC1800007667.hg.1	TIMM21	29090	0,84551578	6,75051691	3,62900741	0,00068475	0,14357203	-0,45978052
TC0900010958.hg.1	TRMO	51531	-0,53096818	9,45554894	-3,62573274	0,0006916	0,14357203	-0,46817558
TC0300014034.hg.1	COX17	10063	0,89253684	8,7674166	3,61949628	0,00070481	0,14469045	-0,48415264
TC1400008110.hg.1	C14orf132	56967	-0,28076082	4,76819733	-3,61162354	0,00072185	0,14524648	-0,50430107
TC1200009457.hg.1	ULK1	8408	-0,37324335	7,8443349	-3,60466383	0,00073723	0,14524648	-0,52209366
TC0100012441.hg.1	RNF223	401934	-0,35993737	7,84721195	-3,59398584	0,00076145	0,14524648	-0,54935692
TC0200008094.hg.1	SEMA4F	10505	-0,45413724	6,32175342	-3,59133272	0,00076758	0,14524648	-0,55612431
TC1600008021.hg.1	MMP15	4324	-0,39453659	4,48506291	-3,59130132	0,00076766	0,14524648	-0,55620438
TC0100013574.hg.1	PEF1	553115	-0,43707691	5,49268481	-3,58674003	0,00077831	0,14524648	-0,56783274
TC0100018246.hg.1	LRRC8C	84230	0,59987488	7,88157596	3,58425577	0,00078418	0,14524648	-0,57416273
TC1900007053.hg.1	ZNF69	7620	-0,57151349	5,99786689	-3,58184261	0,00078991	0,14524648	-0,58030929
TC2200007495.hg.1	SREBF2	6721	-0,51209064	8,46538704	-3,57905465	0,00079659	0,14524648	-0,58740781
TC0900011970.hg.1	QSOX2	169714	-0,74460225	5,86111261	-3,57793907	0,00079928	0,14524648	-0,59024739
TC1100010895.hg.1	TIMM10	26519	0,60129909	8,99514818	3,57766957	0,00079993	0,14524648	-0,59093329
TC2200008859.hg.1	NDUFA6	4700	0,69350688	9,30081217	3,57604129	0,00080387	0,14524648	-0,5950769
TC0600009140.hg.1	GTF3C6	112495	0,80010279	7,47936714	3,57363781	0,00080972	0,14524648	-0,60119135
TC1200007989.hg.1	RXYLT1	10329	0,61391456	6,25730707	3,5657525	0,0008292	0,14607396	-0,62123622

TC0400007280.hg.1	WDR19	57728	-0,69436682	5,43957832	-3,56537524	0,00083015	0,14607396	-0,62219464
TC1500010726.hg.1	TMEM62	80021	-0,80516488	5,41367974	-3,5575208	0,00085002	0,14815973	-0,64213638
TC0600010060.hg.1	MRPL18	29074	0,50950245	7,86140157	3,54634483	0,00087908	0,14858773	-0,67047049
TC1500007614.hg.1	MAP2K1	5604	0,34660853	8,3648587	3,54514763	0,00088225	0,14858773	-0,67350287
TC1300008181.hg.1	CHAMP1	283489	0,56006757	6,64290186	3,54203059	0,00089055	0,14858773	-0,68139543
TC0X00008866.hg.1	VBP1	7411	0,58365656	5,77824345	3,54138803	0,00089227	0,14858773	-0,68302195
TC1100007457.hg.1	ATG13	9776	-0,42461865	7,80492532	-3,53667058	0,000905	0,14858773	-0,69495855
TC0200016707.hg.1	ZNF514	84874	0,45034986	6,351276	3,53428432	0,00091151	0,14858773	-0,70099324
TC0300006483.hg.1	BHLHE40	8553	0,84328981	8,09163175	3,53115244	0,00092011	0,14858773	-0,70891022
TC0900009685.hg.1	HACD4	401494	1,35624111	9,01817169	3,52715488	0,00093121	0,14858773	-0,71900998
TC0200010838.hg.1	STK16	8576	-0,48035187	5,1845005	-3,52253594	0,00094419	0,14858773	-0,73067194
TC1700007016.hg.1	TRPV2	51393	0,36518649	10,0628841	3,51866138	0,00095521	0,14858773	-0,74044806
TC1100012315.hg.1	TIMM8B	26521	0,5490593	9,10626477	3,51739779	0,00095883	0,14858773	-0,74363501
TC0100009307.hg.1	NTNG1	22854	-0,58439718	5,12717159	-3,51232992	0,00097349	0,14858773	-0,75641069
TC1900009491.hg.1	FCER2	2208	-1,07503115	6,13001663	-3,50178995	0,00100466	0,14858773	-0,78294885
TC0100013710.hg.1	TRAPPC3	27095	0,76181467	7,83720427	3,50058262	0,00100829	0,14858773	-0,78598595
TC0300014032.hg.1	POPDC2	64091	0,62462236	7,2472097	3,49745768	0,00101774	0,14858773	-0,79384422
TC2000007473.hg.1	YWHAB	7529	0,31749203	10,8957086	3,49624737	0,00102143	0,14858773	-0,79688676
TC0700009485.hg.1	GSTK1	373156	0,4393778	8,06807869	3,49393261	0,00102851	0,14858773	-0,8027041
TC0100018261.hg.1	GSTM4	2948	0,68867063	6,06057896	3,49141319	0,00103627	0,14858773	-0,80903336
TC1900011745.hg.1	ARHGEF1	9138	-0,78779371	9,1449089	-3,49110106	0,00103724	0,14858773	-0,80981732
TC2200006678.hg.1	SNAP29	9342	0,68629143	8,12756672	3,48353263	0,00106091	0,14858773	-0,82881459
TC0400009694.hg.1	ATP5ME	521	0,39582461	10,4549375	3,47986313	0,00107257	0,14858773	-0,83801711
TC0600011927.hg.1	SUPT3H	8464	0,88350091	7,27796204	3,4790341	0,00107523	0,14858773	-0,84009545
TC0200011690.hg.1	YWHAQ	10971	0,37523572	10,0972751	3,47902869	0,00107524	0,14858773	-0,84010901
TC2000008379.hg.1	MKKS	8195	1,01472873	6,34942935	3,4780738	0,00107831	0,14858773	-0,84250251
TC1100008049.hg.1	BANF1	8815	0,47396233	9,30714194	3,47748273	0,00108021	0,14858773	-0,8439839
TC0600013206.hg.1	SLC18B1	116843	-0,4144466	6,60544697	-3,47713479	0,00108133	0,14858773	-0,84485586
TC1600006941.hg.1	SNX29	92017	-0,5052109	8,35983542	-3,47638248	0,00108375	0,14858773	-0,84674107
TC0X00009750.hg.1	KDM5C	8242	-0,51188515	8,76634526	-3,47612197	0,00108459	0,14858773	-0,84739381
TC0500009183.hg.1	MRPL22	29093	0,53331349	6,9942183	3,47577962	0,0010857	0,14858773	-0,84825159
TC0600007302.hg.1	BTN2A2	10385	-0,63422227	8,1955564	-3,46928396	0,00110689	0,14907163	-0,86451794
TC1900010632.hg.1	SIRT2	22933	0,36290017	7,91684572	3,46802859	0,00111103	0,14907163	-0,86765966
TC1600007143.hg.1	LYRM1	57149	0,89911223	6,84359191	3,46730009	0,00111344	0,14907163	-0,86948255
TC0X00010341.hg.1	XKRX	402415	-0,70065472	4,97666839	-3,46417868	0,00112382	0,149379	-0,87729064
TC1400008752.hg.1	CIDEB	27141	0,63421587	8,78152337	3,461039	0,00113435	0,14970227	-0,88514049

TC0X00008862.hg.1	FUNDC2	65991	0,76626772	8,28103985	3,4550775	0,00115462	0,1504031	-0,90003459
TC1100009234.hg.1	TRAPPC4	51399	0,54083081	8,37562089	3,45468995	0,00115595	0,1504031	-0,90100235
TC2100008375.hg.1	KRTAP10-1	386677	-0,33445075	7,73344365	-3,45210493	0,00116485	0,15041589	-0,90745586
TC0900006969.hg.1	CHMP5	51510	0,62660186	8,80787418	3,44994805	0,00117233	0,15041589	-0,91283843
TC1800008474.hg.1	SLC39A6	25800	0,58901601	8,14718818	3,44686515	0,00118309	0,15075063	-0,9205287
TC1600006826.hg.1	ABAT	18	-0,28454558	7,6320314	-3,43794273	0,00121479	0,15226959	-0,94276386
TC0400012027.hg.1	ANAPC10	10393	0,51568695	6,0422112	3,43786524	0,00121507	0,15226959	-0,94295681
TC0500010497.hg.1	NADK2	133686	0,39337556	4,89969244	3,43657023	0,00121974	0,15226959	-0,94618134
TC0500010018.hg.1	CFAP90	134121	-0,26752961	5,07685882	-3,43425002	0,00122815	0,1522901	-0,95195682
TC0300011220.hg.1	CHDH	55349	-0,23656907	4,38422545	-3,42815623	0,00125049	0,15402692	-0,9671151
TC0X00011139.hg.1	CETN2	1069	0,73555841	7,31919981	3,42431918	0,00126476	0,1541143	-0,97665198
TC1500009795.hg.1	IGDCC3	9543	-0,33091609	4,97292682	-3,41936061	0,00128342	0,1541143	-0,98896744
TC0100008152.hg.1	UQCRH	7388	0,61938381	8,83738641	3,4192891	0,00128369	0,1541143	-0,98914496
TC0400007958.hg.1	PRDM8	56978	0,36809527	6,46021361	3,41675904	0,00129332	0,1541143	-0,99542483
TC1300009273.hg.1	COMMD6	170622	0,4424401	10,4851306	3,41487479	0,00130054	0,1541143	-1,00010003
TC2100008357.hg.1	CFAP410	755	-0,26189804	8,46879854	-3,41422386	0,00130304	0,1541143	-1,00171477
TC0X00008080.hg.1	VSIG1	340547	1,00913242	7,54636956	3,41252422	0,00130959	0,1541143	-1,00593022
TC0600014069.hg.1	TMEM14B	81853	0,543631	9,12199898	3,40656818	0,0013328	0,15522737	-1,02069295
TC1200008525.hg.1	SNRPF	6636	0,56772116	6,45717715	3,40579217	0,00133585	0,15522737	-1,02261528
TC0900009040.hg.1	SPACA9	11092	-0,33293642	5,75565951	-3,398272	0,00136578	0,15771315	-1,04123154
TC0400012551.hg.1	DCTD	1635	0,95614068	6,14273005	3,39536272	0,00137753	0,15796996	-1,04842722
TC1600011552.hg.1	CTRL	1506	-0,36901745	6,95294698	-3,3924986	0,00138919	0,15796996	-1,05550775
TC0500009481.hg.1	ATP6V0E1	8992	0,3782746	10,5310866	3,39140761	0,00139365	0,15796996	-1,05820393
TC0100006876.hg.1	MIIP	60672	-0,31938064	7,79476307	-3,38753227	0,00140963	0,15809564	-1,06777718
TC0500007542.hg.1	NDUFAF2	91942	0,62540156	7,15979188	3,38504683	0,00141996	0,15809564	-1,07391364
TC0100018463.hg.1	RHOC	389	1,15874343	7,40868231	3,38288885	0,001429	0,15809564	-1,07923955
TC0100013748.hg.1	GNL2	29889	0,53571865	7,45420406	3,37985178	0,0014418	0,15809564	-1,08673173
TC1900007871.hg.1	COX6B1	1340	0,31548029	10,7668786	3,37328165	0,00146988	0,15809564	-1,10292646
TC0100017229.hg.1	INTS7	25896	0,44928156	5,75002445	3,37205476	0,00147518	0,15809564	-1,10594863
TC1800006508.hg.1	MYL12B	103910	0,52009182	10,5635176	3,37092065	0,00148009	0,15809564	-1,10874168
TC1700011262.hg.1	SEPTIN4	5414	-0,45331384	5,2094521	-3,37051828	0,00148184	0,15809564	-1,10973249
TC0100016158.hg.1	PFDN2	5202	0,37629856	7,99837419	3,36808065	0,00149247	0,15809564	-1,11573357
TC1200012626.hg.1	PRPF40B	25766	-0,38173402	7,17104083	-3,36404789	0,00151022	0,15809564	-1,12565615
TC0800008546.hg.1	ENY2	56943	0,80129685	9,19643945	3,36341247	0,00151303	0,15809564	-1,12721897
TC0200016115.hg.1	ARL4C	10123	0,89129941	9,28319429	3,36212407	0,00151875	0,15809564	-1,13038727
TC1100009004.hg.1	FDX1	2230	0,4451585	7,04649325	3,36209659	0,00151887	0,15809564	-1,13045484

TC0200016706.hg.1	MRPS5	64969	0,65742808	7,87941135	3,35886718	0,00153331	0,15809564	-1,13839316
TC0100018185.hg.1	MICOS10	440574	0,45060461	10,6311756	3,35741086	0,00153986	0,15809564	-1,14197156
TC1500009621.hg.1	GTF2A2	2958	0,47632508	7,32290771	3,35432477	0,00155383	0,15809564	-1,14955156
TC1700008879.hg.1	MRPL58	3396	0,55167251	6,44064431	3,35078096	0,00157002	0,15809564	-1,15825083
TC1900011651.hg.1	SAXO5	374877	-0,33843837	7,5783814	-3,34924295	0,00157709	0,15809564	-1,16202464
TC1500010731.hg.1	LOC100419583	100419583	0,59137033	6,87176258	3,34919441	0,00157732	0,15809564	-1,16214373
TC0500010591.hg.1	MROH2B	133558	-0,26281289	3,99720088	-3,34810742	0,00158234	0,15809564	-1,16481025
TC2000010027.hg.1	PEDS1-UBE2V1	387522	0,51608684	8,21519825	3,34661949	0,00158923	0,15809564	-1,1684595
TC0800010926.hg.1	PAG1	55824	0,93650543	9,19995113	3,3454151	0,00159484	0,15809564	-1,17141268
TC0100013483.hg.1	TAF12	6883	0,68463506	6,62210655	3,3445808	0,00159873	0,15809564	-1,17345801
TC1800006506.hg.1	MYL12A	10627	0,46186563	10,9581887	3,34255518	0,00160822	0,15809564	-1,17842274
TC0700013050.hg.1	KCNH2	3757	0,38329402	5,42894243	3,34062555	0,0016173	0,15809564	-1,18315055
TC0500008814.hg.1	PAIP2	51247	0,39160088	10,370166	3,33892747	0,00162534	0,15809564	-1,18730975
TC0100006729.hg.1	PARK7	11315	0,48271213	10,9474474	3,33511464	0,00164353	0,15809564	-1,19664419
TC1900011837.hg.1	UQCR11	10975	0,4220573	10,8049958	3,33469458	0,00164554	0,15809564	-1,19767219
TC0300011939.hg.1	IFT57	55081	-0,55106228	5,66453673	-3,33285352	0,0016544	0,15809564	-1,20217686
TC1500009204.hg.1	UBR1	197131	-0,66049337	9,1358606	-3,33099269	0,00166341	0,15809564	-1,20672843
TC0900012179.hg.1	SPTAN1	6709	-0,67243895	7,78636845	-3,33021889	0,00166716	0,15809564	-1,20862068
TC1200011311.hg.1	NAP1L1	4673	0,58926428	9,77806072	3,32992767	0,00166858	0,15809564	-1,20933279
TC0500011529.hg.1	RIOK2	55781	-0,8133503	5,49362061	-3,32554184	0,00169004	0,15931226	-1,22005258
TC0200009949.hg.1	DCAF17	80067	0,76517873	6,43487466	3,32113174	0,00171189	0,15949492	-1,23082339
TC1100007874.hg.1	POLR2G	5436	0,50826155	8,8565696	3,32019219	0,00171658	0,15949492	-1,23311696
TC1000007878.hg.1	KIFBP	26128	0,29035843	4,16186734	3,31993193	0,00171788	0,15949492	-1,23375222
TC0300010315.hg.1	IQSEC1	9922	-0,59439518	6,71436996	-3,31525641	0,00174141	0,16087121	-1,2451597
TC1600009417.hg.1	ZC3H7A	29066	0,46179707	10,0017167	3,31294973	0,00175313	0,16114827	-1,25078413
TC0100015299.hg.1	ST7L	54879	1,00522854	6,90834444	3,30583118	0,00178977	0,16303333	-1,26812693
TC1000011025.hg.1	PPP3CB	5532	0,57226203	7,29659872	3,30431128	0,00179769	0,16303333	-1,27182698
TC1900006955.hg.1	UBL5	59286	0,43825971	10,7292866	3,30384864	0,00180011	0,16303333	-1,27295303
TC1100007935.hg.1	STIP1	10963	0,47557769	7,90193993	3,30190197	0,00181031	0,16315756	-1,27769019
TC0200016672.hg.1	RTN4	57142	1,04013358	8,69214238	3,29615665	0,00184074	0,1650949	-1,29166158
TC0200010757.hg.1	TESHL	101928327	-0,31514159	4,52855696	-3,29398197	0,00185239	0,16533668	-1,29694618
TC0600014068.hg.1	TMEM14C	51522	0,8438407	8,02533528	3,28789454	0,00188535	0,16732476	-1,31172803
TC2000008213.hg.1	DDRGK1	65992	0,42227361	8,24045897	3,28653863	0,00189277	0,16732476	-1,31501834
TC0X00007528.hg.1	OTUD6A	139562	-0,3064621	4,64425375	-3,28336696	0,00191023	0,16798751	-1,32271166
TC1200008764.hg.1	WSCD2	9671	0,31288779	5,328564	3,28166789	0,00191965	0,16798751	-1,32683119
TC0700007098.hg.1	GARS1	2617	0,44781639	9,16304307	3,27773973	0,00194159	0,16798751	-1,33635046

TC0500013389.hg.1	MED7	9443	0,8292849	7,83164226	3,27543889	0,00195455	0,16798751	-1,34192302
TC0100008090.hg.1	DMAP1	55929	-0,5519083	6,59001212	-3,27529665	0,00195535	0,16798751	-1,34226743
TC1000011690.hg.1	ARL3	403	0,63523486	7,86269481	3,27358485	0,00196505	0,16798751	-1,34641176
TC0200009402.hg.1	MGAT5	4249	-0,77182206	6,52907728	-3,27307472	0,00196795	0,16798751	-1,34764654
TC0900009829.hg.1	NDUFB6	4712	0,66986663	7,56282632	3,272187	0,00197301	0,16798751	-1,34979502
TC0800010766.hg.1	LACTB2	51110	0,7419643	5,76983009	3,26641313	0,00200619	0,17002891	-1,36376063
TC0200016667.hg.1	GPR75-ASB3	100302652	-0,43113561	6,6047608	-3,26403701	0,00201999	0,17041734	-1,36950362
TC1800009283.hg.1	SMAD2	4087	0,45279702	8,06288806	3,25851137	0,00205245	0,17236843	-1,38284922
TC1800007734.hg.1	ZNF236	7776	-0,44648346	9,29851285	-3,25504724	0,00207305	0,17274285	-1,3912089
TC0600014293.hg.1	CRIP3	401262	-0,47369849	6,5376543	-3,25461909	0,00207561	0,17274285	-1,39224175
TC1200007615.hg.1	DIP2B	57609	-0,62757326	9,73597096	-3,24581795	0,00212888	0,17638238	-1,41345517
TC1700010704.hg.1	ZNF385C	201181	-0,29384121	7,31150786	-3,23879447	0,00217233	0,17917809	-1,4303591
TC0500012467.hg.1	ARSI	340075	-0,33209817	5,61160745	-3,23497295	0,00219631	0,18035147	-1,43954734
TC0X00007686.hg.1	PBDC1	51260	0,44119866	7,009345	3,22980544	0,00222915	0,18223772	-1,45196141
TC0700012013.hg.1	GIGYF1	64599	-0,32444575	6,14241425	-3,22439194	0,00226404	0,18427503	-1,46495358
TC0900012067.hg.1	PNPLA7	375775	-0,47145879	7,73981583	-3,22198278	0,00227974	0,18473859	-1,47073121
TC0100018220.hg.1	CCDC30	728621	0,44533729	4,33143064	3,21841402	0,00230317	0,18534735	-1,47928496
TC2000009128.hg.1	ZHX3	23051	-0,5713359	6,73941684	-3,21778739	0,00230731	0,18534735	-1,48078629
TC1100007247.hg.1	CAPRIN1	4076	0,85273869	8,70951345	3,21450617	0,0023291	0,18555394	-1,48864485
TC0X00009069.hg.1	ARHGAP6	395	0,74087382	5,02068079	3,21437555	0,00232997	0,18555394	-1,4889576
TC0X00006590.hg.1	WWC3	55841	-0,66627362	6,65493355	-3,20162511	0,00241652	0,19113756	-1,51944799
TC0100012828.hg.1	LZIC	84328	0,70877111	6,80064903	3,19615387	0,00245458	0,19113756	-1,5325088
TC0300007164.hg.1	SS18L2	51188	0,45536003	8,76534594	3,19212335	0,00248298	0,19113756	-1,54212162
TC1900012055.hg.1	VN1R1	57191	-0,4600929	6,477677	-3,19174761	0,00248564	0,19113756	-1,54301739
TC0200012256.hg.1	HEATR5B	54497	-0,56015021	7,87287408	-3,19132185	0,00248866	0,19113756	-1,54403234
TC0600011560.hg.1	UQCC2	84300	0,50346081	6,26541269	3,19126792	0,00248904	0,19113756	-1,54416087
TC0700009222.hg.1	LRGUK	136332	-0,25396999	5,36646429	-3,19081614	0,00249225	0,19113756	-1,54523773
TC0200006440.hg.1	ACP1	52	0,5463122	7,98128972	3,1905236	0,00249433	0,19113756	-1,54593497
TC0600014198.hg.1	TULP4	56995	-0,57021584	9,04961238	-3,1901681	0,00249686	0,19113756	-1,54678223
TC1600010184.hg.1	ITFG1	81533	0,62450439	7,53440077	3,18923271	0,00250353	0,19113756	-1,54901125
TC0200016647.hg.1	DPY30	84661	0,55719127	9,47947467	3,18422022	0,00253956	0,19159202	-1,56094908
TC0600007495.hg.1	HLA-A	3105	-0,37841555	9,13553655	-3,18373025	0,00254311	0,19159202	-1,56211539
TC0400008175.hg.1	PDLIM5	10611	0,47869647	8,19853205	3,18095986	0,00256326	0,19159202	-1,56870783
TC1100012981.hg.1	RCN1	5954	0,73317501	6,3464773	3,18015494	0,00256914	0,19159202	-1,57062256
TC1100009433.hg.1	HYLS1	219844	0,56733668	4,83316533	3,17925592	0,00257573	0,19159202	-1,57276077
TC2000009731.hg.1	PSMA7	5688	0,34127069	10,3089218	3,17750765	0,00258858	0,19159202	-1,57691778

TC1100012919.hg.1	THYN1	29087	0,50525483	7,89090242	3,17653293	0,00259577	0,19159202	-1,57923485
TC0800011363.hg.1	BAALC-AS2	157556	-0,33326867	5,29786122	-3,17493543	0,0026076	0,19159202	-1,58303142
TC1500010769.hg.1	GOLGA6C	653641	-0,32811376	4,9312859	-3,1738574	0,00261561	0,19159202	-1,58559275
TC1100013097.hg.1	REXO2	25996	0,95437398	6,70078992	3,17354766	0,00261791	0,19159202	-1,58632859
TC0700011601.hg.1	TMEM60	85025	0,76594033	7,95920559	3,17114419	0,00263587	0,19159202	-1,59203683
TC1400010767.hg.1	ZFYVE26	23503	-0,38828681	8,15559962	-3,17038804	0,00264154	0,19159202	-1,59383214
TC1600011312.hg.1	HBZ	3050	-0,21338972	4,67675634	-3,16976309	0,00264624	0,19159202	-1,59531575
TC0800011239.hg.1	COX6C	1345	0,38883187	10,8729974	3,16822128	0,00265786	0,19159202	-1,59897516
TC1100007899.hg.1	PLAAT4	5920	0,71869743	9,8285255	3,16626317	0,00267269	0,19159202	-1,60362106
TC1000008643.hg.1	SCD	6319	-0,39937562	7,00977506	-3,1638873	0,00269079	0,19159202	-1,60925578
TC0100018265.hg.1	GSTM5	2949	0,31946411	5,01538462	3,16332823	0,00269506	0,19159202	-1,61058132
TC1600007803.hg.1	CNEP1R1	255919	0,89173024	7,32090022	3,16075666	0,00271481	0,19159202	-1,61667655
TC0400007293.hg.1	UBE2K	3093	0,55424686	9,09071004	3,1600319	0,0027204	0,19159202	-1,61839384
TC1600009580.hg.1	XYLT1	64131	-0,57182713	7,59065753	-3,15869285	0,00273076	0,19159202	-1,62156606
TC0200010009.hg.1	SCRN3	79634	0,3497894	5,07598157	3,15860826	0,00273142	0,19159202	-1,62176642
TC0100018498.hg.1	KRTCAP2	200185	0,41002263	10,4551384	3,15292236	0,00277582	0,19159202	-1,63522678
TC0X00010712.hg.1	THOC2	57187	0,58416787	7,24236292	3,15283903	0,00277648	0,19159202	-1,63542394
TC0300008111.hg.1	OR5K2	402135	-0,19060587	3,840054	-3,15106883	0,00279044	0,19159202	-1,63961143
TC1400008722.hg.1	ZFHX2	85446	-0,29979672	6,33530794	-3,15093048	0,00279154	0,19159202	-1,63993865
TC0200015695.hg.1	DIRC3	729582	-0,50531571	5,29163403	-3,14893933	0,00280733	0,19159202	-1,64464694
TC1700010507.hg.1	YWHAEP7	284100	0,36872654	7,23199512	3,14691308	0,00282349	0,19159202	-1,64943634
TC0300010077.hg.1	LMLN	89782	0,34578988	5,26225173	3,14558342	0,00283414	0,19159202	-1,65257818
TC1100012702.hg.1	TMEM218	219854	0,7766692	7,08410541	3,14249749	0,00285901	0,19159202	-1,6598667
TC0500007251.hg.1	FBXO4	26272	-0,37890509	6,76470309	-3,14178877	0,00286475	0,19159202	-1,66153998
TC0X00007946.hg.1	ARMCX4	100131755	-0,36374316	4,47525536	-3,14091734	0,00287182	0,19159202	-1,66359709
TC1200007558.hg.1	PRPH	5630	-0,29644128	5,40549613	-3,13972663	0,00288151	0,19159202	-1,66640731
TC1100012140.hg.1	DCUN1D5	84259	0,52691162	6,53400578	3,139707	0,00288167	0,19159202	-1,66645362
TC0500010553.hg.1	FYB1	2533	1,63528044	10,0668154	3,13803405	0,00289534	0,19159202	-1,67040085
TC1400010085.hg.1	TCL1A	8115	-0,9990395	7,42953325	-3,13769795	0,00289809	0,19159202	-1,6711937
TC0100009889.hg.1	VPS45	11311	0,96105171	6,87468303	3,13713512	0,00290271	0,19159202	-1,67252129
TC2200008257.hg.1	LRP5L	91355	-0,51411272	6,49507863	-3,13545524	0,00291652	0,19159202	-1,67648282
TC0200016433.hg.1	TMEM178A	130733	-0,27340383	5,68171672	-3,13535739	0,00291733	0,19159202	-1,67671355
TC0X00007224.hg.1	CCDC22	28952	-0,31860307	7,51895658	-3,13484319	0,00292157	0,19159202	-1,67792585
TC1200007137.hg.1	FGFR1OP2	26127	0,65812621	6,2206822	3,13451586	0,00292428	0,19159202	-1,67869752
TC1000010265.hg.1	ITGB1	3688	0,69760915	10,5520809	3,13171593	0,0029475	0,19180912	-1,68529624
TC0300012590.hg.1	PRR23B	389151	-0,3006094	4,87835391	-3,13161388	0,00294835	0,19180912	-1,68553667

TC1200011491.hg.1	EPYC	1833	-0,28099182	4,99802737	-3,12943634	0,00296654	0,19227107	-1,69066592
TC1900008655.hg.1	ZNF175	7728	0,41457592	6,70214185	3,12785413	0,00297983	0,19227107	-1,69439145
TC0X00007587.hg.1	PIN4	5303	0,47717536	7,85568472	3,12704076	0,00298667	0,19227107	-1,6963062
TC1700012468.hg.1	JPT1	51155	0,54591834	8,61843737	3,12174947	0,0030316	0,19419181	-1,70875468
TC0400008604.hg.1	EXOSC9	5393	0,63407944	8,99976468	3,12095299	0,00303841	0,19419181	-1,71062737
TC0300007335.hg.1	TMA7	51372	0,32702984	10,8731511	3,11983089	0,00304804	0,19419181	-1,71326514
TC0100011267.hg.1	ATP2B4	493	0,85925834	10,4349235	3,11724696	0,00307032	0,19443856	-1,71933706
TC0100007449.hg.1	SH3BGRL3	83442	0,72507447	7,91587285	3,11694195	0,00307296	0,19443856	-1,72005359
TC0100015939.hg.1	DCST2	127579	-0,29437506	5,16324298	-3,11349593	0,00310294	0,19444521	-1,72814588
TC1700010645.hg.1	KRTAP3-2	83897	-0,34513749	5,58911415	-3,11319138	0,0031056	0,19444521	-1,72886081
TC0700013457.hg.1	FSCN3	29999	-0,33826031	6,04540637	-3,11308632	0,00310652	0,19444521	-1,72910741
TC1100009900.hg.1	OR51G2	81282	-0,28961798	4,27292834	-3,11209955	0,00311517	0,19444521	-1,73142342
TC0700010247.hg.1	RPA3	6119	0,72003934	7,95367904	3,11024952	0,00313143	0,19480242	-1,7357643
TC0300013090.hg.1	PHC3	80012	-0,33607316	10,1388741	-3,10717282	0,00315866	0,19507366	-1,74297983
TC0100018162.hg.1	TNFRSF14	8764	-0,43141974	8,33278809	-3,10707431	0,00315954	0,19507366	-1,74321078
TC1200012806.hg.1	MYO1A	4640	-0,34228183	4,65512113	-3,10433997	0,00318393	0,19507366	-1,74961952
TC0200015012.hg.1	ATP5MC3	518	0,51637836	9,50824825	3,10384592	0,00318835	0,19507366	-1,75077708
TC0500013118.hg.1	OR2V1	26693	-0,27313205	4,99386344	-3,1038204	0,00318858	0,19507366	-1,75083688
TC1900011655.hg.1	PET100	100131801	0,39442736	10,8786651	3,10228619	0,00320237	0,19527026	-1,75443081
TC1200012829.hg.1	NDUFA12	55967	0,71195199	9,88100312	3,09827676	0,00323865	0,19655505	-1,76381774
TC1700008690.hg.1	PITPNC1	26207	0,92365246	7,22752761	3,09761078	0,00324471	0,19655505	-1,7653762
TC1200009221.hg.1	HIP1R	9026	-0,60147975	6,33575635	-3,09243614	0,00329219	0,19877961	-1,77747812
TC0900006892.hg.1	IFNK	56832	-0,31170164	4,09444389	-3,09052566	0,00330989	0,19919702	-1,78194294
TC0200012058.hg.1	OST4	100128731	0,49987563	10,6472625	3,08008433	0,00340818	0,20444665	-1,80631353
TC1000010493.hg.1	OR13A1	79290	-0,38054124	4,62740007	-3,07825713	0,00342566	0,20483021	-1,81057292
TC1200011991.hg.1	IQCD	115811	-0,31310049	6,67350082	-3,07424653	0,00346433	0,20647384	-1,81991642
TC0900009276.hg.1	NELFB	25920	-0,32818164	7,84682187	-3,0683939	0,00352148	0,20873803	-1,83353737
TC1900011915.hg.1	ZNF737	100129842	-0,52287693	7,54909398	-3,06804571	0,00352491	0,20873803	-1,83434722
TC1300006683.hg.1	GTF3A	2971	0,33062404	10,0142413	3,06472202	0,00355779	0,21001212	-1,84207459
TC1400007300.hg.1	PSMA3	5684	0,68992272	8,90947294	3,06194992	0,00358544	0,21014695	-1,84851546
TC1500009458.hg.1	ARPP19	10776	0,66977509	6,74483348	3,06017753	0,00360322	0,21014695	-1,85263158
TC1400010263.hg.1	CINP	51550	0,58617659	6,06205784	3,0600068	0,00360494	0,21014695	-1,85302801
TC0100011527.hg.1	NENF	29937	0,4298959	8,03041915	3,05858989	0,00361922	0,21014695	-1,85631737
TC1900010951.hg.1	SNRPD2	6633	0,27471446	10,5114202	3,05781098	0,00362709	0,21014695	-1,85812521
TC2000010026.hg.1	PEDS1	387521	0,35483504	7,79590778	3,05768914	0,00362832	0,21014695	-1,85840796
TC1500010255.hg.1	C15orf40	123207	-0,32202897	4,4215176	-3,0551924	0,00365367	0,21086051	-1,8642007

TC0300007470.hg.1	PPM1M	132160	0,52364471	8,3544781	3,05423228	0,00366347	0,21086051	-1,86642749
TC2000007149.hg.1	BPIFA1	51297	-0,24324896	4,68807422	-3,04868978	0,00372048	0,2134772	-1,87927333
TC1500009702.hg.1	RPS27L	51065	0,6740627	9,48472415	3,04640375	0,00374424	0,21417528	-1,88456732
TC0300013975.hg.1	IP6K2	51447	0,44381712	7,06100347	3,03729435	0,00384033	0,21846172	-1,90563746
TC0500011528.hg.1	LIX1	167410	-0,68375139	5,59883201	-3,0370608	0,00384283	0,21846172	-1,90617714
TC2200008300.hg.1	TFIP11	24144	0,39227243	6,21898034	3,03437293	0,00387164	0,21896748	-1,91238614
TC0600012550.hg.1	LYRM2	57226	0,65733616	5,69205113	3,03402067	0,00387543	0,21896748	-1,91319959
TC0200014834.hg.1	STK39	27347	0,85658373	7,80076684	3,03135986	0,00390417	0,21933419	-1,91934216
TC1400007761.hg.1	AHSA1	10598	0,95585216	8,12498229	3,03048662	0,00391365	0,21933419	-1,9213573
TC0300013123.hg.1	PLD1	5337	-0,2697062	5,82500534	-3,0301294	0,00391753	0,21933419	-1,92218152
TC0400012868.hg.1	STOX2	56977	-0,28494096	5,40286357	-3,02510261	0,00397256	0,22140255	-1,93377347
TC1900011748.hg.1	SRRM5	100170229	-0,30596957	4,86865138	-3,0245689	0,00397844	0,22140255	-1,9350035
TC1700011956.hg.1	TBC1D16	125058	-0,30161469	5,96266452	-3,02157607	0,00401159	0,22216163	-1,94189838
TC1900011636.hg.1	UBE2M	9040	0,32440429	8,85813969	3,02044504	0,00402418	0,22216163	-1,94450289
TC0X00011102.hg.1	CD99L2	83692	0,37873408	6,66699066	3,02008927	0,00402815	0,22216163	-1,94532202
TC0700008274.hg.1	ZNF804B	219578	-0,20060554	3,92999689	-3,01777811	0,00405403	0,22240987	-1,95064176
TC0400012252.hg.1	PPID	5481	0,48901319	6,02091163	3,01740126	0,00405826	0,22240987	-1,95150892
TC0800012287.hg.1	LEPROTL1	23484	0,85266797	9,33212613	3,01646778	0,00406877	0,22240987	-1,95365666
TC0400010058.hg.1	HS3ST1	9957	-0,38925717	7,2438279	-3,01440541	0,00409207	0,22254199	-1,95840015
TC1500009216.hg.1	TGM7	116179	-0,29298558	5,35924563	-3,01324314	0,00410525	0,22254199	-1,96107249
TC1400009998.hg.1	NDUFB1	4707	0,40936536	10,4581437	3,01306136	0,00410732	0,22254199	-1,96149039
TC1900007543.hg.1	ZNF257	113835	-0,32368552	6,91265832	-3,01181591	0,0041215	0,22265735	-1,96435312
TC0700008263.hg.1	DBF4	10926	-0,44904919	6,31943374	-3,00902071	0,00415349	0,22351581	-1,97077522
TC1700009706.hg.1	MFSD6L	162387	-0,22919017	6,75824054	-3,00786093	0,00416683	0,22351581	-1,97343871
TC1700006500.hg.1	TRARG1	286753	-0,27307529	5,85246245	-3,00726695	0,00417368	0,22351581	-1,97480258
TC0X00008405.hg.1	RBMX2	51634	0,45331536	7,98613103	3,00496353	0,00420034	0,22377997	-1,98008989
TC0900011177.hg.1	TXN	7295	0,75424535	10,7402421	3,0047484	0,00420284	0,22377997	-1,98058355
TC0500007378.hg.1	NDUFS4	4724	0,42548495	8,83063821	3,0019565	0,00423538	0,22481322	-1,9869883
TC0200012994.hg.1	SNRPG	6637	0,45524612	9,62879402	3,00043442	0,00425322	0,22481322	-1,99047838
TC0800006692.hg.1	MSRA	4482	0,49033829	7,8712454	2,9998206	0,00426044	0,22481322	-1,99188554
TC0800012432.hg.1	MRPS28	28957	0,75485401	6,85639236	2,99826556	0,00427877	0,22481322	-1,99544953
TC0400008902.hg.1	ABCE1	6059	0,82410512	8,20840275	2,99773821	0,004285	0,22481322	-1,99665788
TC1200009459.hg.1	PUS1	80324	-0,32043081	6,9273348	-2,99657831	0,00429874	0,22481322	-1,99931516
TC1600007335.hg.1	SBK1	388228	-0,27557381	4,32273948	-2,99584718	0,00430742	0,22481322	-2,00098981
TC1000012497.hg.1	BORCS7-ASMT	100528007	0,25555934	4,5036149	2,994041	0,00432894	0,2248963	-2,00512571
TC0X00007222.hg.1	PLP2	5355	0,84828111	8,5155239	2,9926742	0,00434528	0,2248963	-2,00825441

TC1900007189.hg.1	ZNF333	84449	-0,28549168	5,25459596	-2,99265376	0,00434553	0,2248963	-2,00830118
TC1900011890.hg.1	WDR83OS	51398	0,28541203	9,033424	2,98705183	0,00441314	0,22775754	-2,02111458
TC1000008736.hg.1	SUFU	51684	-0,21837489	6,77292681	-2,98414562	0,0044486	0,22863414	-2,02775581
TC0500008921.hg.1	NDFIP1	80762	0,54082465	8,66659006	2,9836339	0,00445488	0,22863414	-2,02892476
TC0300014082.hg.1	ETV5	2119	0,336764	4,04542785	2,98132773	0,00448324	0,22874821	-2,0341912
TC0X00009867.hg.1	SPIN4	139886	0,34007014	4,93529313	2,9810298	0,00448692	0,22874821	-2,03487135
TC0500008304.hg.1	SLC25A46	91137	0,452563	6,68667363	2,97693087	0,0045378	0,22874821	-2,04422458
TC0200014108.hg.1	NIFK	84365	0,45727953	8,5476239	2,97622255	0,00454664	0,22874821	-2,04584003
TC2000006577.hg.1	PRND	23627	-0,25004053	5,73939342	-2,97489134	0,00456331	0,22874821	-2,04887539
TC0800010103.hg.1	LSM12	124801	0,50804177	8,13636049	2,97399672	0,00457455	0,22874821	-2,05091475
TC1100011811.hg.1	CCDC90B	60492	0,88113054	7,1782811	2,97310665	0,00458575	0,22874821	-2,05294335
TC0200016654.hg.1	COX7A2L	9167	0,57112193	8,21086685	2,97054287	0,00461816	0,22874821	-2,05878434
TC2100007797.hg.1	ATP5PF	522	0,38952574	10,4268364	2,97025369	0,00462183	0,22874821	-2,05944297
TC1100008652.hg.1	TMEM126A	84233	0,95319158	8,44941471	2,97017589	0,00462282	0,22874821	-2,05962016
TC0200012082.hg.1	IFT172	26160	-0,74106741	7,81039503	-2,97003356	0,00462462	0,22874821	-2,0599443
TC0500012153.hg.1	FAM13B	51306	-0,53401761	6,51027401	-2,96998138	0,00462529	0,22874821	-2,06006314
TC0X00006593.hg.1	CLCN4	1183	-0,46109084	6,28018487	-2,96967776	0,00462914	0,22874821	-2,06075456
TC0100016049.hg.1	FCRL3	115352	-0,95446431	9,63882572	-2,96957653	0,00463043	0,22874821	-2,06098508
TC1500009438.hg.1	LYSMD2	256586	0,64796602	6,52445704	2,96835532	0,00464598	0,22890446	-2,06376557
TC1000009327.hg.1	MGMT	4255	-0,32618284	6,6894823	-2,96683583	0,0046654	0,22919417	-2,06722415
TC0600012123.hg.1	DST	667	0,82021369	5,07307204	2,96457398	0,00469445	0,22919417	-2,07237029
TC0600013519.hg.1	ZC3H12D	340152	-0,7647867	7,59385368	-2,9640402	0,00470133	0,22919417	-2,07358437
TC2000007294.hg.1	CTNNBL1	56259	0,67440394	9,93963983	2,96402812	0,00470148	0,22919417	-2,07361183
TC1300008225.hg.1	PSPC1	55269	-0,40838325	9,40337102	-2,96250981	0,0047211	0,22954497	-2,07706442
TC1700011731.hg.1	ATP5PD	10476	0,36222954	9,95803322	2,95601199	0,00480594	0,23286509	-2,09182702
TC0500013401.hg.1	LCP2	3937	0,9508601	9,47766243	2,95535464	0,0048146	0,23286509	-2,09331928
TC0400009746.hg.1	SLBP	7884	0,52584765	6,87295317	2,95403896	0,00483197	0,23309542	-2,09630539
TC1600007918.hg.1	IRX5	10265	-0,39472105	6,24968377	-2,95024199	0,00488246	0,23491183	-2,10491814
TC1600006600.hg.1	E4F1	1877	-0,38440564	6,77527249	-2,9492999	0,00489506	0,23491183	-2,10705397
TC0100016629.hg.1	RNASEL	6041	0,4570596	6,18228004	2,94771019	0,00491639	0,23530843	-2,11065703
TC0200009219.hg.1	MYO7B	4648	-0,64498676	5,31199155	-2,94570917	0,00494336	0,23530843	-2,11519047
TC1900011849.hg.1	DUS3L	56931	-0,28923183	7,94457754	-2,94438737	0,00496126	0,23530843	-2,11818397
TC0100006577.hg.1	RER1	11079	0,34055282	8,68419755	2,9439006	0,00496786	0,23530843	-2,11928615
TC0800009919.hg.1	GNRH1	2796	0,51036509	7,25687154	2,9435356	0,00497282	0,23530843	-2,12011252
TC1500009636.hg.1	ANXA2	302	1,20578485	8,15667942	2,9430271	0,00497974	0,23530843	-2,12126367
TC0200009065.hg.1	DBI	1622	0,47247928	8,27705837	2,94042992	0,00501519	0,23637942	-2,12714115

TC0100015397.hg.1	CD58	965	0,97072568	8,0349561	2,93659122	0,00506804	0,23826212	-2,13582195
TC0300009610.hg.1	NDUFB5	4711	0,73812351	9,1394537	2,93317205	0,00511554	0,23945394	-2,14354767
TC1000009659.hg.1	AKR1C8	340811	0,25706671	5,23633628	2,93268116	0,00512239	0,23945394	-2,14465637
TC0700013597.hg.1	SAP25	100316904	-0,34766449	8,30221583	-2,93070526	0,00515007	0,23945394	-2,14911776
TC0300006491.hg.1	EDEM1	9695	-0,61094866	5,98253257	-2,93019881	0,00515718	0,23945394	-2,15026094
TC0700012203.hg.1	COG5	10466	-0,5866788	8,12252462	-2,92858067	0,00517998	0,23945394	-2,15391263
TC0300009731.hg.1	MAP3K13	9175	-0,50761421	4,59777952	-2,9269251	0,0052034	0,23945394	-2,15764742
TC1300008632.hg.1	ALG5	29880	0,72863476	7,69325536	2,92620335	0,00521364	0,23945394	-2,15927514
TC1000009920.hg.1	VIM-AS1	100507347	0,96852144	7,04000108	2,92604021	0,00521596	0,23945394	-2,15964305
TC2200009360.hg.1	HDAC10	83933	-0,38534292	7,042779	-2,92573596	0,00522028	0,23945394	-2,1603291
TC0400011705.hg.1	C4orf3	401152	0,45173539	7,93987761	2,92312067	0,00525759	0,23945394	-2,16622442
TC1400009690.hg.1	ABCD4	5826	-0,48871705	6,38973084	-2,92261963	0,00526477	0,23945394	-2,16735347
TC0400008429.hg.1	ALPK1	80216	-0,44987003	4,15070996	-2,92252163	0,00526617	0,23945394	-2,16757428
TC1500010926.hg.1	ARPIN-AP3S2	100526783	0,48346338	6,54361015	2,92243073	0,00526748	0,23945394	-2,1677791
TC0700008579.hg.1	TRIM56	81844	-0,39609683	8,00712461	-2,92141769	0,00528202	0,23945394	-2,17006137
TC1300006930.hg.1	COG6	57511	-0,38451427	6,06444919	-2,92012232	0,00530067	0,23945394	-2,17297893
TC1300009013.hg.1	VPS36	51028	0,4511631	7,78051855	2,92011699	0,00530075	0,23945394	-2,17299094
TC0600006554.hg.1	FOXC1	2296	-0,22515853	4,8432727	-2,91919979	0,005314	0,23946675	-2,17505622
TC1600008889.hg.1	CPNE7	27132	-0,30541159	7,0576989	-2,91579242	0,00536347	0,24044076	-2,18272489
TC0400007311.hg.1	RHOH	399	-0,80101758	7,84973925	-2,91496747	0,00537551	0,24044076	-2,18458062
TC0400008882.hg.1	SMARCA5	8467	0,53768063	6,93344859	2,91430991	0,00538513	0,24044076	-2,18605958
TC0400008287.hg.1	CISD2	493856	0,50485844	6,9755486	2,9141366	0,00538766	0,24044076	-2,18644933
TC1200010798.hg.1	AAAS	8086	0,4300744	9,03795054	2,91172219	0,00542313	0,24144023	-2,19187756
TC0400009024.hg.1	ARFIP1	27236	0,82728754	6,36961439	2,91038346	0,00544288	0,24149297	-2,19488608
TC1900009548.hg.1	MYO1F	4542	0,69768682	9,59315432	2,9095754	0,00545484	0,24149297	-2,19670157
TC0200007388.hg.1	LINC01126	100129726	-0,35435208	6,29946214	-2,9089134	0,00546466	0,24149297	-2,19818866
TC0300012589.hg.1	PRR23A	729627	-0,28347272	4,49246714	-2,90808013	0,00547703	0,24149297	-2,20006015
TC1600011494.hg.1	ARL6IP1	23204	0,36580862	9,08155025	2,90723155	0,00548967	0,24149297	-2,20196568
TC1300009004.hg.1	ATP7B	540	0,24420835	4,32683075	2,90582233	0,0055107	0,24184256	-2,20512932
TC0200016420.hg.1	MRPL33	9553	0,66040929	8,81149518	2,90489132	0,00552464	0,24187975	-2,20721882
TC2000009895.hg.1	SMIM26	388789	0,47769212	8,64880647	2,90233351	0,00556311	0,24249193	-2,21295714
TC0100015866.hg.1	S100A4	6275	0,89677242	8,90457701	2,90221637	0,00556487	0,24249193	-2,21321984
TC0200015912.hg.1	IRS1	3667	-0,3002514	5,34260026	-2,90007125	0,00559733	0,24321877	-2,21802955
TC0100010510.hg.1	RCSD1	92241	-0,23962792	8,0606868	-2,89937666	0,00560788	0,24321877	-2,21958644
TC0200012522.hg.1	FBXO11	80204	-0,46494441	6,36871769	-2,8982982	0,0056243	0,24335941	-2,22200324
TC0700007398.hg.1	ZMIZ2	83637	-0,44164461	7,7403564	-2,89638583	0,00565351	0,24360517	-2,22628731

TC0200015216.hg.1	ORMDL1	94101	0,55596435	9,14539012	2,89620089	0,00565634	0,24360517	-2,22670152
TC1200012648.hg.1	MYL6	4637	0,31789447	11,1560927	2,89370783	0,00569466	0,24468507	-2,23228336
TC0300006437.hg.1	CHL1	10752	-0,25130914	3,70450018	-2,89136504	0,00573089	0,24535215	-2,23752582
TC0200009920.hg.1	SSB	6741	0,60553703	8,93750424	2,89098765	0,00573675	0,24535215	-2,23837003
TC0700010678.hg.1	LSM5	23658	0,7344662	6,01671734	2,88984613	0,00575449	0,24554276	-2,24092315
TC1600008656.hg.1	KLHL36	79786	-0,453052	5,76272016	-2,88772584	0,00578759	0,24587911	-2,24566359
TC1900008435.hg.1	SELENOW	6415	0,48532375	9,58545731	2,88533669	0,0058251	0,24587911	-2,25100231
TC1200009972.hg.1	GPR19	2842	0,31699684	5,21222858	2,88473141	0,00583464	0,24587911	-2,25235438
TC2000009975.hg.1	TMEM230	29058	0,29958193	9,96906403	2,88446841	0,00583879	0,24587911	-2,2529418
TC0900009944.hg.1	CD72	971	-0,97665531	7,95460279	-2,88433404	0,00584091	0,24587911	-2,2532419
TC1900011708.hg.1	PDCD2L	84306	0,6448189	7,31981193	2,88425061	0,00584222	0,24587911	-2,25342824
TC0600007412.hg.1	NKAPL	222698	-0,20832968	4,32273496	-2,88270789	0,00586663	0,24613674	-2,2568731
TC0600010008.hg.1	GTF2H5	404672	0,42384603	4,98112236	2,88187058	0,00587992	0,24613674	-2,25874227
TC0X00011353.hg.1	ASB9	140462	-0,47159775	5,49013423	-2,88049445	0,00590181	0,24613674	-2,26181349
TC0900011396.hg.1	RAB14	51552	0,39304359	9,62828537	2,88007874	0,00590844	0,24613674	-2,26274107
TC0800010154.hg.1	LSM1	27257	0,61390072	8,09676262	2,87967067	0,00591496	0,24613674	-2,26365152
TC0600011661.hg.1	STK38	11329	0,43439222	10,0168254	2,87734396	0,00595223	0,24699184	-2,26884095
TC0300012504.hg.1	KY	339855	-0,25163004	4,82301947	-2,87672106	0,00596224	0,24699184	-2,27022979
TC1200006782.hg.1	CLEC9A	283420	0,30621586	4,9651079	2,87462596	0,00599604	0,24783633	-2,27489953
TC0900012270.hg.1	FRRS1L	23732	-0,25435545	4,9144932	-2,87257532	0,0060293	0,24865459	-2,27946797
TC1900006993.hg.1	QTRT1	81890	-0,34094311	7,26022217	-2,87043103	0,00606425	0,24953924	-2,28424269
TC1700011967.hg.1	EIF4A3	9775	0,69880506	8,23926354	2,86948152	0,00607979	0,24962271	-2,28635621
TC1200012664.hg.1	MYRFL	196446	-0,25243348	4,30244827	-2,86591359	0,00613851	0,25091222	-2,29429382
TC1000008727.hg.1	NFKB2	4791	-0,3143198	6,86697185	-2,86480991	0,00615678	0,25091222	-2,29674785
TC1200010024.hg.1	ARHGDIB	397	0,34485058	10,621171	2,86446504	0,0061625	0,25091222	-2,29751453
TC0300007566.hg.1	SPATA12	353324	-0,28809153	5,2406977	-2,86428327	0,00616552	0,25091222	-2,29791859
TC1000008399.hg.1	IFIT1B	439996	-0,25713165	4,45551688	-2,85803816	0,00627	0,25413571	-2,31179069
TC0500013358.hg.1	SKP1	6500	0,47612476	8,91445042	2,85745949	0,00627976	0,25413571	-2,31307503
TC1500010900.hg.1	MTHFS	10588	0,73127295	7,01580506	2,85365329	0,00634433	0,25413571	-2,32151841
TC0X00010989.hg.1	LDOC1	23641	-0,33478028	6,37741835	-2,85191384	0,00637405	0,25413571	-2,32537451
TC0200009813.hg.1	PSMD14	10213	0,42896976	7,52399415	2,85190156	0,00637426	0,25413571	-2,32540172
TC0400010251.hg.1	DHX15	1665	0,51668017	8,80068735	2,85015146	0,00640429	0,25413571	-2,32927981
TC0900009996.hg.1	PAX5	5079	-0,68434589	7,60579283	-2,84954875	0,00641466	0,25413571	-2,330615
TC0200008573.hg.1	UNC50	25972	0,52397762	8,79051198	2,84935867	0,00641793	0,25413571	-2,33103605
TC1400010642.hg.1	SERPINA4	5267	-0,23861244	4,36340819	-2,84926446	0,00641956	0,25413571	-2,33124473
TC0600011438.hg.1	LTB	4050	-0,57864816	9,42997413	-2,84819346	0,00643804	0,25413571	-2,33361667

TC0900006953.hg.1	TMEM215	401498	-0,24724018	6,06278517	-2,84796382	0,00644201	0,25413571	-2,33412519
TC2200007312.hg.1	LGALS1	3956	0,76174603	9,94651782	2,84771911	0,00644624	0,25413571	-2,33466704
TC1100013066.hg.1	KRTAP5-10	387273	-0,28258594	5,44006136	-2,84621186	0,00647236	0,25413571	-2,33800374
TC0500007836.hg.1	S100Z	170591	0,74096822	5,40006585	2,84509207	0,00649183	0,25413571	-2,34048194
TC0800007431.hg.1	AP3M2	10947	0,59213244	7,08115795	2,84496053	0,00649412	0,25413571	-2,34077299
TC0900009051.hg.1	GTF3C5	9328	0,24416596	7,55669394	2,84256582	0,00653596	0,25413571	-2,34607029
TC1200012573.hg.1	RHNO1	83695	-0,67677772	5,66051933	-2,84250701	0,00653699	0,25413571	-2,34620035
TC1400006674.hg.1	C14orf119	55017	0,63157927	7,52763498	2,84237315	0,00653934	0,25413571	-2,34649635
TC0800007330.hg.1	DDHD2	23259	-0,52290838	5,5201491	-2,84236772	0,00653943	0,25413571	-2,34650836
TC2000009014.hg.1	NDRG3	57446	0,44495487	8,28888285	2,84090707	0,00656509	0,25413571	-2,34973778
TC1700012356.hg.1	RPL26	6154	0,3208494	9,60333667	2,84064502	0,0065697	0,25413571	-2,35031704
TC0900010925.hg.1	ZNF782	158431	-0,52109333	5,80921209	-2,84025706	0,00657653	0,25413571	-2,35117457
TC0100018271.hg.1	OLFML3	56944	-0,2423921	5,05440162	-2,84013354	0,00657871	0,25413571	-2,35144757
TC0100012497.hg.1	GNB1	2782	0,22314108	10,7413385	2,83882456	0,00660182	0,25413571	-2,35434016
TC1900007887.hg.1	KIRREL2	84063	-0,34181718	5,68290919	-2,8385448	0,00660677	0,25413571	-2,35495824
TC1100010863.hg.1	OR5M11	219487	-0,22462174	4,51577506	-2,83826671	0,0066117	0,25413571	-2,35557262
TC1000010930.hg.1	PCBD1	5092	0,52919567	6,81654456	2,83737276	0,00662755	0,25413571	-2,35754727
TC1100009369.hg.1	OR10G9	219870	-0,29999771	5,27108894	-2,83724212	0,00662987	0,25413571	-2,35783582
TC1700007771.hg.1	GSDMA	284110	-0,25493795	6,19186398	-2,83564246	0,00665833	0,25469822	-2,36136818
TC0100015715.hg.1	SF3B4	10262	0,51923686	9,00194315	2,83349776	0,00669666	0,25479832	-2,36610194
TC0200011919.hg.1	LDAH	60526	0,65637051	6,64605773	2,8331916	0,00670215	0,25479832	-2,36677749
TC1600009744.hg.1	NDUFAB1	4706	0,55426775	7,87291726	2,83318238	0,00670232	0,25479832	-2,36679784
TC1000009404.hg.1	PWWP2B	170394	-0,26468499	7,89773181	-2,83233455	0,00671754	0,25485264	-2,36866834
TC1200010569.hg.1	ASB8	140461	0,41030859	9,06138749	2,83016482	0,00675664	0,25529804	-2,37345352
TC0100018131.hg.1	OR2T10	127069	-0,33160371	4,48096026	-2,83014973	0,00675691	0,25529804	-2,37348681
TC0200015559.hg.1	PLEKHM3	389072	-0,5026109	7,3797182	-2,82830937	0,00679025	0,25603389	-2,3775436
TC2200008829.hg.1	POLR3H	171568	-0,37640204	7,21533164	-2,82707703	0,00681265	0,25635555	-2,38025908
TC1900007851.hg.1	FFAR1	2864	-0,34953709	7,84438697	-2,82344463	0,0068791	0,25832957	-2,38825847
TC0100008697.hg.1	CTH	1491	0,37041219	5,28300654	2,82167805	0,00691162	0,25902468	-2,39214632
TC0400010867.hg.1	EPHA5	2044	-0,25341485	4,63384589	-2,82084276	0,00692706	0,25907745	-2,39398404
TC1000011726.hg.1	SH3PXD2A	9644	-0,37011615	5,97092003	-2,81977255	0,00694687	0,25929373	-2,39633805
TC0500009098.hg.1	MYOZ3	91977	-0,28875757	5,94755889	-2,81819511	0,00697618	0,25986257	-2,39980664
TC1200009489.hg.1	FBRSL1	57666	-0,42450487	7,26001804	-2,815294	0,00703037	0,26135439	-2,40618234
TC0400012934.hg.1	SDAD1	55153	0,43593902	7,54220789	2,81331724	0,00706752	0,26155969	-2,41052401
TC1900011139.hg.1	SLC17A7	57030	-0,24718443	4,65386474	-2,81330938	0,00706767	0,26155969	-2,41054127
TC0300006894.hg.1	CMC1	152100	1,23656319	7,89873455	2,81274211	0,00707836	0,26155969	-2,4117868

TC1200008792.hg.1	UNG	7374	0,289049	6,18380576	2,81160426	0,00709986	0,26183038	-2,41428463
TC0800008352.hg.1	VPS13B	157680	-0,57555882	9,45012767	-2,81068518	0,00711727	0,2619495	-2,4163017
TC0600006779.hg.1	KU-MEL-3	497048	-0,2326434	4,61459695	-2,80835452	0,00716159	0,26276238	-2,4214147
TC0800012285.hg.1	HMBOX1	79618	-0,4238532	6,14836952	-2,80802909	0,0071678	0,26276238	-2,42212838
TC0800009237.hg.1	GPAA1	8733	-0,29238775	8,15546777	-2,80667613	0,00719367	0,26303093	-2,42509493
TC1200008820.hg.1	IFT81	28981	0,2193226	4,06508304	2,80615784	0,0072036	0,26303093	-2,42623108
TC1300006698.hg.1	PDX1	3651	-0,26680268	6,08041602	-2,80461754	0,00723319	0,26328462	-2,42960676
TC1700009036.hg.1	TMC8	147138	-0,44716996	8,2705682	-2,80431336	0,00723904	0,26328462	-2,43027323
TC0400008007.hg.1	GPAT3	84803	-0,33427291	4,5579231	-2,80341947	0,00725628	0,26339304	-2,43223152
TC0100010100.hg.1	CKS1B	1163	0,4394364	7,84505272	2,79844035	0,00735299	0,26602526	-2,44313165
TC1700012345.hg.1	CTDNEP1	23399	0,31425184	8,22672828	2,79820494	0,00735759	0,26602526	-2,44364668
TC0600006524.hg.1	FOXQ1	94234	-0,26625632	6,47702184	-2,79576629	0,00740542	0,26723159	-2,4489801
TC1000008800.hg.1	GSTO1	9446	0,9530816	7,26187223	2,79436098	0,00743311	0,26770801	-2,4520521
TC1700007796.hg.1	IGFBP4	3487	-0,40113325	7,02236419	-2,79113249	0,0074971	0,26945396	-2,45910553
TC0300012029.hg.1	NAA50	80218	0,64419907	9,00273466	2,79044628	0,00751076	0,26945396	-2,46060398
TC0100008752.hg.1	TYW3	127253	0,5144594	6,66093743	2,78687542	0,00758224	0,27070375	-2,4683975
TC0400009723.hg.1	CTBP1	1487	-0,45123746	8,96295466	-2,78657889	0,0075882	0,27070375	-2,46904439
TC1200009300.hg.1	BRI3BP	140707	-0,4540049	8,17571144	-2,78623091	0,00759521	0,27070375	-2,46980343
TC0100009479.hg.1	DCLRE1B	64858	0,3669943	5,96288244	2,78565463	0,00760682	0,27070375	-2,47106034
TC1900011327.hg.1	ZNF160	90338	-0,35666953	6,35569251	-2,78505826	0,00761885	0,27070375	-2,47236086
TC1700012473.hg.1	FBF1	85302	-0,21576972	7,42196223	-2,78297426	0,00766105	0,27168046	-2,47690402
TC1400009131.hg.1	MAP4K5	11183	0,49815714	7,25767978	2,7796592	0,00772861	0,27240664	-2,48412603
TC0800008629.hg.1	RAD21-AS1	644660	-0,23149099	5,34463701	-2,77947535	0,00773237	0,27240664	-2,48452638
TC0700008747.hg.1	HBP1	26959	-0,40281595	9,32369731	-2,77891254	0,0077439	0,27240664	-2,48575184
TC1200012791.hg.1	FMNL3	91010	-0,57587974	6,98028691	-2,77884838	0,00774522	0,27240664	-2,48589153
TC1700010190.hg.1	IFT20	90410	0,4984173	6,85153807	2,77829935	0,00775649	0,27240664	-2,48708679
TC0500008054.hg.1	POLR3G	10622	0,74974714	5,88936824	2,77752833	0,00777233	0,27240664	-2,48876506
TC0800007774.hg.1	CHD7	55636	-0,64233605	7,3941769	-2,77692587	0,00778473	0,27240664	-2,49007621
TC0900006554.hg.1	INSL4	3641	-0,25893511	4,58466063	-2,77539827	0,00781626	0,27299287	-2,49339988
TC0600007534.hg.1	PRR3	80742	-0,29274631	6,9279699	-2,77241702	0,00787813	0,27300173	-2,49988265
TC0X00008081.hg.1	ATG4A	115201	0,45045671	5,37469753	2,77187098	0,00788951	0,27300173	-2,5010695
TC0400012874.hg.1	CYP4V2	285440	0,62803837	6,69723889	2,76956478	0,00793775	0,27300173	-2,50608035
TC2200007419.hg.1	TNRC6B	23112	-0,36698351	9,07952856	-2,7691613	0,00794621	0,27300173	-2,50695671
TC1100008651.hg.1	TMEM126B	55863	0,50923067	7,99171658	2,76834016	0,00796347	0,27300173	-2,50873999
TC0X00010382.hg.1	BEX5	340542	0,34268291	5,41178058	2,76803	0,00797	0,27300173	-2,50941346
TC0200009537.hg.1	KYNU	8942	0,82386578	5,29266013	2,76784259	0,00797395	0,27300173	-2,50982037

TC1600009182.hg.1	ZNF200	7752	0,36622916	4,87319305	2,76778339	0,00797519	0,27300173	-2,50994892
TC1400008684.hg.1	PRMT5	10419	0,40204351	7,15607331	2,76749448	0,00798128	0,27300173	-2,51057617
TC1600011345.hg.1	DNASE1	1773	-0,39717608	7,70087272	-2,76665967	0,0079989	0,27300173	-2,51238835
TC1100009370.hg.1	OR10G8	219869	-0,19199261	4,58239138	-2,76664323	0,00799924	0,27300173	-2,51242405
TC0400007978.hg.1	ENOPH1	58478	0,37668767	7,1988059	2,76658137	0,00800055	0,27300173	-2,51255831
TC0100015236.hg.1	LAMTOR5	10542	0,41760568	10,2935239	2,76590084	0,00801494	0,27300173	-2,51403529
TC1400006658.hg.1	MRPL52	122704	0,40702735	7,41159756	2,76503821	0,00803322	0,27300173	-2,51590711
TC1500007905.hg.1	CIMAP1C	161753	-0,24696827	5,31343252	-2,76423754	0,00805021	0,27300173	-2,51764412
TC1700009539.hg.1	PFN1	5216	0,31361843	11,8636595	2,76183778	0,00810135	0,27300173	-2,5228482
TC1900011049.hg.1	TPRX1	284355	-0,26149644	7,22442029	-2,76163894	0,0081056	0,27300173	-2,52327924
TC1600006574.hg.1	NDUFB10	4716	0,27701654	8,70054819	2,76084803	0,00812253	0,27300173	-2,52499361
TC1100012478.hg.1	CD3D	915	1,69873421	8,936178	2,76082573	0,00812301	0,27300173	-2,52504194
TC1000010065.hg.1	ARHGAP21	57584	0,60542174	6,32527801	2,76082033	0,00812313	0,27300173	-2,52505366
TC1000008582.hg.1	C10orf62	414157	-0,32749753	4,8314529	-2,7604379	0,00813132	0,27300173	-2,52588247
TC1900011642.hg.1	SPPL2B	56928	-0,55126044	8,31708907	-2,75984645	0,00814402	0,27300173	-2,52716415
TC0100008885.hg.1	SPATA1	100505741	-0,43661257	4,2683013	-2,75892757	0,00816378	0,27300173	-2,52915498
TC1000009063.hg.1	DENND10	404636	0,5076549	8,49233175	2,75858561	0,00817114	0,27300173	-2,52989574
TC0400006936.hg.1	C1QTNF7	114905	-0,22260297	5,47919958	-2,75672838	0,00821124	0,27384633	-2,53391781
TC0900012141.hg.1	GCNT1	2650	-0,49086791	4,4845201	-2,75355363	0,00828021	0,27482025	-2,54078874
TC1500007520.hg.1	APH1B	83464	0,68983848	8,21366835	2,75273091	0,00829817	0,27482025	-2,54256842
TC1400007155.hg.1	RTRAF	51637	0,43793035	9,14613384	2,75265568	0,00829981	0,27482025	-2,54273112
TC0600011631.hg.1	TEAD3	7005	-0,25152323	6,71049941	-2,75143972	0,00832643	0,27482025	-2,5453607
TC1700012246.hg.1	STARD3	10948	-0,35506228	6,87269916	-2,75106695	0,0083346	0,27482025	-2,54616666
TC2000006756.hg.1	MACROD2	140733	-0,73519027	4,41151429	-2,75067938	0,00834311	0,27482025	-2,54700456
TC1000012575.hg.1	ATAD1	84896	-0,56702681	7,95501658	-2,75061321	0,00834456	0,27482025	-2,5471476
TC1000010249.hg.1	KIF5B	3799	0,51030687	7,40187648	2,74926472	0,00837423	0,27526047	-2,55006221
TC0600013179.hg.1	OR2A4	79541	-0,44243619	5,10919401	-2,74865276	0,00838773	0,27526047	-2,55138457
TC0800010737.hg.1	SLCO5A1	81796	-0,3819357	5,46253888	-2,74378833	0,00849572	0,27822002	-2,56188852
TC0300008766.hg.1	COPG1	22820	0,4880207	7,97982287	2,74296458	0,00851413	0,27822002	-2,56366597
TC0100016263.hg.1	TMCO1	54499	0,3927256	8,27021493	2,74232854	0,00852837	0,27822002	-2,56503816
TC0100015918.hg.1	UBE2Q1	55585	0,52123725	9,17066735	2,74189278	0,00853814	0,27822002	-2,56597812
TC1000007092.hg.1	MASTL	84930	0,66494235	6,29247843	2,73975149	0,00858631	0,27916792	-2,57059553
TC0100007199.hg.1	VWA5B1	127731	-0,26201167	5,21214036	-2,73833712	0,00861826	0,27916792	-2,57364403
TC0200013001.hg.1	ADD2	119	-0,46859679	5,89000554	-2,73705022	0,00864742	0,27916792	-2,57641684
TC0X00009218.hg.1	RPS6KA3	6197	-0,51351318	5,09964802	-2,73667643	0,00865591	0,27916792	-2,57722203
TC0200008617.hg.1	PDCL3	79031	0,50172156	6,01321446	2,73651085	0,00865967	0,27916792	-2,57757871

TC1900009226.hg.1	LINGO3	645191	-0,23550172	5,1882204	-2,73593752	0,00867271	0,27916792	-2,57881355
TC0700010509.hg.1	CYCS	54205	0,59854268	8,86635954	2,73592478	0,008673	0,27916792	-2,57884099
TC0100007398.hg.1	TMEM50A	23585	0,55876293	9,5412996	2,73424329	0,00871135	0,27991465	-2,58246157
TC0900010886.hg.1	PTCH1	5727	0,89969427	7,26081255	2,73192755	0,00876442	0,28113099	-2,58744523
TC1100006666.hg.1	MMP26	56547	-0,26862115	4,08475887	-2,7311617	0,00878204	0,28120785	-2,58909276
TC0200016000.hg.1	GPR55	9290	0,43411574	4,94237419	2,73015561	0,00880523	0,28146268	-2,5912566
TC1700008677.hg.1	CACNG4	27092	-0,21560783	5,0776167	-2,72767945	0,00886255	0,28275223	-2,59657979
TC0100008921.hg.1	CLCA2	9635	-0,12325685	3,48032453	-2,72701667	0,00887795	0,28275223	-2,59800403
TC1900008921.hg.1	ZNF865	100507290	-0,30266113	6,63359937	-2,72567232	0,00890927	0,28275223	-2,60089217
TC1100006455.hg.1	B4GALNT4	338707	-0,23407845	6,50601964	-2,72524099	0,00891933	0,28275223	-2,60181859
TC0800009819.hg.1	DOK2	9046	0,99342007	10,5105158	2,72489883	0,00892733	0,28275223	-2,60255344
TC1000009879.hg.1	RPP38-DT	221060	-0,25219237	4,81995293	-2,72333289	0,008964	0,28275223	-2,60591569
TC1500008370.hg.1	ST8SIA2	8128	-0,20924158	5,22702171	-2,72316691	0,0089679	0,28275223	-2,60627198
TC0500009268.hg.1	TTC1	7265	0,6149348	6,06581971	2,72316239	0,008968	0,28275223	-2,60628169
TC0200014609.hg.1	RBM43	375287	-0,35043878	5,95057025	-2,72199537	0,00899543	0,28287221	-2,6087864
TC0X00009776.hg.1	FAM120C	54954	-0,31080001	7,37130792	-2,72169832	0,00900243	0,28287221	-2,60942383
TC0100018273.hg.1	ATP1A1	476	0,82403647	8,23633829	2,72056953	0,00902905	0,28322714	-2,61184559
TC1600007536.hg.1	ARMC5	79798	-0,25025463	6,94308797	-2,71979966	0,00904725	0,28331706	-2,61349691
TC0900011811.hg.1	DDX31	64794	-0,22776467	4,55255449	-2,71719061	0,00910919	0,28415845	-2,61909067
TC0300014094.hg.1	DYNLT2B	255758	0,6707661	7,47467893	2,7162168	0,0091324	0,28415845	-2,62117754
TC0500011211.hg.1	OTP	23440	-0,30721846	6,40745955	-2,71598013	0,00913805	0,28415845	-2,62168464
TC1300008963.hg.1	DLEU7	220107	-0,22582742	6,99420707	-2,71555921	0,00914811	0,28415845	-2,62258646
TC1200011773.hg.1	APPL2	55198	-0,41126794	6,8284839	-2,71543749	0,00915102	0,28415845	-2,62284721
TC0100016112.hg.1	TAGLN2	8407	0,38688141	10,5078642	2,71021621	0,00927667	0,28757663	-2,63402505
TC0200013278.hg.1	GGCX	2677	0,34659741	8,80398404	2,70858972	0,00931613	0,28781717	-2,63750399
TC1900008035.hg.1	NFKBIB	4793	-0,30645987	5,38321774	-2,70826839	0,00932394	0,28781717	-2,63819111
TC0900012151.hg.1	ANKRD19P	138649	-0,23241603	4,02067887	-2,70797189	0,00933116	0,28781717	-2,63882509
TC1600008845.hg.1	CDT1	81620	-0,29949881	6,79872735	-2,7070243	0,00935426	0,2878266	-2,64085088
TC1500010899.hg.1	ST20-MTHFS	100528021	0,66627597	7,93737005	2,70668158	0,00936262	0,2878266	-2,64158345
TC0600007830.hg.1	MAPK13	5603	-0,25101261	5,83356106	-2,70508668	0,00940165	0,28854618	-2,64499166
TC0100017523.hg.1	LIN9	286826	0,2808443	3,85190785	2,70247607	0,00946585	0,2899731	-2,65056732
TC1700007095.hg.1	DRC3	83450	0,22419943	4,56276636	2,70126443	0,00949578	0,2899731	-2,6531538
TC1800007682.hg.1	CNDP1	84735	-0,26879386	4,89092151	-2,70045241	0,00951589	0,2899731	-2,65488677
TC0900008178.hg.1	NANS	54187	0,65934488	8,01502084	2,69909267	0,00954965	0,2899731	-2,6577878
TC0900008532.hg.1	DELEC1	50514	-0,24001021	5,03679152	-2,69893572	0,00955355	0,2899731	-2,65812259
TC1100013027.hg.1	COX8A	1351	0,37649992	10,9657034	2,69871654	0,00955901	0,2899731	-2,6585901

TC0700007054.hg.1	PRR15	222171	-0,29029221	7,60344996	-2,69844095	0,00956587	0,2899731	-2,65917789
TC1400009353.hg.1	SIX4	51804	-0,21844995	4,79875482	-2,6981269	0,0095737	0,2899731	-2,65984766
TC0100017043.hg.1	LRRN2	10446	-0,29299211	6,23516918	-2,69584094	0,00963083	0,29085818	-2,66472124
TC1100006731.hg.1	ILK	3611	0,5070265	8,35004959	2,69549686	0,00963946	0,29085818	-2,66545456
TC1700012276.hg.1	PNPO	55163	0,39796562	5,92492586	2,69403223	0,00967627	0,29085818	-2,66857527
TC1100008042.hg.1	CCDC85B	11007	-0,23110865	6,04348875	-2,69388509	0,00967997	0,29085818	-2,66888871
TC0100015597.hg.1	ANKRD34A	284615	-0,26223892	5,32909979	-2,69372496	0,009684	0,29085818	-2,66922982
TC0100018090.hg.1	LINC02897	388759	-0,31873926	6,472222	-2,69299305	0,00970246	0,29085818	-2,67078873
TC0200007015.hg.1	RAB10	10890	0,68813289	9,57299547	2,69229038	0,0097202	0,29085818	-2,67228508
TC0600012554.hg.1	BACH2	60468	-0,92965098	6,79352755	-2,6919482	0,00972886	0,29085818	-2,67301366
TC1900008522.hg.1	SNRNP70	6625	-0,40057354	9,15989811	-2,68884546	0,00980764	0,2927399	-2,67961708
TC1500007318.hg.1	TEX9	374618	0,56412504	4,81925529	2,68786691	0,00983261	0,29277351	-2,68169858
TC1600008627.hg.1	HSBP1	3281	0,75700683	7,21730902	2,68717802	0,00985022	0,29277351	-2,6831636
TC0200008262.hg.1	RNF181	51255	0,27329838	6,41375259	2,68694038	0,00985631	0,29277351	-2,6836689
TC1400007354.hg.1	PPM1A	5494	0,72321583	8,10687735	2,68584844	0,0098843	0,29313374	-2,68599037
TC1200011909.hg.1	VPS29	51699	0,2853652	10,3024551	2,68377544	0,00993764	0,29412209	-2,69039572
TC1600008832.hg.1	IL17C	27189	-0,25615123	6,61675686	-2,68331747	0,00994947	0,29412209	-2,69136863

Supplementary Table 5. List of differentially expressed genes in myeloid cells between post-treatment and pre-treatment with belimumab.

Affymetrix ID	Gene Symbol	Entrez	logFC	AveExpr	t	P.Value	adj.P.Val
TC0100009241.hg.1	S1PR1	1901	1,7710954	9,73623675	7,68043213	6,40E-10	1,18E-05
TC0100016135.hg.1	SLAMF6	114836	2,06395859	9,32261902	6,4552219	4,88E-08	0,00045117
TC1300009249.hg.1	KLF12	11278	1,56015878	8,36154703	6,26238813	9,67E-08	0,00059552
TC1200006771.hg.1	CLEC2D	29121	1,12110719	10,0494301	6,00488117	2,40E-07	0,00110965
TC1200010516.hg.1	SLC38A1	81539	1,81013491	8,52316038	5,91778169	3,27E-07	0,00118338
TC1100012225.hg.1	NPAT	4863	1,19145255	8,18329066	5,8715509	3,84E-07	0,00118338
TC0100017110.hg.1	FCMR	9214	1,64664016	9,49861149	5,58470165	1,05E-06	0,0026507
TC1900010761.hg.1	CEACAM4	1089	-0,43538048	7,69972607	-5,55950981	1,15E-06	0,0026507
TC1400007430.hg.1	SYNE2	23224	1,30077735	8,30642396	5,44241264	1,73E-06	0,00347259
TC2200009272.hg.1	APOBEC3D	140564	1,26471454	8,45256593	5,41794812	1,88E-06	0,00347259
TC1400008193.hg.1	EVL	51466	0,8484444	10,153371	5,38415404	2,11E-06	0,00355015
TC2000009458.hg.1	NFATC2	4773	1,15154947	8,85648973	5,30780914	2,75E-06	0,00424045
TC1000011904.hg.1	ABLIM1	3983	1,64864754	9,40195691	5,22538634	3,66E-06	0,00490112
TC1900011908.hg.1	CIST1	729966	-0,90845605	6,04502441	-5,20803476	3,89E-06	0,00490112
TC0400012874.hg.1	CYP4V2	285440	1,17713301	6,69723889	5,19099834	4,12E-06	0,00490112
TC0300008559.hg.1	PARP15	165631	1,45265838	9,57012364	5,16835237	4,46E-06	0,00490112
TC1700010982.hg.1	SKAP1	8631	1,25264765	8,21786678	5,16496784	4,51E-06	0,00490112
TC1900008882.hg.1	NCR1	9437	1,09155479	7,19527997	5,13738385	4,96E-06	0,00508951
TC0600007301.hg.1	BTN3A2	11118	0,57487494	11,2918282	5,07368211	6,17E-06	0,00600006
TC0600012763.hg.1	SCML4	256380	0,94071302	8,48403798	5,0435163	6,84E-06	0,00632045
TC0400009919.hg.1	MRFAP1L1	114932	0,51747727	8,96747029	5,0174248	7,48E-06	0,00632205
TC1500009056.hg.1	RASGRP1	10125	1,48531868	8,12359	5,01557392	7,53E-06	0,00632205
TC0200008534.hg.1	ANKRD36	375248	1,00120591	8,4311606	4,96970517	8,80E-06	0,00685302
TC2000007928.hg.1	ZNF831	128611	0,94668371	6,81444081	4,95125726	9,38E-06	0,00685302
TC0700007409.hg.1	CCM2	83605	0,84951161	8,96775861	4,9469855	9,51E-06	0,00685302
TC1100008557.hg.1	USP35	57558	-0,73876848	8,17965357	-4,94106937	9,71E-06	0,00685302
TC2000006628.hg.1	MCM8	84515	0,87661361	5,19305391	4,93015146	1,01E-05	0,00685302
TC0200016424.hg.1	LBH	81606	1,24969303	9,01151779	4,92118591	1,04E-05	0,00685302
TC2000006559.hg.1	CDC25B	994	0,7752952	8,65986302	4,89858024	1,12E-05	0,00714513
TC0400010636.hg.1	OCIAD2	132299	1,28736969	8,8305221	4,82969686	1,42E-05	0,00872386
TC0100010243.hg.1	PYHIN1	149628	1,36145775	9,00545493	4,77146047	1,72E-05	0,01027777
TC1100013193.hg.1	PTPRCAP	5790	1,03578906	9,35315416	4,74945496	1,86E-05	0,01072296
TC0900011962.hg.1	NACC2	138151	-0,47690135	6,21894601	-4,72693801	2,00E-05	0,01121651

TC0400012782.hg.1	STIM2	57620	0,70791391	9,79051383	4,69970404	2,20E-05	0,01192975
TC1400007710.hg.1	JDP2	122953	-0,9151626	6,47751063	-4,68418701	2,31E-05	0,01220825
TC0900010886.hg.1	PTCH1	5727	1,53769655	7,26081255	4,66922568	2,43E-05	0,01247954
TC0100016337.hg.1	XCL2	6846	1,4777484	5,42027915	4,65297092	2,57E-05	0,01282148
TC1100010175.hg.1	RRAS2	22800	1,41086353	7,46220029	4,62477578	2,82E-05	0,01371826
TC1000008081.hg.1	KAT6B	23522	0,74144037	8,02046022	4,60670294	3,00E-05	0,0141978
TC2100007821.hg.1	ADAMTS1	9510	1,03613713	4,49005111	4,58743934	3,20E-05	0,01442654
TC1200010252.hg.1	TMTC1	83857	-0,62952332	7,43356975	-4,58545857	3,22E-05	0,01442654
TC1500009263.hg.1	PATL2	197135	1,08100429	9,04772366	4,57967534	3,28E-05	0,01442654
TC1000009172.hg.1	BUB3	9184	0,54346355	9,09934127	4,55389147	3,57E-05	0,01487496
TC1900011915.hg.1	ZNF737	100129842	0,7756311	7,54909398	4,5511124	3,61E-05	0,01487496
TC0600007303.hg.1	BTN3A1	11119	0,71374446	9,54785846	4,54973455	3,62E-05	0,01487496
TC1900009369.hg.1	PLIN3	10226	-0,37542614	7,38737378	-4,51791623	4,03E-05	0,01617258
TC0600008760.hg.1	PM20D2	135293	0,9809405	7,2838617	4,46160096	4,85E-05	0,01826181
TC0200011690.hg.1	YWHAQ	10971	0,48090089	10,0972751	4,45871192	4,90E-05	0,01826181
TC0100015265.hg.1	DENND2D	79961	1,4121997	8,30158392	4,45645415	4,93E-05	0,01826181
TC0100017078.hg.1	RAB29	8934	0,73476693	7,94445208	4,45598228	4,94E-05	0,01826181
TC1000007199.hg.1	ZEB1	6935	0,96485344	7,40492304	4,4449225	5,13E-05	0,01856903
TC1000006816.hg.1	OPTN	10133	0,64175684	7,96133189	4,4379308	5,25E-05	0,01863658
TC0500012138.hg.1	SPOCK1	6695	-0,39797994	5,71944691	-4,40480436	5,85E-05	0,02039145
TC0X00009981.hg.1	IL2RG	3561	0,78282736	9,51683093	4,39049188	6,13E-05	0,0204036
TC1600006656.hg.1	MMP25	64386	-0,43290162	7,4150713	-4,38617346	6,22E-05	0,0204036
TC0100015817.hg.1	THEM4	117145	1,13631729	6,74179435	4,38321101	6,28E-05	0,0204036
TC2200009248.hg.1	KREMEN1	83999	-0,46047674	5,62010512	-4,37778592	6,39E-05	0,0204036
TC1700011032.hg.1	GNGT2	2793	0,88232068	6,52661935	4,37716905	6,41E-05	0,0204036
TC0400008641.hg.1	FAT4	79633	0,63571853	5,26281736	4,34269879	7,17E-05	0,02245528
TC0X00008437.hg.1	STK26	51765	0,48596332	8,68143024	4,31077897	7,96E-05	0,02306383
TC0300006791.hg.1	KAT2B	8850	0,8532021	9,451382	4,30892232	8,01E-05	0,02306383
TC0200013567.hg.1	ANKRD36B	57730	0,77066195	9,15139725	4,30446475	8,12E-05	0,02306383
TC1300006794.hg.1	RXFP2	122042	-0,28700447	3,84670558	-4,30360342	8,15E-05	0,02306383
TC0300013809.hg.1	RPL14	9045	0,26607412	11,6769114	4,30309929	8,16E-05	0,02306383
TC0200016115.hg.1	ARL4C	10123	1,14074655	9,28319429	4,30307865	8,16E-05	0,02306383
TC0X00008073.hg.1	PRPS1	5631	0,71319363	9,40852458	4,29709948	8,32E-05	0,02306383
TC0100014457.hg.1	JAK1	3716	0,58815712	10,656396	4,29555818	8,36E-05	0,02306383
TC0700007345.hg.1	STK17A	9263	0,92815481	8,70583447	4,27864306	8,84E-05	0,02361689
TC0600007711.hg.1	ITPR3	3710	0,83741352	6,82253003	4,27688763	8,89E-05	0,02361689

TC1400010777.hg.1	TC2N	123036	1,94769315	7,78935582	4,27277452	9,01E-05	0,02361689
TC1600008661.hg.1	CRISPLD2	83716	-0,92544162	6,25063118	-4,27046089	9,08E-05	0,02361689
TC1500010080.hg.1	IMP3	55272	0,64145083	9,35519268	4,2487021	9,74E-05	0,02499439
TC2100006976.hg.1	ITSN1	6453	-0,75449778	5,72976513	-4,24433668	9,88E-05	0,02500364
TC0400012956.hg.1	ELOVL6	79071	1,11058906	5,54844883	4,23102323	0,00010315	0,02575368
TC1700010497.hg.1	SYNRG	11276	0,7983865	9,90425116	4,22562496	0,00010497	0,02585854
TC0300007484.hg.1	STAB1	23166	-0,99510008	6,97209267	-4,20405336	0,00011256	0,02721438
TC1700012460.hg.1	ABCA5	23461	1,01882183	7,26972899	4,201693	0,00011342	0,02721438
TC0100016049.hg.1	FCRL3	115352	1,34836204	9,63882572	4,19509061	0,00011586	0,02744467
TC0600013780.hg.1	LPAL2	80350	0,7261231	7,13250348	4,18497203	0,00011971	0,02799702
TC0600007306.hg.1	BTN3A3	10384	0,38553173	8,96543838	4,18000574	0,00012164	0,02809357
TC0200007609.hg.1	SPTBN1	6711	1,03445925	9,83363731	4,16306631	0,00012847	0,0293036
TC1500009641.hg.1	RORA	6095	1,27924039	9,01391645	4,14579731	0,00013581	0,02964113
TC0300008385.hg.1	TIGIT	201633	0,7058378	6,5541418	4,14439495	0,00013642	0,02964113
TC0900009275.hg.1	STPG3	441476	-0,4039399	7,36130626	-4,1435471	0,0001368	0,02964113
TC0200014834.hg.1	STK39	27347	1,17054816	7,80076684	4,142447	0,00013728	0,02964113
TC0600007833.hg.1	PNPLA1	285848	-0,38734607	4,67204942	-4,14088692	0,00013797	0,02964113
TC0X00006658.hg.1	OFD1	8481	0,77223382	9,8152029	4,11457235	0,00015013	0,03132792
TC0600008247.hg.1	IL17A	3605	-0,3167191	5,08927984	-4,11395151	0,00015043	0,03132792
TC0X00010155.hg.1	ITM2A	9452	0,93195873	8,16995207	4,11295821	0,00015091	0,03132792
TC1100007899.hg.1	PLAAT4	5920	0,93252849	9,8285255	4,10830828	0,00015317	0,03144491
TC1100008133.hg.1	TBC1D10C	374403	0,74748434	8,3290978	4,10065924	0,00015697	0,03187062
TC0100010532.hg.1	XCL1	6375	1,06398992	5,74220521	4,08891643	0,00016298	0,03243435
TC0600014337.hg.1	GOPC	57120	0,61555239	6,20653376	4,085511	0,00016477	0,03243435
TC1200010976.hg.1	AGAP2	116986	0,94184644	7,91233919	4,0842544	0,00016543	0,03243435
TC0600007108.hg.1	RNF144B	255488	-1,22648895	6,81944535	-4,08172859	0,00016677	0,03243435
TC1000007226.hg.1	CCDC7	79741	1,13551989	7,45396716	4,07192822	0,00017207	0,03311711
TC0200016710.hg.1	ANKRD36C	400986	0,93314291	8,25159405	4,0629434	0,00017708	0,03372905
TC1200007535.hg.1	CACNB3	784	-0,45627658	6,03749259	-4,05970026	0,00017892	0,03373198
TC1200012754.hg.1	KLRC1	3821	1,69735482	5,10171844	4,05344804	0,00018252	0,03406345
TC1000012582.hg.1	RRP12	23223	-0,51717511	7,67449538	-4,04565989	0,00018711	0,0340829
TC0X00009100.hg.1	TRAPPC2	6399	0,97328139	7,35500658	4,04533159	0,0001873	0,0340829
TC1200012748.hg.1	CD163L1	283316	-0,33813662	4,22400952	-4,04191644	0,00018935	0,0340829
TC0400009396.hg.1	NEIL3	55247	-0,40824196	6,81351793	-4,04083191	0,00019001	0,0340829
TC1200006773.hg.1	KLRF1	51348	2,10194689	5,3673049	4,03541226	0,00019331	0,03434235
TC0800006983.hg.1	PPP3CC	5533	0,86746954	9,4472857	4,01890528	0,00020372	0,03584762

TC1000010499.hg.1	ZFAND4	93550	0,78307217	7,04447775	4,01563373	0,00020585	0,03588023
TC1200006555.hg.1	CCND2	894	0,9707155	9,79304854	4,00528471	0,00021272	0,03656588
TC1400007732.hg.1	VASH1	22846	-0,69279801	5,204236	-4,00377677	0,00021374	0,03656588
TC0900010444.hg.1	CARNMT1	138199	0,83448251	8,45650065	3,98314276	0,00022818	0,03867759
TC0600007832.hg.1	BRPF3	27154	0,4489411	7,22965864	3,96697098	0,00024015	0,04033654
TC0900011501.hg.1	NR6A1	2649	-0,3891289	4,73125296	-3,95440694	0,00024987	0,04140424
TC1900010951.hg.1	SNRPD2	6633	0,35513727	10,5114202	3,95298682	0,00025099	0,04140424
TC0600007693.hg.1	RPS18	6222	0,19242301	12,3702697	3,94710064	0,00025569	0,04180667
TC0100014794.hg.1	ODF2L	57489	1,02525814	7,96919535	3,93668318	0,00026422	0,04219585
TC0500013039.hg.1	ADAMTS2	9509	-0,49692517	5,99400715	-3,93241629	0,0002678	0,04219585
TC0200008501.hg.1	ITPRIPL1	150771	0,66266005	5,49804235	3,93071894	0,00026923	0,04219585
TC1000008713.hg.1	NOLC1	9221	0,38107677	9,63613907	3,92907119	0,00027063	0,04219585
TC1700012387.hg.1	SLFN12L	100506736	1,33049977	7,37099906	3,9280953	0,00027146	0,04219585
TC1700008912.hg.1	MYO15B	80022	-0,48194011	7,46705172	-3,92555236	0,00027364	0,04219585
TC1600008166.hg.1	CTCF	10664	0,388641	8,13252372	3,92424881	0,00027477	0,04219585
TC0400006661.hg.1	STK32B	55351	-0,29765197	5,36136602	-3,92243099	0,00027634	0,04219585
TC1200006850.hg.1	ETV6	2120	-0,49790476	9,98057541	-3,91569236	0,00028226	0,04224022
TC0100007638.hg.1	SERINC2	347735	-0,63193605	6,20498328	-3,91470365	0,00028314	0,04224022
TC1300008371.hg.1	CENPJ	55835	0,63498208	4,9814116	3,91430394	0,00028349	0,04224022
TC1900007975.hg.1	ZNF570	148268	0,7050749	5,7978724	3,90916034	0,00028811	0,04258469
TC1900007743.hg.1	ZNF507	22847	1,0070185	6,52945279	3,90172734	0,00029491	0,04259623
TC0X00009160.hg.1	RBBP7	5931	0,80854713	9,67568792	3,90099105	0,00029559	0,04259623
TC1800007863.hg.1	ENOSF1	55556	0,8431797	6,86027377	3,89878752	0,00029764	0,04259623
TC0200008099.hg.1	HK2	3099	-0,53543204	6,08562895	-3,89758932	0,00029876	0,04259623
TC1200008792.hg.1	UNG	7374	0,40057819	6,18380576	3,89645822	0,00029982	0,04259623
TC2200008110.hg.1	PPM1F	9647	-0,5924114	6,52729395	-3,8917658	0,00030427	0,04259623
TC0200015349.hg.1	HSPD1	3329	1,32986556	7,89926611	3,89108149	0,00030492	0,04259623
TC1700007997.hg.1	GRN	2896	-0,60922812	9,6420641	-3,88887528	0,00030703	0,04259623
TC0200007929.hg.1	GMCL1	64395	0,61998728	6,41459207	3,88690342	0,00030894	0,04259623
TC1700008886.hg.1	SLC16A5	9121	-0,33066028	7,97459778	-3,88134194	0,00031436	0,04283446
TC0100015593.hg.1	ANKRD35	148741	-0,36002992	5,24605228	-3,88039057	0,0003153	0,04283446
TC1100006481.hg.1	DRD4	1815	-0,33607934	6,33489766	-3,87792007	0,00031775	0,04285182
TC0X00009867.hg.1	SPIN4	139886	0,44165444	4,93529313	3,87151025	0,00032418	0,04340286
TC0300007100.hg.1	SLC25A38	54977	0,80165532	7,81029837	3,86246181	0,00033348	0,0440056
TC0X00009869.hg.1	ARHGEF9	23229	0,7323687	8,68126461	3,86088688	0,00033513	0,0440056
TC0100013205.hg.1	ECE1	1889	-0,42467367	9,85947726	-3,85987603	0,00033619	0,0440056

TC2000009461.hg.1	ATP9A	10079	0,31559435	4,34739028	3,8549996	0,00034134	0,0440056
TC0200015631.hg.1	IKZF2	22807	1,09680537	6,25062925	3,8543935	0,00034199	0,0440056
TC0100018521.hg.1	TSTD1	100131187	0,91945271	8,14702716	3,85347082	0,00034298	0,0440056
TC0800007431.hg.1	AP3M2	10947	0,8007689	7,08115795	3,84737561	0,00034956	0,04441411
TC1500010778.hg.1	IL16	3603	0,50187826	9,80620349	3,8421071	0,00035535	0,04441411
TC1300008837.hg.1	ZC3H13	23091	0,48627509	9,55637512	3,8417213	0,00035578	0,04441411
TC0400009330.hg.1	SAP30	8819	-0,60225929	6,80301658	-3,83959854	0,00035814	0,04441411
TC0600011943.hg.1	ENPP5	59084	1,58051842	5,22220879	3,83956121	0,00035818	0,04441411
TC0600011546.hg.1	CUTA	51596	0,43944263	11,0374308	3,82414121	0,00037579	0,04628751
TC1900011856.hg.1	CD320	51293	0,54105701	7,8163082	3,82059216	0,00037996	0,04649126
TC1300009583.hg.1	DOCK9	23348	0,63554244	7,45413606	3,8170489	0,00038417	0,0466969
TC1200006718.hg.1	CLEC6A	93978	-0,6945892	5,41404942	-3,811503	0,00039085	0,0469632
TC0900007663.hg.1	CEP78	84131	1,67126959	6,88416985	3,81101102	0,00039145	0,0469632
TC0200008090.hg.1	HTRA2	27429	0,51104989	8,28498821	3,80822465	0,00039485	0,04706568
TC1100012775.hg.1	ETS1	2113	0,76971912	9,4767699	3,79971364	0,00040541	0,04746451
TC1000008385.hg.1	STAMBPL1	57559	0,99197061	8,75007987	3,79558481	0,00041064	0,04746451
TC0700013338.hg.1	GLCCI1	113263	1,00584426	6,64340746	3,79421097	0,00041239	0,04746451
TC1100009791.hg.1	ASCL2	430	-0,31799613	6,86841605	-3,79395382	0,00041272	0,04746451
TC0200016502.hg.1	IL18R1	8809	1,08610601	6,15960896	3,79370139	0,00041304	0,04746451
TC0400012634.hg.1	CFAP97	57587	0,66577194	8,1723297	3,7932614	0,00041361	0,04746451
TC1300006989.hg.1	AKAP11	11215	0,48219342	8,07926553	3,78548787	0,00042369	0,04783118
TC1700010637.hg.1	KRT26	353288	-0,35002275	4,36924704	-3,78504342	0,00042427	0,04783118
TC0200014407.hg.1	DARS1	1615	0,68406242	8,8218384	3,78481773	0,00042457	0,04783118
TC1200011791.hg.1	CKAP4	10970	-0,58940768	7,32233111	-3,78184324	0,0004285	0,04798111
TC0300011108.hg.1	NPRL2	10641	0,73620782	6,99683723	3,7734922	0,00043971	0,04894032
TC0700010504.hg.1	OSBPL3	26031	0,75535534	7,21089859	3,77008616	0,00044437	0,04898954
TC0900009259.hg.1	UAP1L1	91373	-0,49249367	6,1044515	-3,76788199	0,0004474	0,04898954
TC1400007944.hg.1	KCNK13	56659	-0,42587684	5,9779701	-3,76737286	0,00044811	0,04898954
TC0X00010643.hg.1	SEPTIN6	23157	0,47623101	10,6772268	3,7634196	0,00045361	0,04923752
TC1300009288.hg.1	KCTD12	115207	-0,39722187	7,3408553	-3,7619299	0,00045571	0,04923752
TC1100009729.hg.1	TOLLIP	54472	-0,35843789	8,48799998	-3,75297156	0,00046848	0,05032363
TC1400008333.hg.1	PPP2R5C	5527	0,82567959	9,5905756	3,75024214	0,00047244	0,05045573
TC1700011234.hg.1	CUEDC1	404093	-0,49644403	7,92162523	-3,74730464	0,00047674	0,05056077
TC2000006577.hg.1	PRND	23627	-0,31477525	5,73939342	-3,74508149	0,00048002	0,05056077
TC0800011305.hg.1	NCALD	83988	0,77289362	7,25066666	3,7439905	0,00048164	0,05056077
TC1900006620.hg.1	GNA15	2769	-0,39373062	7,81502824	-3,73819501	0,00049031	0,05118074

TC0700008252.hg.1	CROT	54677	0,79914269	4,62694642	3,73027416	0,00050241	0,05194223
TC1600006658.hg.1	IL32	9235	1,39780919	9,03986036	3,72827828	0,00050551	0,05194223
TC1100009287.hg.1	ARHGEF12	23365	1,08376244	7,35008839	3,72793497	0,00050604	0,05194223
TC2200009153.hg.1	DENND6B	414918	-0,30556412	5,40067345	-3,71996391	0,00051859	0,0523859
TC1700010426.hg.1	SLFN13	146857	1,19379531	7,7871564	3,71924401	0,00051974	0,0523859
TC0300012654.hg.1	TFDP2	7029	1,02700858	7,21742921	3,71502764	0,00052652	0,0523859
TC0400007617.hg.1	POLR2B	5431	0,42888862	9,43222493	3,71500197	0,00052656	0,0523859
TC1900011030.hg.1	SLC8A2	6543	-0,31587594	4,92291653	-3,71439249	0,00052755	0,0523859
TC0300011899.hg.1	CBLB	868	0,69526594	9,52651165	3,71417767	0,00052789	0,0523859
TC2100008559.hg.1	CFAP298	56683	0,60926709	8,97137349	3,71275147	0,00053021	0,0523859
TC0300012186.hg.1	MIX23	131076	0,68539222	6,88402345	3,70708105	0,00053952	0,05282876
TC0700006629.hg.1	FSCN1	6624	-0,50945098	6,61214685	-3,70445537	0,00054388	0,05282876
TC0800012176.hg.1	PLEC	5339	-0,62063459	7,69389092	-3,70373153	0,00054509	0,05282876
TC1100011833.hg.1	SYTL2	54843	0,72305649	7,77671629	3,70122046	0,0005493	0,05282876
TC1200011882.hg.1	TRPV4	59341	-0,29110548	4,19502684	-3,6988902	0,00055324	0,05282876
TC0100011270.hg.1	LAX1	54900	1,27114962	8,11799298	3,698195	0,00055442	0,05282876
TC0500008627.hg.1	SLC22A4	6583	-0,43153667	6,11654096	-3,69689897	0,00055663	0,05282876
TC1100010761.hg.1	FNBP4	23360	0,4246576	9,99502274	3,69634808	0,00055757	0,05282876
TC0700010167.hg.1	ACTB	60	-0,35390631	8,4060698	-3,68937368	0,00056961	0,05369397
TC1300008533.hg.1	HSPH1	10808	0,62486822	9,25135892	3,68609596	0,00057535	0,05396004
TC1400008619.hg.1	ZNF219	51222	-0,45978055	5,58593485	-3,68439479	0,00057835	0,0539677
TC0700012224.hg.1	LAMB1	3912	-0,24165664	3,78411835	-3,68161411	0,00058329	0,05415519
TC1700008859.hg.1	CD300LD-AS1	146723	-0,39483124	4,96001707	-3,67175049	0,00060114	0,05516924
TC1500009173.hg.1	EHD4	30844	-0,49451394	6,05594241	-3,67067322	0,00060313	0,05516924
TC0200016647.hg.1	DPY30	84661	0,64222765	9,47947467	3,67018364	0,00060403	0,05516924
TC2200009271.hg.1	APOBEC3C	27350	0,87052519	9,24089592	3,6682121	0,00060768	0,05516924
TC0100012895.hg.1	MTHFR	4524	-0,38456725	6,66756702	-3,66661901	0,00061064	0,05516924
TC0100015058.hg.1	SASS6	163786	0,71808276	5,76372624	3,66403955	0,00061547	0,05516924
TC0700007285.hg.1	CDK13	8621	0,65554057	8,79655624	3,66299528	0,00061743	0,05516924
TC1900006493.hg.1	ELANE	1991	-0,4496075	5,15972445	-3,66263894	0,0006181	0,05516924
TC1400009190.hg.1	GNPNAT1	64841	0,85468297	5,67318155	3,65954924	0,00062395	0,05542395
TC1700008635.hg.1	RGS9	8787	1,56149665	6,59870802	3,65523683	0,00063221	0,05543497
TC0900012124.hg.1	POLR1E	64425	0,53898211	7,30535987	3,65517202	0,00063234	0,05543497
TC2200008637.hg.1	IL2RB	3560	1,29782791	8,60190644	3,65402099	0,00063456	0,05543497
TC2200009290.hg.1	TAFA5	25817	-0,32872312	6,87992374	-3,65323637	0,00063608	0,05543497
TC1100008218.hg.1	TPCN2	219931	-0,55860188	7,97352337	-3,64482032	0,0006526	0,05641196

TC1200006570.hg.1	KCNA1	3736	-0,29105966	4,99799753	-3,64441804	0,0006534	0,05641196
TC0500012390.hg.1	JAKMIP2	9832	0,69657527	5,72903056	3,64205756	0,00065811	0,05655446
TC1100007935.hg.1	STIP1	10963	0,52305128	7,90193993	3,63150774	0,00067957	0,05801049
TC0100007333.hg.1	PITHD1	57095	0,50667879	8,35871651	3,63034876	0,00068196	0,05801049
TC0100009040.hg.1	RPAP2	79871	0,76890113	6,11355262	3,62914131	0,00068447	0,05801049
TC1000009880.hg.1	NMT2	9397	0,64309814	7,06560027	3,6255184	0,00069205	0,05838462
TC0400010682.hg.1	SGCB	6443	0,57696251	5,3765837	3,62325609	0,00069682	0,05851986
TC0500013258.hg.1	ARHGEF37	389337	-0,31135443	5,16304633	-3,61944798	0,00070492	0,05893235
TC0300007748.hg.1	MITF	4286	-0,27496851	5,18373728	-3,61759172	0,0007089	0,05899826
TC0600013409.hg.1	HIVEP2	3097	0,6954924	7,181689	3,61339797	0,00071797	0,05948557
TC0700011642.hg.1	HGF	3082	-0,4464255	6,04414668	-3,60982041	0,0007258	0,05986579
TC1200007647.hg.1	ACVR1B	91	-0,49767443	5,37895047	-3,60675674	0,00073257	0,06015561
TC0100006926.hg.1	PRAMEF20	645425	-0,24562721	4,63520717	-3,59845922	0,00075121	0,06120065
TC1000007761.hg.1	ARID5B	84159	1,03060707	8,49352716	3,59814627	0,00075192	0,06120065
TC0400011815.hg.1	MFSD8	256471	0,8247266	6,8724793	3,59529377	0,00075844	0,06131735
TC1700007474.hg.1	RNF135	84282	-0,50114244	7,41109079	-3,59461749	0,00076	0,06131735
TC0900007848.hg.1	NXNL2	158046	-0,37557734	7,19762932	-3,59171406	0,0007667	0,0615892
TC0600011525.hg.1	RXRB	6257	0,42146389	8,97018083	3,58816947	0,00077496	0,06198324
TC1900008044.hg.1	NCCRP1	342897	-0,37076095	6,15781094	-3,58069636	0,00079265	0,06291729
TC2200009273.hg.1	APOBEC3F	200316	0,80458694	7,93512972	3,58036461	0,00079345	0,06291729
TC1100012677.hg.1	OR8B12	219858	-0,31251823	4,64228919	-3,57213994	0,00081339	0,06411604
TC0300014020.hg.1	CLDND1	56650	0,6971418	10,0343994	3,57020869	0,00081814	0,06411604
TC0200006757.hg.1	TRIB2	28951	0,76870688	7,09453094	3,56986947	0,00081898	0,06411604
TC1000010651.hg.1	CSTF2T	23283	0,44940411	6,04913247	3,56317605	0,00083566	0,06514656
TC0900012277.hg.1	AK1	203	0,57950697	7,6066108	3,55971576	0,00084442	0,06537485
TC2100007072.hg.1	TTC3	7267	0,46823894	8,66178269	3,55922441	0,00084567	0,06537485
TC0X00007200.hg.1	SUV39H1	6839	0,65074291	7,56368007	3,55380921	0,00085957	0,06543256
TC1900011845.hg.1	MFSD12	126321	-0,42345839	7,1287963	-3,55198489	0,0008643	0,06543256
TC0100018416.hg.1	TAS1R2	80834	-0,35522257	5,06188772	-3,55177276	0,00086485	0,06543256
TC0800009919.hg.1	GNRH1	2796	0,6156773	7,25687154	3,55092478	0,00086706	0,06543256
TC2200006768.hg.1	VPREB1	7441	-0,38058867	4,7344004	-3,54986418	0,00086983	0,06543256
TC1000008658.hg.1	PAX2	5076	-0,38096051	5,88258717	-3,54919188	0,00087159	0,06543256
TC1200012646.hg.1	ESYT1	23344	0,49707363	8,64242969	3,54853583	0,00087331	0,06543256
TC1500009120.hg.1	RMDN3	55177	0,64963271	7,97362499	3,54798812	0,00087475	0,06543256
TC0300011183.hg.1	NEK4	6787	-0,40760162	5,80951585	-3,54238982	0,00088959	0,06627446
TC1200010265.hg.1	CAPRIN2	65981	0,69288022	8,16004227	3,5401626	0,00089556	0,06645139

TC0900007143.hg.1	ZCCHC7	84186	0,72879964	8,44166585	3,53863999	0,00089967	0,06648891
TC0400009543.hg.1	TLR3	7098	0,73049908	4,63059316	3,53374364	0,00091299	0,06701783
TC0600014238.hg.1	BLOC1S5	63915	0,59743013	7,92155254	3,53334573	0,00091408	0,06701783
TC1000008310.hg.1	LDB3	11155	-0,33464176	6,84194423	-3,53157578	0,00091894	0,06710837
TC1900010008.hg.1	IQCN	80726	-1,23009743	7,64816837	-3,52774199	0,00092957	0,06739624
TC2000007936.hg.1	PHACTR3	116154	-0,29071042	5,91617482	-3,52752305	0,00093018	0,06739624
TC1100012187.hg.1	KBTBD3	143879	0,43397786	5,64236082	3,52613117	0,00093407	0,06741369
TC1900009583.hg.1	ZNF266	10781	0,65145762	7,0650758	3,52065394	0,00094953	0,06826265
TC0900010789.hg.1	IARS1	3376	0,52478766	6,97916775	3,51653744	0,00096131	0,06879106
TC1900011889.hg.1	MAN2B1	4125	-0,4249873	8,94112	-3,51549008	0,00096433	0,06879106
TC1200009199.hg.1	B3GNT4	79369	-0,30287758	5,69796352	-3,51177383	0,00097511	0,06921369
TC0100016024.hg.1	SH2D2A	9047	0,57154437	7,01098264	3,51087277	0,00097774	0,06921369
TC0100009328.hg.1	PRPF38B	55119	0,44151162	8,08529387	3,50612683	0,00099172	0,06970001
TC1200007740.hg.1	HOXC8	3224	-0,29969391	5,39540852	-3,50523552	0,00099437	0,06970001
TC0500013349.hg.1	FBN2	2201	-0,3911754	6,11712254	-3,50352646	0,00099946	0,06970001
TC1700007716.hg.1	MLLT6	4302	0,52705167	8,46846629	3,50344434	0,0009997	0,06970001
TC1100008342.hg.1	INPPL1	3636	-0,37339362	8,31399617	-3,49522318	0,00102455	0,06998868
TC1600007541.hg.1	AHSP	51327	-0,23631227	4,24269483	-3,49501507	0,00102519	0,06998868
TC0100009903.hg.1	RPRD2	23248	0,72227275	8,12500916	3,49494856	0,0010254	0,06998868
TC0900008742.hg.1	ARPC5L	81873	0,73149493	7,32352266	3,49419454	0,00102771	0,06998868
TC1900009682.hg.1	ELOF1	84337	-0,44860918	5,12580387	-3,49217778	0,00103391	0,06998868
TC1900008864.hg.1	KIR3DX1	90011	0,52516197	6,15532771	3,49190197	0,00103476	0,06998868
TC0100018485.hg.1	ADAMTSL4-AS1	574406	-0,51997216	6,79681187	-3,49128347	0,00103667	0,06998868
TC1100007729.hg.1	DTX4	23220	-0,7051435	5,99430314	-3,49038428	0,00103946	0,06998868
TC2000008007.hg.1	RPS21	6227	0,29173035	12,8395539	3,48866219	0,00104481	0,06998868
TC1200006737.hg.1	PHC1	1911	0,55395293	7,26678954	3,48845145	0,00104547	0,06998868
TC1100013164.hg.1	PRG2	5553	-0,34753316	6,04399375	-3,48807516	0,00104664	0,06998868
TC2000009961.hg.1	SCRT2	85508	-0,28752528	6,31853293	-3,48722414	0,0010493	0,06998868
TC2200007556.hg.1	MPPED1	758	-0,39273662	6,31725338	-3,48468863	0,00105726	0,07012064
TC0200011919.hg.1	LDAH	60526	0,80718594	6,64605773	3,48417915	0,00105887	0,07012064
TC0100018486.hg.1	CDC42SE1	56882	0,36306155	10,0653538	3,48099951	0,00106895	0,07053539
TC0900009654.hg.1	RPS6	6194	0,15872889	12,8787503	3,47784803	0,00107903	0,07094726
TC0700007905.hg.1	AUTS2	26053	1,09592063	7,68798101	3,47509081	0,00108793	0,07122422
TC0200016369.hg.1	DTYMK	1841	0,42959135	7,26071973	3,47415729	0,00109095	0,07122422
TC0100012690.hg.1	KLHL21	9903	-0,31647275	7,48111765	-3,47296769	0,00109482	0,07122516
TC1100007762.hg.1	OOSP2	219990	-0,34213404	4,95994634	-3,47035068	0,00110338	0,07153008

TC1800007066.hg.1	MAPRE2	10982	1,00465915	8,4653701	3,46726604	0,00111355	0,07163786
TC0900009333.hg.1	FAM157B	100132403	-0,68436605	8,52122457	-3,46639765	0,00111643	0,07163786
TC1000009006.hg.1	PNLIPRP1	5407	-0,28634168	4,60197361	-3,46553165	0,00111931	0,07163786
TC1100008521.hg.1	B3GNT6	192134	-0,30776813	6,77999675	-3,46515771	0,00112055	0,07163786
TC0200013079.hg.1	DUSP11	8446	0,50011168	9,77333946	3,46009525	0,00113754	0,07228129
TC1100008041.hg.1	CTSW	1521	0,80855423	7,5353852	3,4596606	0,00113901	0,07228129
TC0900010792.hg.1	NOL8	55035	0,59775432	7,24974433	3,45819824	0,00114397	0,07228129
TC0500010480.hg.1	PRLR	5618	-0,44984542	4,80361015	-3,45615896	0,00115092	0,07228129
TC1200008748.hg.1	PWP1	11137	0,5295387	8,53135272	3,45575651	0,00115229	0,07228129
TC1200010407.hg.1	KIF21A	55605	0,99444645	5,74772682	3,45523074	0,00115409	0,07228129
TC2200009274.hg.1	APOBEC3G	60489	1,19499259	7,31106823	3,45055775	0,00117021	0,07304307
TC1900011679.hg.1	CYP4F3	4051	-0,25395892	5,84345995	-3,44305171	0,00119654	0,07422607
TC2100006903.hg.1	SOD1	6647	0,41292426	10,7966086	3,44286825	0,0011972	0,07422607
TC1100008174.hg.1	ALDH3B1	221	-0,48517617	6,77121623	-3,44019664	0,00120671	0,07451719
TC1700009613.hg.1	ASGR2	433	-0,41362233	6,45123963	-3,43928933	0,00120996	0,07451719
TC2200008799.hg.1	ST13	6767	0,59891872	7,77330739	3,43808763	0,00121427	0,07453444
TC0900011992.hg.1	AGPAT2	10555	-0,37738707	7,96152515	-3,42988791	0,0012441	0,0760082
TC0700012276.hg.1	DOCK4	9732	-0,36844995	3,87821369	-3,42923439	0,00124651	0,0760082
TC0400006742.hg.1	SH3TC1	54436	-0,33505043	8,383198	-3,42339128	0,00126823	0,0767534
TC1600006577.hg.1	RNF151	146310	-0,39755155	6,49914317	-3,42195912	0,00127361	0,0767534
TC0800012132.hg.1	ZC3H3	23144	-0,34386644	6,61268299	-3,42136928	0,00127583	0,0767534
TC0400006579.hg.1	ADD1	118	0,69348898	8,49431117	3,42065931	0,00127851	0,0767534
TC1100012685.hg.1	VSIG2	23584	-0,31225212	6,65732884	-3,42039672	0,0012795	0,0767534
TC0100007206.hg.1	CDA	978	-0,6491069	5,13439311	-3,41837695	0,00128716	0,07696278
TC1200011711.hg.1	GNPTAB	79158	0,77191932	8,93289776	3,41583863	0,00129684	0,07729163
TC1000009149.hg.1	PLEKHA1	59338	1,09260994	7,96064401	3,41327887	0,00130668	0,0776274
TC0100009524.hg.1	VANGL1	81839	0,4392406	5,69051172	3,4092359	0,00132235	0,07818544
TC1200012587.hg.1	GPR162	27239	-0,4700118	6,17597227	-3,40695485	0,00133128	0,07818544
TC0200013700.hg.1	MFSD9	84804	0,64862563	7,40636038	3,40654646	0,00133288	0,07818544
TC1900007917.hg.1	ZNF146	7705	0,7071078	7,26292658	3,40651776	0,001333	0,07818544
TC1100011113.hg.1	SLC22A8	9376	-0,29941553	4,73015784	-3,40502352	0,00133888	0,07827062
TC0800007027.hg.1	SLC25A37	51312	-0,68536788	6,85757961	-3,40400122	0,00134292	0,07827062
TC0600013160.hg.1	SAMD3	154075	1,69685757	6,91150403	3,40212487	0,00135037	0,07845708
TC0100017500.hg.1	TMEM63A	9725	0,56029955	9,68153957	3,40004586	0,00135866	0,07852615
TC1700011566.hg.1	FAM20A	54757	-0,52805279	4,92429803	-3,39969784	0,00136006	0,07852615
TC2100008424.hg.1	SLC19A1	6573	-0,66002911	5,90451702	-3,39581664	0,00137569	0,07877718

TC1100006580.hg.1	KCNQ1	3784	-0,40837945	7,49933968	-3,39573027	0,00137604	0,07877718
TC1700008135.hg.1	TBX21	30009	0,57568246	6,85317063	3,39460643	0,0013806	0,07877718
TC0800010685.hg.1	MYBL1	4603	1,74493375	6,56210236	3,39439439	0,00138146	0,07877718
TC0100013223.hg.1	RAP1GAP	5909	-0,28451809	4,48591019	-3,39096787	0,00139546	0,07924876
TC1300009273.hg.1	COMMD6	170622	0,43925278	10,4851306	3,39027417	0,00139831	0,07924876
TC0200016640.hg.1	HS1BP3	64342	-0,30571027	5,78959912	-3,38666393	0,00141323	0,0798496
TC1400010575.hg.1	BCL2L2	599	-0,32168389	7,92376619	-3,38428169	0,00142316	0,08016551
TC0600009459.hg.1	ENPP1	5167	0,48205543	5,1409348	3,38241585	0,00143098	0,08036121
TC0400010615.hg.1	TXK	7294	1,39833899	7,88830942	3,38137222	0,00143538	0,0803637
TC1700006773.hg.1	TMEM88	92162	-0,44014062	7,38598475	-3,37961192	0,00144282	0,08046065
TC1600010966.hg.1	CDYL2	124359	-0,42758229	6,78006867	-3,37674434	0,00145502	0,08046065
TC1600010599.hg.1	NAE1	8883	0,64470305	6,87924356	3,37370704	0,00146804	0,08046065
TC0200015246.hg.1	STAT4	6775	1,59616737	7,28714355	3,37352676	0,00146882	0,08046065
TC0600010021.hg.1	SYTL3	94120	0,81337581	7,12125053	3,37280753	0,00147192	0,08046065
TC0200007702.hg.1	PAPOLG	64895	0,5844694	8,38676638	3,37247604	0,00147336	0,08046065
TC0600014258.hg.1	HLA-B	3106	0,3188768	11,5875289	3,37234467	0,00147392	0,08046065
TC0200016441.hg.1	TMEM247	388946	-0,39164295	5,95472674	-3,37116428	0,00147904	0,08046065
TC1500010842.hg.1	GOLGA8R	101059918	0,63306389	6,16632376	3,36901135	0,0014884	0,08046065
TC0200015838.hg.1	FARSB	10056	0,49774325	7,79223494	3,36887554	0,001489	0,08046065
TC1500008933.hg.1	GOLGA8O	728047	0,80990914	7,30734058	3,36866912	0,0014899	0,08046065
TC0200013894.hg.1	ANAPC1	64682	0,52274014	7,05592365	3,36832558	0,0014914	0,08046065
TC0400009450.hg.1	WWC2	80014	-0,29236808	4,72500336	-3,36779482	0,00149372	0,08046065
TC1600011417.hg.1	NFATC3	4775	0,97946291	9,3864641	3,36387087	0,001511	0,08085523
TC1900008910.hg.1	ZNF628	89887	-0,31724485	6,83837439	-3,36343878	0,00151291	0,08085523
TC0X00011274.hg.1	TCEANC	170082	0,52850832	6,21317018	3,363154	0,00151418	0,08085523
TC0100009858.hg.1	PPIAL4C	653598	0,36319089	8,32535836	3,35949315	0,0015305	0,08138502
TC1000007598.hg.1	AGAP6	414189	0,4041223	9,71751986	3,35882505	0,0015335	0,08138502
TC1600011520.hg.1	SEPTIN1	1731	0,6775927	8,21312642	3,35753911	0,00153928	0,08138502
TC2000007269.hg.1	TLDC2	140711	-0,33713605	5,72404406	-3,35576025	0,00154731	0,08138502
TC0300010561.hg.1	SLC4A7	9497	0,56592794	9,10180612	3,35510168	0,0015503	0,08138502
TC1900011877.hg.1	ZNF625	90589	0,3539827	6,94464353	3,35463465	0,00155242	0,08138502
TC1600009165.hg.1	MMP25-AS1	100507419	0,76561074	6,5286004	3,35375943	0,0015564	0,08138502
TC1900012038.hg.1	LILRA6	79168	-0,60238909	7,21305383	-3,35311484	0,00155934	0,08138502
TC0300009271.hg.1	GMPS	8833	0,59001096	7,72364143	3,34691376	0,00158787	0,08264066
TC0100015588.hg.1	RNF115	27246	0,50225323	8,34227923	3,3443287	0,00159991	0,08303336
TC1000011376.hg.1	CH25H	9023	-0,76717631	4,47243724	-3,34208444	0,00161043	0,08306185

TC2200008874.hg.1	NFAM1	150372	-0,54218079	6,70893212	-3,34193315	0,00161114	0,08306185
TC0100013749.hg.1	RSPO1	284654	-0,31332769	7,06168455	-3,3405699	0,00161757	0,08306185
TC0100017079.hg.1	SLC41A1	254428	0,55865847	8,34994373	3,33892249	0,00162537	0,08306185
TC0300010566.hg.1	EOMES	8320	1,03618166	7,69324309	3,33860232	0,00162689	0,08306185
TC1100008381.hg.1	P2RY6	5031	-0,37522674	6,44757061	-3,33778493	0,00163077	0,08306185
TC0100015417.hg.1	VTCN1	79679	-0,26325114	4,82115436	-3,3375426	0,00163193	0,08306185
TC1700012177.hg.1	LOC339166	339166	-0,27918626	4,7909577	-3,33467828	0,00164562	0,08352885
TC1200011385.hg.1	LIN7A	8825	-0,9859852	4,91767485	-3,33247032	0,00165625	0,0838382
TC1900009368.hg.1	TICAM1	148022	-0,33687247	6,28443588	-3,32628152	0,0016864	0,08491038
TC0100016659.hg.1	APOBEC4	403314	-0,20048389	3,51771889	-3,32530767	0,0016912	0,08491038
TC1700012330.hg.1	OR3A2	4995	-0,28786798	5,46146895	-3,32447287	0,00169531	0,08491038
TC0100015171.hg.1	HENMT1	113802	0,77219058	6,82952228	3,32377427	0,00169877	0,08491038
TC1400007029.hg.1	C14orf28	122525	0,31061159	5,47941689	3,32325923	0,00170132	0,08491038
TC1200006738.hg.1	KLRG1	10219	1,41095007	7,22825441	3,32251442	0,00170501	0,08491038
TC0200008167.hg.1	REG3G	130120	-0,32539955	5,92154891	-3,32102849	0,0017124	0,08504929
TC0100007542.hg.1	SMPDL3B	27293	-0,3697739	6,34280085	-3,31923832	0,00172135	0,08518853
TC0500009422.hg.1	NPM1	4869	0,55610805	8,92345372	3,31862434	0,00172443	0,08518853
TC1800008474.hg.1	SLC39A6	25800	0,56661939	8,14718818	3,31580224	0,00173865	0,08524284
TC0700011770.hg.1	KRIT1	889	0,96717528	9,00643577	3,31553023	0,00174002	0,08524284
TC1000011585.hg.1	GOT1	2805	0,71731966	7,57544541	3,31385077	0,00174854	0,08524284
TC2100008491.hg.1	GRIK1-AS2	100379661	-0,51009144	5,37532543	-3,31255126	0,00175516	0,08524284
TC0600011439.hg.1	NCR3	259197	0,77199786	8,72163452	3,31241506	0,00175586	0,08524284
TC0100014638.hg.1	PIGK	10026	0,94155328	6,22669289	3,31174172	0,0017593	0,08524284
TC0100009555.hg.1	CD2	914	1,020573	5,2665569	3,31141135	0,00176099	0,08524284
TC0100007574.hg.1	EPB41	2035	0,43028834	10,6625923	3,31021396	0,00176713	0,08524284
TC1300007228.hg.1	WDFY2	115825	-0,34477655	8,10356651	-3,30991826	0,00176865	0,08524284
TC1200011811.hg.1	MTERF2	80298	0,61169809	6,98367973	3,30877897	0,00177451	0,08524284
TC1100011552.hg.1	CLPB	81570	-0,53768306	8,62785774	-3,30843681	0,00177628	0,08524284
TC1900007795.hg.1	LSM14A	26065	0,41700392	7,5199888	3,30505022	0,00179384	0,08586257
TC0300013684.hg.1	TFRC	7037	-0,81307676	5,92036331	-3,30257094	0,0018068	0,08609745
TC0200016224.hg.1	PER2	8864	-0,40899417	5,10226256	-3,30232953	0,00180807	0,08609745
TC0X00010211.hg.1	CHM	1121	0,41893588	6,54761246	3,29887593	0,00182628	0,08659372
TC2200009236.hg.1	GGT1	2678	-0,31075185	7,12384649	-3,29857757	0,00182786	0,08659372
TC1200009416.hg.1	RAN	5901	0,51139765	10,2544253	3,29738548	0,00183419	0,08667147
TC1700012257.hg.1	KRTAP9-3	83900	-0,39161703	6,0937088	-3,29204202	0,00186283	0,08773642
TC0100007105.hg.1	PADI4	23569	-0,92859148	6,61751437	-3,29064745	0,00187038	0,08773642

TC1600010792.hg.1	PHLPP2	23035	0,74560758	6,42900158	3,29022266	0,00187268	0,08773642
TC0800009244.hg.1	MROH1	727957	-0,30450031	7,43948686	-3,28945338	0,00187686	0,08773642
TC1100011723.hg.1	RSF1	51773	0,50362909	9,07732165	3,28878948	0,00188047	0,08773642
TC0100011461.hg.1	UTP25	27042	0,42160257	6,55130767	3,28742518	0,00188792	0,08786196
TC1100008999.hg.1	ZC3H12C	85463	-0,37874125	4,25305076	-3,28411758	0,00190609	0,08848463
TC1400010612.hg.1	PCNX4	64430	0,84354955	7,96856114	3,28178307	0,00191901	0,08872156
TC1100009068.hg.1	NCAM1	4684	1,81538909	6,7678322	3,28046372	0,00192635	0,08872156
TC1100012358.hg.1	USP28	57646	1,13278901	6,49527535	3,27997804	0,00192906	0,08872156
TC0300008320.hg.1	CD96	10225	1,47809149	9,80884116	3,27959108	0,00193122	0,08872156
TC0900009470.hg.1	ERMP1	79956	0,62704949	5,73716992	3,27682668	0,00194672	0,08872156
TC1800008285.hg.1	NPC1	4864	0,80473218	7,3362224	3,27597945	0,0019515	0,08872156
TC1900012040.hg.1	LILRA5	353514	-0,50699157	7,81318271	-3,27529926	0,00195534	0,08872156
TC2000009968.hg.1	SIRPB2	284759	-0,45275185	7,18555566	-3,27529314	0,00195537	0,08872156
TC2000009918.hg.1	BPI	671	-0,47426669	6,67502761	-3,27343878	0,00196588	0,08872156
TC0400008620.hg.1	AFG2A	166378	0,58742474	7,25378847	3,27308336	0,0019679	0,08872156
TC0100009939.hg.1	GABPB2	126626	0,62996195	9,66932187	3,27300104	0,00196837	0,08872156
TC1700011435.hg.1	ICAM2	3384	0,52678026	7,64325483	3,27292301	0,00196882	0,08872156
TC0X00009644.hg.1	KCND1	3750	-0,30200575	5,15316897	-3,27156955	0,00197653	0,08885063
TC0500013384.hg.1	PPP2R2B	5521	1,43100205	5,58778467	3,27073574	0,0019813	0,08885063
TC1600009545.hg.1	ABCC6	368	-0,3429675	7,207369	-3,26922171	0,00198998	0,08902399
TC1200006786.hg.1	TMEM52B	120939	-0,25975844	5,22139895	-3,26722324	0,0020015	0,08932297
TC0700013426.hg.1	PVRIG	79037	0,54918516	8,93267554	3,26533685	0,00201243	0,08959432
TC0800007681.hg.1	TGS1	96764	0,45639759	8,18018436	3,26414104	0,00201939	0,08968797
TC0600008509.hg.1	KCNQ5	56479	0,97408946	6,63633129	3,26198164	0,00203201	0,0898551
TC0100012687.hg.1	NOL9	79707	0,562357	7,95618441	3,26166248	0,00203388	0,0898551
TC1200010198.hg.1	INTS13	55726	1,02640359	6,75235727	3,2610053	0,00203774	0,0898551
TC0100011269.hg.1	LINC00260	84719	0,77662589	6,71171859	3,25735525	0,0020593	0,09008686
TC0200008663.hg.1	IL1R2	7850	-0,85359081	6,05328018	-3,2572442	0,00205996	0,09008686
TC0100018180.hg.1	CLCNKA	1187	-0,36240958	5,51050245	-3,25679145	0,00206265	0,09008686
TC0500012023.hg.1	KIF3A	11127	0,48127058	5,24722681	3,25665056	0,00206349	0,09008686
TC0300009789.hg.1	ST6GAL1	6480	0,55441576	9,26418874	3,25599791	0,00206738	0,09008686
TC1700010452.hg.1	LYZL6	57151	-0,3019352	4,30291826	-3,25370193	0,0020811	0,09047152
TC1100008006.hg.1	SLC22A20P	440044	-0,28771248	4,92150478	-3,25244495	0,00208865	0,09058659
TC0300012345.hg.1	LINC01565	23434	-0,24654893	5,96798283	-3,24657996	0,00212422	0,09180428
TC1200009253.hg.1	ATP6V0A2	23545	0,48815503	8,50317526	3,2461807	0,00212666	0,09180428
TC1200007170.hg.1	CCDC91	55297	0,40102402	9,51924036	3,24313327	0,00214539	0,09239687

TC1700008498.hg.1	PPM1D	8493	0,40229295	6,12498661	3,24001356	0,00216473	0,09301273
TC1300009765.hg.1	IRS2	8660	-0,84242676	6,01076935	-3,23844	0,00217454	0,09321765
TC1700007967.hg.1	CD300LG	146894	-0,29779577	6,66017883	-3,23379761	0,00220374	0,09383673
TC0200013113.hg.1	LOXL3	84695	-0,41838293	6,60517389	-3,23323342	0,00220732	0,09383673
TC0900011713.hg.1	FNBP1	23048	0,35931384	9,6352963	3,23302517	0,00220864	0,09383673
TC0100014243.hg.1	NDC1	55706	0,69383287	7,17352551	3,23292079	0,0022093	0,09383673
TC0100014788.hg.1	ZNHIT6	54680	0,7491798	6,39086542	3,23102884	0,00222133	0,0941315
TC1800009277.hg.1	PIAS2	9063	0,51797659	7,99475813	3,22958388	0,00223057	0,09430646
TC1700007599.hg.1	AP2B1	163	0,59377126	8,14531537	3,22831447	0,00223871	0,09442841
TC2200007273.hg.1	CSF2RB	1439	-0,4794794	8,34884215	-3,22754253	0,00224367	0,09442841
TC1500010672.hg.1	TARS3	123283	0,59900291	5,58146609	3,22649312	0,00225044	0,09449789
TC1700007415.hg.1	CRYBA1	1411	-0,30798888	4,69052953	-3,22459017	0,00226276	0,09463928
TC0X00009637.hg.1	PIM2	11040	0,66798472	10,1224633	3,22295345	0,0022734	0,09463928
TC1800007696.hg.1	TSHZ1	10194	0,37761967	6,5468217	3,22282297	0,00227425	0,09463928
TC2200006833.hg.1	BCR	613	0,54142823	6,11055783	3,22281647	0,00227429	0,09463928
TC0300010427.hg.1	RFTN1	23180	0,4709033	8,92398084	3,22133164	0,002284	0,09472374
TC0100007676.hg.1	LCK	3932	0,78771399	6,81561806	3,22045812	0,00228972	0,09472374
TC2100007467.hg.1	DIP2A	23181	0,64236614	6,75809671	3,22015617	0,0022917	0,09472374
TC1100008505.hg.1	EMSY	56946	0,632578	9,1249379	3,21912508	0,00229849	0,09479199
TC1900008967.hg.1	ZNF470	388566	0,51712556	5,87186299	3,21828966	0,00230399	0,09480302
TC1700010856.hg.1	C1QL1	10882	-0,36787569	7,14957017	-3,21752981	0,00230902	0,09480302
TC0300011259.hg.1	ARHGEF3	50650	0,69880244	7,49075276	3,21415643	0,00233143	0,09551116
TC0300009131.hg.1	HPS3	84343	0,4960783	8,78159272	3,21024101	0,00235771	0,09578431
TC0X00011332.hg.1	PLXNB3	5365	-0,36740874	6,1531645	-3,21013936	0,00235839	0,09578431
TC1200012842.hg.1	ANAPC7	51434	0,84505935	8,40313982	3,21008748	0,00235874	0,09578431
TC1100011367.hg.1	UNC93B1	81622	-0,68426212	8,08080428	-3,2100738	0,00235884	0,09578431
TC0300011105.hg.1	RASSF1	11186	0,38421292	5,96103641	3,2090022	0,00236608	0,09586774
TC1000012428.hg.1	AKR1C3	8644	0,93142015	5,93369878	3,2069025	0,00238033	0,09601709
TC2200008103.hg.1	YPEL1	29799	0,5734142	6,21335401	3,20673464	0,00238148	0,09601709
TC1700011768.hg.1	GALK1	2584	-0,29728832	8,52902623	-3,20590564	0,00238713	0,09601709
TC0200007717.hg.1	AHSA2P	130872	0,90142498	9,10768027	3,20540428	0,00239055	0,09601709
TC0500007154.hg.1	SLC1A3	6507	-0,55064985	5,98214807	-3,20351864	0,00240348	0,09632668
TC1800008730.hg.1	MBD2	8932	-0,33243886	9,89376568	-3,20146718	0,00241761	0,09640977
TC1700011808.hg.1	ST6GALNAC2	10610	-0,38444147	7,17871258	-3,20135881	0,00241836	0,09640977
TC0200007486.hg.1	TTC7A	57217	-0,54269467	7,70312057	-3,20094717	0,0024212	0,09640977
TC0700010247.hg.1	RPA3	6119	0,74048123	7,95367904	3,19854937	0,00243784	0,09677011

TC1100009207.hg.1	KMT2A	4297	0,38945583	8,68818361	3,19790004	0,00244237	0,09677011
TC0900010580.hg.1	GKAP1	80318	0,90007483	5,7851529	3,19722175	0,0024471	0,09677011
TC0400008007.hg.1	GPAT3	84803	-0,38115906	4,5579231	-3,19663575	0,0024512	0,09677011
TC1900011464.hg.1	TMEM150B	284417	-0,65063276	5,66377588	-3,19453357	0,00246596	0,09714501
TC1200009916.hg.1	TAS2R9	50835	-0,18003698	3,63858783	-3,19227615	0,00248189	0,09756482
TC1100008477.hg.1	DGAT2	84649	-0,49939621	6,06643574	-3,19131253	0,00248873	0,0976257
TC1600008003.hg.1	ADGRG5	221188	0,71275888	8,34782642	3,18963569	0,00250066	0,09769579
TC0100010625.hg.1	FASLG	356	0,35867369	5,18013049	3,18957538	0,00250109	0,09769579
TC0500009116.hg.1	GM2A	2760	-0,48528097	7,34817988	-3,18430617	0,00253894	0,09860751
TC1700011263.hg.1	TEX14	56155	-0,27389287	4,66358472	-3,18027126	0,00256829	0,09860751
TC0900012229.hg.1	EXOSC3	51010	0,56279624	5,57950309	3,18019537	0,00256885	0,09860751
TC0200012809.hg.1	PELI1	57162	-1,08625077	7,53128858	-3,18002886	0,00257007	0,09860751
TC2100007359.hg.1	TRPM2	7226	-0,45731477	6,15543499	-3,17923562	0,00257588	0,09860751
TC1700006786.hg.1	ALOX15B	247	-0,46177886	5,38352621	-3,17918445	0,00257625	0,09860751
TC1700010587.hg.1	IKZF3	22806	0,56146071	8,35119619	3,17910359	0,00257685	0,09860751
TC2000007386.hg.1	PLCG1	5335	1,14851246	6,80494232	3,17864115	0,00258024	0,09860751
TC1400007445.hg.1	PLEKHG3	26030	0,62610854	6,6515033	3,17859964	0,00258054	0,09860751
TC1800006583.hg.1	LRRC30	339291	-0,31207974	5,15484076	-3,17803037	0,00258473	0,09860751
TC1000012498.hg.1	GSTO2	119391	-0,18493447	5,364444	-3,17774887	0,0025868	0,09860751
TC1700006740.hg.1	ACAP1	9744	0,39709399	10,3130889	3,17752188	0,00258847	0,09860751
TC0100015596.hg.1	LIX1L	128077	0,77304439	7,63703829	3,17398995	0,00261462	0,09923605
TC0800007949.hg.1	TERF1	7013	0,5376279	7,81266015	3,17384267	0,00261572	0,09923605
TC1000009666.hg.1	CALML5	51806	-0,25797536	7,10226563	-3,17288898	0,00262282	0,09930171
TC0300011052.hg.1	LAMB2	3913	-0,30898861	7,51757358	-3,16991622	0,00264509	0,0996896
TC0700012522.hg.1	PRRT4	401399	-0,33428031	6,79194677	-3,16988383	0,00264533	0,0996896
TC0100012463.hg.1	ACAP3	116983	-0,30524169	7,62412707	-3,16902812	0,00265177	0,0996896
TC0600009488.hg.1	RPS12	6206	0,2021478	12,1578936	3,16864667	0,00265465	0,0996896
TC1100012474.hg.1	MPZL2	10205	-0,48067848	6,33995869	-3,1665786	0,0026703	0,10007379
TC0600009446.hg.1	AKAP7	9465	0,38057175	5,07608259	3,16372399	0,00269204	0,10038201
TC0100010334.hg.1	UFC1	51506	0,38213842	9,83765888	3,16262619	0,00270044	0,10038201
TC0X00007134.hg.1	USP11	8237	0,53377671	8,16600287	3,16223312	0,00270346	0,10038201
TC1400009732.hg.1	ERG28	11161	0,55638059	8,71346921	3,16216104	0,00270401	0,10038201
TC1700011074.hg.1	DLX3	1747	-0,29625153	6,22886782	-3,16194299	0,00270569	0,10038201
TC1300009933.hg.1	RASA3	22821	0,53662604	9,47427065	3,15900732	0,00272833	0,10070764
TC1200011301.hg.1	KRR1	11103	0,3425546	7,35260399	3,15835631	0,00273337	0,10070764
TC0200010516.hg.1	CYP20A1	57404	0,74121761	6,82460379	3,15751192	0,00273993	0,10070764

TC1900008931.hg.1	RFPL4A	342931	-0,43270511	4,52198718	-3,15700142	0,0027439	0,10070764
TC1300007256.hg.1	OLFM4	10562	-0,35184201	4,90398417	-3,1564958	0,00274784	0,10070764
TC0900011254.hg.1	CDC26	246184	0,34986466	9,57527204	3,1555229	0,00275543	0,10070764
TC0100015243.hg.1	KCNA10	3744	-0,34719859	7,6373524	-3,15522309	0,00275777	0,10070764
TC0300010714.hg.1	TRANK1	9881	0,84115699	7,7707297	3,15518527	0,00275807	0,10070764
TC1000008276.hg.1	CCSER2	54462	0,73266232	9,66517358	3,15339852	0,00277208	0,10091109
TC0400007379.hg.1	GRXCR1	389207	-0,24967108	4,22710781	-3,15308226	0,00277456	0,10091109
TC1500010878.hg.1	INTS14	81556	0,70481847	6,84116696	3,15189991	0,00278388	0,10105095
TC1100007110.hg.1	FIBIN	387758	-0,2819069	5,81346799	-3,14977092	0,00280073	0,10132327
TC0100007920.hg.1	PPIE	10450	0,5753023	6,72859918	3,14935829	0,002804	0,10132327
TC0X00008344.hg.1	SH2D1A	4068	1,25512311	7,0545341	3,14857007	0,00281027	0,10132327
TC0100015660.hg.1	PPIAL4G	644591	0,27810156	7,34948053	3,14818756	0,00281332	0,10132327
TC0600007402.hg.1	ZKSCAN8	7745	0,61546972	7,75259294	3,14663379	0,00282573	0,10157215
TC1900006464.hg.1	TPGS1	91978	-0,27126956	7,17319705	-3,14429631	0,00284449	0,10204808
TC1900011797.hg.1	LILRA2	11027	-0,42853032	8,7896441	-3,14290228	0,00285573	0,10223049
TC1700010630.hg.1	CCR7	1236	0,88347919	9,77344517	3,14229592	0,00286064	0,10223049
TC0100012816.hg.1	CLSTN1	22883	0,45101395	8,3269839	3,14049972	0,00287521	0,10255301
TC0500007314.hg.1	MRPS30	10884	0,55106463	8,67553061	3,13906566	0,0028869	0,10277147
TC1700009093.hg.1	CBX2	84733	-0,38752539	7,5969775	-3,13415433	0,00292727	0,1038015
TC0600013588.hg.1	SYNE1	23345	0,89073949	6,95331273	3,13330017	0,00293434	0,1038015
TC0200008724.hg.1	MRPS9	64965	0,5374968	7,35946741	3,13289819	0,00293768	0,1038015
TC1900011312.hg.1	ZNF611	81856	0,43394528	9,58225039	3,13282199	0,00293831	0,1038015
TC0400011018.hg.1	CXCL2	2920	-0,32983547	7,86634669	-3,13162852	0,00294823	0,10392103
TC1500010921.hg.1	MRPL46	26589	0,57957603	7,51956301	3,13087141	0,00295454	0,10392103
TC0800011881.hg.1	NDRG1	10397	-0,32759472	7,57892222	-3,13016543	0,00296044	0,10392103
TC0800012300.hg.1	FNTA	2339	0,57688673	5,06396971	3,12971724	0,00296419	0,10392103
TC1600007827.hg.1	NOD2	64127	-0,4892046	7,4931751	-3,12771209	0,00298102	0,10424104
TC1100013045.hg.1	SIPA1	6494	0,7055225	7,7030817	3,12675634	0,00298907	0,10424104
TC0500012286.hg.1	ARAP3	64411	-0,3295171	7,25553256	-3,12631598	0,00299279	0,10424104
TC2100007996.hg.1	TMEM50B	757	0,8704541	8,05145162	3,12594966	0,00299589	0,10424104
TC0100013882.hg.1	FOXJ3	22887	0,43734963	9,49464658	3,120091	0,00304581	0,10535003
TC2000007184.hg.1	ITCH	83737	0,55897097	8,50852494	3,11991943	0,00304728	0,10535003
TC0700013529.hg.1	HOXA3	3200	-0,38200327	7,11764536	-3,11828297	0,00306137	0,10535003
TC2100006922.hg.1	EVA1C	59271	0,60617818	7,0851528	3,11799138	0,00306389	0,10535003
TC0200015399.hg.1	PPIL3	53938	0,60613329	7,37123211	3,11767823	0,00306659	0,10535003
TC0200009539.hg.1	ARHGAP15	55843	0,64692299	9,74080646	3,11718402	0,00307087	0,10535003

TC0100006784.hg.1	TMEM201	199953	-0,34775136	7,30609875	-3,11684627	0,00307379	0,10535003
TC1700007014.hg.1	UBB	7314	0,26613334	12,2256258	3,1162972	0,00307855	0,10535003
TC0700008762.hg.1	CBLL1	79872	0,5835653	7,87328519	3,11600977	0,00308105	0,10535003
TC0700013485.hg.1	LOC155060	155060	-0,36758653	7,56726497	-3,11394225	0,00309904	0,10535003
TC1000007564.hg.1	MAPK8	5599	0,7734017	8,98143917	3,11392804	0,00309917	0,10535003
TC0200015082.hg.1	FKBP7	51661	0,446173	4,8992294	3,11385202	0,00309983	0,10535003
TC2200009363.hg.1	CIMAP1B	440836	-0,39823564	7,1694255	-3,1136167	0,00310188	0,10535003
TC0600014282.hg.1	RPS10	6204	0,24783102	11,5042182	3,11268354	0,00311005	0,10543342
TC1900008419.hg.1	C5AR2	27202	-0,42610656	5,49088189	-3,10970286	0,00313625	0,10602925
TC0700013231.hg.1	MNX1	3110	-0,44910763	6,18728007	-3,10767513	0,0031542	0,10602925
TC1200009580.hg.1	FBXL14	144699	0,49719832	7,98976137	3,10761185	0,00315476	0,10602925
TC1900007456.hg.1	ZNF253	56242	0,53312982	8,74095383	3,10747195	0,003156	0,10602925
TC1900007207.hg.1	OR1I1	126370	-0,23718119	4,03619099	-3,10632126	0,00316624	0,10602925
TC1700006643.hg.1	GLTPD2	388323	-0,33414497	5,96348635	-3,10590957	0,0031699	0,10602925
TC1900008544.hg.1	RCN3	57333	-0,37986693	6,88137532	-3,10552382	0,00317335	0,10602925
TC1400006549.hg.1	RNASE8	122665	-0,27158168	4,38072459	-3,1055029	0,00317353	0,10602925
TC1900011916.hg.1	ZNF626	199777	0,65406328	7,94101448	3,10389897	0,00318788	0,1063163
TC1100012545.hg.1	TRIM29	23650	-0,28757308	5,62117211	-3,10262332	0,00319933	0,10648536
TC0300012818.hg.1	IGSF10	285313	-0,31673189	3,98956382	-3,10100392	0,00321393	0,10648536
TC0100013339.hg.1	RUNX3	864	0,70686214	9,64445438	3,10063142	0,00321729	0,10648536
TC0400010484.hg.1	PDS5A	23244	0,45044986	10,4263453	3,09970475	0,00322568	0,10648536
TC0800009619.hg.1	CTSB	1508	-0,83506735	6,72430835	-3,09950421	0,0032275	0,10648536
TC0X00008405.hg.1	RBMX2	51634	0,46753755	7,98613103	3,09924047	0,00322989	0,10648536
TC0X00007655.hg.1	SLC16A2	6567	-0,28829009	5,24282035	-3,09886602	0,00323329	0,10648536
TC1700012412.hg.1	KRT33B	3884	-0,28270499	5,11441421	-3,09815503	0,00323976	0,10650839
TC1200012574.hg.1	TULP3	7289	0,54986908	7,54274934	3,09523688	0,00326641	0,10719406
TC0900011256.hg.1	RNF183	138065	-0,32928068	7,42078276	-3,09446048	0,00327354	0,10723748
TC1500008328.hg.1	BLM	641	0,65201554	6,87475136	3,09005974	0,00331422	0,10790587
TC1700012289.hg.1	CA4	762	-0,26558588	5,02168663	-3,08952721	0,00331917	0,10790587
TC1100012984.hg.1	NAT10	55226	0,51593678	7,12605587	3,08866833	0,00332718	0,10790587
TC1100009234.hg.1	TRAPPC4	51399	0,48329438	8,37562089	3,08716182	0,00334126	0,10790587
TC0700009072.hg.1	IRF5	3663	-0,27243714	6,82712071	-3,08663494	0,0033462	0,10790587
TC1400008637.hg.1	SALL2	6297	-0,25694576	5,00188996	-3,08602277	0,00335195	0,10790587
TC1400007161.hg.1	PTGDR	5729	1,17891064	6,97246386	3,08510945	0,00336054	0,10790587
TC0300009337.hg.1	SMC4	10051	0,96279838	6,61618825	3,08484022	0,00336308	0,10790587
TC1200006909.hg.1	EMP1	2012	-0,46201526	4,98571451	-3,08422288	0,0033689	0,10790587

TC0300008999.hg.1	ZBTB38	253461	0,95092899	7,88496994	3,08419731	0,00336914	0,10790587
TC0600014105.hg.1	C2	717	-0,40671148	7,01085223	-3,08387292	0,0033722	0,10790587
TC0700013538.hg.1	TARP	445347	1,90193715	7,68667309	3,08363236	0,00337448	0,10790587
TC0500013118.hg.1	OR2V1	26693	-0,27126283	4,99386344	-3,08257884	0,00338445	0,10790587
TC1900007819.hg.1	ZNF302	55900	0,55822993	7,72253419	3,08219313	0,00338811	0,10790587
TC1200008734.hg.1	RIC8B	55188	0,40646326	7,12107325	3,08183917	0,00339147	0,10790587
TC0100018282.hg.1	POLR3GL	84265	0,344186	6,94847737	3,08135308	0,00339609	0,10790587
TC1100011295.hg.1	CCDC87	55231	-0,2980032	5,70522116	-3,08070142	0,0034023	0,10790587
TC0400008892.hg.1	HHIP	64399	-0,26040452	4,40946678	-3,07950712	0,0034137	0,10790587
TC1100007518.hg.1	OR4S1	256148	-0,2485516	5,89602212	-3,07926258	0,00341603	0,10790587
TC1600009868.hg.1	SULT1A2	6799	-0,38171914	7,08567568	-3,07920525	0,00341658	0,10790587
TC1800006715.hg.1	IMPA2	3613	-0,36328349	6,86906323	-3,07920425	0,00341659	0,10790587
TC1100006444.hg.1	PGGHG	80162	0,45434611	8,53790778	3,07793457	0,00342876	0,10810531
TC1700009640.hg.1	ZBTB4	57659	0,45438508	7,75609045	3,07640556	0,00344346	0,10819869
TC0400010500.hg.1	RBM47	54502	-0,71397821	6,5666664	-3,07582182	0,00344909	0,10819869
TC0400012252.hg.1	PPID	5481	0,49847779	6,02091163	3,07580151	0,00344929	0,10819869
TC1700009402.hg.1	SMYD4	114826	0,74487414	6,70412622	3,07501332	0,0034569	0,10825378
TC1000012567.hg.1	AGAP5	729092	0,3143238	9,2561102	3,07416783	0,00346509	0,10832654
TC0100006910.hg.1	PRAMEF2	65122	-0,28774398	4,47632063	-3,07268718	0,00347947	0,10859235
TC0200012307.hg.1	SOS1	6654	0,63189275	6,19122859	3,07189439	0,00348719	0,10864984
TC0200008291.hg.1	KDM3A	55818	0,57939687	5,96063703	3,06952334	0,00351039	0,10875171
TC1900012017.hg.1	CLDND2	125875	0,37217488	7,81128284	3,06913896	0,00351416	0,10875171
TC0700012871.hg.1	OR9A2	135924	-0,32199346	5,0571011	-3,0682196	0,0035232	0,10875171
TC0700013327.hg.1	CHST12	55501	0,67075675	8,26422442	3,0672403	0,00353285	0,10875171
TC1100006860.hg.1	USP47	55031	0,56069103	7,20082942	3,06700482	0,00353518	0,10875171
TC0500009409.hg.1	GABRP	2568	-0,28784265	6,74436553	-3,06637707	0,00354138	0,10875171
TC0400009048.hg.1	TLR2	7097	-0,58898601	6,19436186	-3,06590616	0,00354605	0,10875171
TC0600008129.hg.1	HSP90AB1	3326	0,27017141	10,9824727	3,06584272	0,00354667	0,10875171
TC1700008455.hg.1	YPEL2	388403	-0,71654958	6,62908	-3,06577828	0,00354731	0,10875171
TC0200013541.hg.1	SEMA4C	54910	0,50060172	6,22488081	3,06557551	0,00354932	0,10875171
TC0400012944.hg.1	HSD17B13	345275	-0,3578225	5,02324683	-3,06492891	0,00355574	0,10876792
TC0100014257.hg.1	SSBP3	23648	0,40205037	9,40540642	3,06344216	0,00357053	0,10893844
TC2000009231.hg.1	WFDC8	90199	-0,26360074	4,95558484	-3,0626962	0,00357798	0,10893844
TC1100009097.hg.1	HTR3B	9177	-0,22750184	4,28023461	-3,06259356	0,003579	0,10893844
TC0600010331.hg.1	FAM120B	84498	0,53398851	7,2853427	3,06176103	0,00358733	0,10901233
TC0400006602.hg.1	HGFAC	3083	-0,36934902	5,37731936	-3,06070373	0,00359793	0,10906457

TC1200010336.hg.1	YARS2	51067	0,6548806	7,09004186	3,06041274	0,00360086	0,10906457
TC0X00011174.hg.1	PDZD4	57595	0,65428874	7,51313455	3,059134	0,00361373	0,10911135
TC0200007144.hg.1	LCLAT1	253558	0,88858752	7,90765736	3,05908603	0,00361421	0,10911135
TC1300007718.hg.1	GPC6	10082	-0,27746793	5,05133276	-3,0577644	0,00362756	0,10933574
TC1000009714.hg.1	PRKCQ	5588	1,22776536	7,10141852	3,0564499	0,00364089	0,10955861
TC0100014910.hg.1	TGFBR3	7049	1,61769273	8,21783286	3,0552686	0,0036529	0,10961598
TC1100009200.hg.1	CD3E	916	1,80503091	8,89893168	3,05458949	0,00365982	0,10961598
TC1100008899.hg.1	CEP126	57562	0,50900865	4,92761572	3,05430012	0,00366278	0,10961598
TC1700007042.hg.1	MPRIP	23164	0,54682111	9,18084101	3,05393321	0,00366652	0,10961598
TC0300011936.hg.1	CD47	961	0,50831859	10,5153925	3,05027809	0,00370406	0,11051879
TC0700010081.hg.1	CARD11	84433	0,57159075	8,80684276	3,04981939	0,0037088	0,11051879
TC1900009594.hg.1	ZNF812P	729648	-0,2514796	5,20046591	-3,04925165	0,00371467	0,11051879
TC0100016786.hg.1	UCHL5	51377	0,73362105	8,10679701	3,04782411	0,00372946	0,11078067
TC0700012836.hg.1	DENND11	57189	0,67768751	8,51751175	3,04724162	0,00373552	0,11078239
TC0300007065.hg.1	DLEC1	9940	0,53242024	5,17656975	3,04465398	0,00376252	0,11140001
TC0400010558.hg.1	ATP8A1	10396	0,77654556	6,01694562	3,04332017	0,00377651	0,11140001
TC1900009224.hg.1	PEAK3	374872	-0,39018148	6,38431847	-3,04321956	0,00377757	0,11140001
TC1900011708.hg.1	PDCD2L	84306	0,68029742	7,31981193	3,04294469	0,00378046	0,11140001
TC1900008287.hg.1	NECTIN2	5819	-0,35062736	5,34565656	-3,04194321	0,00379101	0,11153293
TC0400009518.hg.1	SLC25A4	291	0,53628136	5,57360946	3,04086434	0,0038024	0,11169028
TC1500009514.hg.1	RFX7	64864	0,65498008	7,51160314	3,03948254	0,00381704	0,11194233
TC1000012066.hg.1	NSMCE4A	54780	0,49271207	7,05964185	3,03875703	0,00382475	0,1119906
TC1400010039.hg.1	DDX24	57062	0,30877561	10,4761765	3,03806267	0,00383214	0,11202945
TC2200007792.hg.1	SELENOO	83642	-0,27350974	7,72498356	-3,03687548	0,00384481	0,11210398
TC0200016555.hg.1	TBR1	10716	-0,31669421	5,38789138	-3,03612073	0,00385288	0,11210398
TC0900010445.hg.1	NMRK1	54981	0,94007576	6,82082419	3,03611955	0,00385289	0,11210398
TC0500011435.hg.1	LYSMD3	116068	0,74513897	7,91056734	3,03248318	0,00389201	0,11268289
TC0200008005.hg.1	DYSF	8291	-1,00667002	6,2131886	-3,03223534	0,00389469	0,11268289
TC0300013108.hg.1	TNIK	23043	1,2399635	6,88454125	3,03032836	0,00391537	0,11268289
TC1800009222.hg.1	RNF125	54941	1,20389855	7,71196221	3,02980557	0,00392105	0,11268289
TC1400010257.hg.1	HSP90AA1	3320	0,37828098	10,7861652	3,02975805	0,00392157	0,11268289
TC1400009131.hg.1	MAP4K5	11183	0,54290998	7,25767978	3,02937488	0,00392574	0,11268289
TC0X00008754.hg.1	CSAG3	389903	-0,35833309	6,54700204	-3,02931913	0,00392635	0,11268289
TC0200008063.hg.1	DGUOK	1716	0,71587831	8,99005439	3,02916781	0,003928	0,11268289
TC0200007176.hg.1	SLC30A6	55676	0,5565213	8,38950494	3,02872688	0,00393281	0,11268289
TC0500012162.hg.1	BRD8	10902	0,43306788	7,88715023	3,02863832	0,00393378	0,11268289

TC0200009055.hg.1	MARCO	8685	-0,33173632	6,54533283	-3,02750299	0,00394619	0,11284725
TC1900009747.hg.1	TRIR	79002	0,3199746	9,68285245	3,02672563	0,00395471	0,11284725
TC1300007208.hg.1	RNASEH2B	79621	0,67593135	8,58602091	3,02506694	0,00397295	0,11284725
TC0400012934.hg.1	SDAD1	55153	0,46872267	7,54220789	3,02488542	0,00397495	0,11284725
TC0500012100.hg.1	MACROH2A1	9555	-0,44804394	9,66469098	-3,02401936	0,00398451	0,11284725
TC0900009279.hg.1	TOR4A	54863	-0,36499966	6,88001616	-3,02355746	0,00398961	0,11284725
TC0700011298.hg.1	ZNF680	340252	0,42335618	8,03484846	3,02341427	0,0039912	0,11284725
TC1700010035.hg.1	SLC47A2	146802	-0,25807703	5,17926829	-3,0232528	0,00399299	0,11284725
TC0X00009427.hg.1	BCOR	54880	0,42394249	8,19865799	3,02311735	0,00399449	0,11284725
TC0300011979.hg.1	ZBED2	79413	0,71353953	6,51228169	3,02173652	0,00400981	0,113029
TC0300007164.hg.1	SS18L2	51188	0,43101119	8,76534594	3,02143535	0,00401315	0,113029
TC1100006666.hg.1	MMP26	56547	-0,2970054	4,08475887	-3,0197539	0,0040319	0,11338408
TC1900007848.hg.1	HAMP	57817	-0,32920156	6,97689874	-3,01828646	0,00404832	0,11356691
TC0100017022.hg.1	GOLT1A	127845	-0,2310349	5,04390824	-3,01728133	0,00405961	0,11356691
TC0300011222.hg.1	ACTR8	93973	0,47686518	6,39114303	3,01717725	0,00406078	0,11356691
TC0200012418.hg.1	LRPPRC	10128	0,35609471	9,47703972	3,01690178	0,00406388	0,11356691
TC0800012110.hg.1	LY6H	4062	-0,25144624	6,95947163	-3,01580348	0,00407626	0,11356691
TC0100012664.hg.1	NPHP4	261734	-0,21512218	7,14917399	-3,01537978	0,00408104	0,11356691
TC0900010126.hg.1	FOXD4L6	653404	-0,29196051	7,20845132	-3,01439917	0,00409214	0,11356691
TC1400010617.hg.1	PRKCH	5583	1,33082327	7,79236067	3,01372312	0,0040998	0,11356691
TC1600007887.hg.1	RBL2	5934	0,7547028	7,42651568	3,01288602	0,00410931	0,11356691
TC0800007848.hg.1	TRIM55	84675	-0,25280435	4,66252604	-3,01270614	0,00411136	0,11356691
TC1000008502.hg.1	TBC1D12	23232	-0,35902931	4,52061783	-3,01248266	0,0041139	0,11356691
TC1900007356.hg.1	KCNN1	3780	-0,29333941	6,5662623	-3,0124382	0,00411441	0,11356691
TC0600009002.hg.1	MTRES1	51250	0,69665595	5,87922273	3,01209596	0,00411831	0,11356691
TC1100011602.hg.1	UCP2	7351	0,32555191	10,7350278	3,01131588	0,00412721	0,11364268
TC0100008874.hg.1	PRKACB	5567	0,98186302	8,09509796	3,01073199	0,00413388	0,11365702
TC0X00010434.hg.1	SLC25A53	401612	0,5486876	5,50652266	3,00826731	0,00416215	0,11426442
TC0100013709.hg.1	COL8A2	1296	-0,24590515	5,6154568	-3,00598654	0,00418848	0,11481659
TC0400012763.hg.1	S100P	6286	-0,37264741	7,03029482	-3,00518959	0,00419772	0,11482102
TC0700013355.hg.1	NME8	51314	0,31399663	3,69858039	3,00490055	0,00420107	0,11482102
TC1900008435.hg.1	SELENOW	6415	0,50510051	9,58545731	3,00291312	0,00422421	0,11528274
TC1500010003.hg.1	LOC283731	283731	-0,26775799	5,14733115	-3,00137715	0,00424217	0,11560213
TC1400010778.hg.1	TRIP11	9321	0,6703521	5,99614634	2,99966038	0,00426232	0,1159804
TC1400008715.hg.1	EFS	10278	-0,39555874	6,5370327	-2,99708507	0,00429273	0,11633751
TC0200016442.hg.1	MSH2	4436	0,68417543	6,32723751	2,99698204	0,00429395	0,11633751

TC1200012752.hg.1	KLRC3	3823	1,46531543	6,48713299	2,99679663	0,00429615	0,11633751
TC0400007225.hg.1	DTHD1	401124	0,71883251	5,68729555	2,99630721	0,00430196	0,11633751
TC0200009370.hg.1	ZNF285CP	646915	-0,2103754	4,15719583	-2,99588827	0,00430693	0,11633751
TC0700013509.hg.1	DAGLB	221955	-0,36542003	8,06135586	-2,99500804	0,0043174	0,11642081
TC0100011487.hg.1	TRAF5	7188	0,72466676	6,7599383	2,99457047	0,00432262	0,11642081
TC0X00009199.hg.1	ADGRG2	10149	-0,21973318	4,01091303	-2,9933809	0,00433682	0,11646583
TC1200008223.hg.1	GLIPR1L2	144321	-0,27277205	4,59035531	-2,99302083	0,00434113	0,11646583
TC1900011139.hg.1	SLC17A7	57030	-0,26295916	4,65386474	-2,99284813	0,0043432	0,11646583
TC0900009785.hg.1	LINGO2	158038	0,56174541	4,62044498	2,99152819	0,00435904	0,11672106
TC0100018499.hg.1	MUC1	4582	-0,37917391	6,80572538	-2,99042815	0,00437227	0,11690612
TC1700011366.hg.1	INTS2	57508	0,70147917	7,44280551	2,98845278	0,00439614	0,11737442
TC1700010451.hg.1	RDM1	201299	0,37096339	4,56781756	2,98730141	0,00441011	0,11757743
TC1900007622.hg.1	ZNF254	9534	0,3703791	8,6264486	2,98650747	0,00441976	0,11766506
TC0100013021.hg.1	HSPB7	27129	-0,38209775	5,94204493	-2,9858682	0,00442755	0,11770282
TC0X00006489.hg.1	ARSF	416	-0,28566729	4,90602724	-2,98247223	0,00446914	0,11841013
TC1000007024.hg.1	KIAA1217	56243	-0,23318662	5,64217146	-2,98204031	0,00447446	0,11841013
TC1900009608.hg.1	EIF3G	8666	0,36650898	8,93970135	2,9818617	0,00447666	0,11841013
TC0500009987.hg.1	MED10	84246	0,4527343	9,5278903	2,9809396	0,00448803	0,11841013
TC1200008474.hg.1	SOCS2	8835	0,69829194	6,74667783	2,98021186	0,00449703	0,11841013
TC1900007142.hg.1	ZSWIM4	65249	-0,30490563	6,54495267	-2,9799587	0,00450016	0,11841013
TC1400009388.hg.1	PPP2R5E	5529	0,48412184	7,60010367	2,97959747	0,00450464	0,11841013
TC2100008081.hg.1	CLDN14	23562	-0,27437028	5,25530261	-2,97953356	0,00450543	0,11841013
TC0400011048.hg.1	PPEF2	5470	-0,1760535	3,81346501	-2,97898147	0,00451228	0,11842164
TC0100017365.hg.1	EPRS1	2058	0,39049192	8,59382397	2,97772011	0,00452796	0,11843389
TC1700009032.hg.1	TNRC6C	57690	0,35877847	7,36364158	2,97757074	0,00452982	0,11843389
TC0600007530.hg.1	HLA-E	3133	0,3486651	10,6076411	2,97739774	0,00453197	0,11843389
TC1000007462.hg.1	ZNF22	7570	0,38063062	7,51005194	2,9764102	0,0045443	0,11858818
TC1700007326.hg.1	TBC1D3P5	440419	-0,31176133	5,63178386	-2,97520768	0,00455935	0,11860157
TC0200010264.hg.1	MFSD6	54842	0,51048582	9,04605452	2,97513694	0,00456023	0,11860157
TC2200009142.hg.1	MLC1	23209	1,35858009	6,4663453	2,97483101	0,00456407	0,11860157
TC1700011060.hg.1	FAM117A	81558	0,53212107	7,58142295	2,97187997	0,00460123	0,11939929
TC2200006688.hg.1	LRRC74B	400891	-0,36997671	5,11784914	-2,97102822	0,00461201	0,11951112
TC1000008568.hg.1	FRAT1	10023	-0,41423921	7,51465724	-2,97009319	0,00462387	0,11965061
TC0100016366.hg.1	KIFAP3	22920	0,78772725	6,16828877	2,96908512	0,00463668	0,11971143
TC1200012799.hg.1	SARNP	84324	0,5270048	6,81817121	2,96851125	0,00464399	0,11971143
TC0200009871.hg.1	CSRNP3	80034	-0,32584077	4,88393144	-2,9683811	0,00464565	0,11971143

TC1600008190.hg.1	SLC7A6	9057	0,47965578	9,69269001	2,96516396	0,00468686	0,1202426
TC0300010358.hg.1	XPC	7508	0,7350769	9,43324969	2,96501588	0,00468876	0,1202426
TC1800007506.hg.1	TNFRSF11A	8792	0,37596349	5,39762297	2,9646837	0,00469303	0,1202426
TC0600011875.hg.1	LRRC73	221424	-0,31355011	6,43094139	-2,96452809	0,00469504	0,1202426
TC0500010599.hg.1	OXCT1	5019	0,645387	6,40769057	2,96423564	0,00469881	0,1202426
TC1900009045.hg.1	RPS5	6193	0,26252805	11,4995791	2,96344197	0,00470905	0,12027747
TC1200010900.hg.1	SMARCC2	6601	0,25868536	10,8551354	2,96312145	0,00471319	0,12027747
TC1000009404.hg.1	PWWP2B	170394	-0,27678752	7,89773181	-2,9618409	0,00472977	0,12037285
TC1700009249.hg.1	UTS2R	2837	-0,38398424	6,75507016	-2,96182649	0,00472996	0,12037285
TC0500009417.hg.1	TLX3	30012	-0,29921769	6,0670477	-2,96044449	0,00474791	0,12041319
TC0500009093.hg.1	NDST1	3340	-0,34678794	7,20459456	-2,96037373	0,00474884	0,12041319
TC0200012925.hg.1	C1D	10438	0,52650629	6,3017265	2,9598872	0,00475517	0,12041319
TC1700008862.hg.1	RAB37	326624	0,74065188	6,15756831	2,9592548	0,00476342	0,12041319
TC1900008164.hg.1	RPS19	6223	0,29174351	10,7878969	2,95894994	0,0047674	0,12041319
TC0600010017.hg.1	TMEM181	57583	0,77155271	7,08210731	2,95845874	0,00477383	0,12041319
TC0600012628.hg.1	NDUFAF4	29078	0,52707944	6,41596359	2,9582037	0,00477716	0,12041319
TC1700012426.hg.1	WNT3	7473	-0,24474683	6,61941752	-2,95703459	0,00479249	0,12063495
TC1100012495.hg.1	DDX6	1656	0,32973039	10,3569533	2,95624149	0,00480291	0,12073285
TC0100006604.hg.1	PRDM16	63976	-0,30597077	5,61727428	-2,9536013	0,00483777	0,12136408
TC0900011829.hg.1	RALGDS	5900	0,49477514	7,12992047	2,95334505	0,00484116	0,12136408
TC0700011973.hg.1	TAF6	6878	0,3789443	7,81314881	2,95230232	0,004855	0,12154609
TC0200007237.hg.1	CRIM1	51232	0,47849787	7,21480264	2,95153889	0,00486516	0,12163552
TC0900008202.hg.1	TGFBR1	7046	1,09998393	6,9792032	2,95012367	0,00488404	0,12194249
TC0100011573.hg.1	SMYD2	56950	0,47846541	7,15121326	2,94956198	0,00489155	0,12196522
TC1200009876.hg.1	CD69	969	1,19911023	7,56976897	2,94895299	0,0048997	0,12200395
TC0900012278.hg.1	ST6GALNAC6	30815	0,51213864	8,79595061	2,94774013	0,00491599	0,1221493
TC1600011524.hg.1	ZNF689	115509	-0,29875209	6,10409518	-2,94753357	0,00491876	0,1221493
TC1200012700.hg.1	C12orf75	387882	1,05193028	6,99895257	2,94364803	0,00497129	0,12328806
TC1700011319.hg.1	APPBP2	10513	0,47047315	5,79266498	2,94212593	0,00499201	0,12363596
TC0500008568.hg.1	SLC12A2	6558	0,78877484	6,1987461	2,94079818	0,00501015	0,12380609
TC0500010523.hg.1	GDNF	2668	-0,26170374	6,85189756	-2,93955064	0,00502725	0,12380609
TC0400007609.hg.1	REST	5978	0,6791015	8,02795827	2,93946224	0,00502847	0,12380609
TC1300006683.hg.1	GTF3A	2971	0,31706119	10,0142413	2,93900107	0,0050348	0,12380609
TC0300007517.hg.1	CACNA1D	776	-0,30890431	5,50216724	-2,93883827	0,00503704	0,12380609
TC0800008715.hg.1	ZHX2	22882	0,65352213	7,8996097	2,93868951	0,00503909	0,12380609
TC1600008228.hg.1	CYB5B	80777	0,60117142	7,39093009	2,93798801	0,00504875	0,12387871

TC0100009568.hg.1	MAN1A2	10905	0,60115999	9,80661893	2,93641847	0,00507043	0,12424558
TC1800008281.hg.1	TMEM241	85019	0,60291078	6,34615576	2,93450335	0,00509699	0,12473116
TC1900006678.hg.1	CREB3L3	84699	-0,24587728	4,42958609	-2,9330295	0,00511753	0,12495849
TC2000009782.hg.1	DIDO1	11083	0,5302304	7,7547072	2,93286604	0,00511981	0,12495849
TC1200012258.hg.1	PITPNM2	57605	0,80678176	5,36368967	2,93236883	0,00512676	0,12496298
TC1100008069.hg.1	CNIH2	254263	-0,38323358	5,19127671	-2,93167229	0,0051365	0,12503562
TC0400012173.hg.1	SFRP2	6423	-0,22117461	4,05855986	-2,93040619	0,00515427	0,12530291
TC0200011020.hg.1	SP140	11262	0,83823113	9,59904624	2,92932062	0,00516954	0,12550911
TC1300008083.hg.1	ATP11AUN	400165	-0,25695784	4,36843593	-2,92821164	0,00518519	0,12572381
TC1800007895.hg.1	METTL4	64863	0,49480433	7,71982993	2,92632007	0,00521198	0,12618072
TC0900012179.hg.1	SPTAN1	6709	0,5906641	7,78636845	2,9252332	0,00522743	0,12618072
TC0600012428.hg.1	UBE3D	90025	0,60601633	5,78377021	2,92496246	0,00523129	0,12618072
TC0100018513.hg.1	SNHG28	284677	-0,27144422	6,09553987	-2,92473844	0,00523448	0,12618072
TC0900009579.hg.1	ZDHHC21	340481	0,64348778	7,17775054	2,92427864	0,00524104	0,12618072
TC2000008072.hg.1	PPDPF	79144	-0,31917177	6,56255475	-2,92400068	0,00524501	0,12618072
TC1500007091.hg.1	CTDSPL2	51496	0,43917048	8,37259274	2,9230318	0,00525886	0,12634951
TC0100012055.hg.1	GGPS1	9453	0,89256525	7,37730612	2,91933533	0,00531204	0,12746126
TC0100014247.hg.1	IFT25	51668	0,46287178	7,17599163	2,91880431	0,00531972	0,12747997
TC0700008870.hg.1	CAV2	858	-0,23855596	4,40293342	-2,91761261	0,00533699	0,12767947
TC0400006616.hg.1	ADRA2C	152	-0,35493234	6,86844267	-2,91727692	0,00534186	0,12767947
TC1600011346.hg.1	SEC14L5	9717	-0,23833815	4,54822919	-2,91672711	0,00534985	0,1277053
TC0700013371.hg.1	ZNF138	7697	0,62191818	8,1739102	2,91483158	0,0053775	0,1281995
TC1500007838.hg.1	ISLR2	57611	-0,22344531	4,36774307	-2,91341559	0,00539823	0,12832836
TC0100015945.hg.1	THBS3	7059	-0,32866648	7,28810605	-2,91336897	0,00539892	0,12832836
TC0100016302.hg.1	CD247	919	0,87430539	7,36263998	2,9130404	0,00540374	0,12832836
TC0500010580.hg.1	PRKAA1	5562	0,46194606	8,59772211	2,91101491	0,00543356	0,12887085
TC1200011054.hg.1	PPM1H	57460	-0,26696832	4,15518672	-2,91005039	0,00544781	0,12890449
TC2200006457.hg.1	POTEH	23784	-0,25036975	4,24546371	-2,90969182	0,00545312	0,12890449
TC0400011108.hg.1	CNOT6L	246175	0,74262897	8,76714782	2,9091303	0,00546144	0,12890449
TC0100010335.hg.1	USP21	27005	0,60017496	7,17250499	2,90903296	0,00546288	0,12890449
TC1600008199.hg.1	ZFP90	146198	0,45298896	7,46083288	2,90659602	0,00549914	0,12959459
TC0200013595.hg.1	MGAT4A	11320	1,16272544	7,7059616	2,90517034	0,00552046	0,12993122
TC0900009922.hg.1	SPATA31F3	100129969	-0,27567135	4,33741025	-2,90460959	0,00552887	0,12994711
TC0X00009007.hg.1	PUDP	8226	-0,32894647	4,50802484	-2,90364229	0,00554339	0,12994711
TC0500011316.hg.1	RPS23	6228	0,13861701	12,6917279	2,90339525	0,00554711	0,12994711
TC0700013420.hg.1	ARPC1B	10095	-0,51188183	8,19528665	-2,902761	0,00555666	0,12994711

TC1900006504.hg.1	GRIN3B	116444	-0,35460155	5,62321132	-2,90267553	0,00555795	0,12994711
TC0600010799.hg.1	GCM2	9247	-0,26925101	5,39915069	-2,90231828	0,00556333	0,12994711
TC0200016584.hg.1	HSPE1	3336	0,52170213	10,2511581	2,90127182	0,00557914	0,12999209
TC2100008506.hg.1	MORC3	23515	0,61845084	7,31241375	2,90125939	0,00557933	0,12999209
TC1400008423.hg.1	TDRD9	122402	-0,28281528	4,86076214	-2,90071663	0,00558755	0,13001956
TC1100012052.hg.1	CCDC82	79780	0,63337566	7,22796342	2,89963691	0,00560393	0,13023665
TC0100017748.hg.1	SIPA1L2	57568	-0,37370352	6,68765562	-2,89669416	0,00564879	0,13096013
TC1700012209.hg.1	SLC47A1	55244	-0,3735091	5,94415281	-2,8966653	0,00564923	0,13096013
TC1500010429.hg.1	CIB1	10519	0,48713536	8,76336406	2,89545987	0,00566771	0,13119063
TC0700009688.hg.1	NOS3	4846	-0,23154975	5,08327748	-2,89509078	0,00567338	0,13119063
TC1500009348.hg.1	FBN1	2200	-0,26972106	5,04019728	-2,89369046	0,00569493	0,13122422
TC0500008698.hg.1	JADE2	23338	0,4518977	6,75389525	2,89356566	0,00569686	0,13122422
TC0800006880.hg.1	PCM1	5108	0,30781538	8,77506622	2,89323959	0,00570189	0,13122422
TC0Y00006487.hg.1	ZFY	7544	0,25564383	6,22383484	2,89315193	0,00570324	0,13122422
TC0400012938.hg.1	RASGEF1B	153020	-1,09507264	6,57928579	-2,89169036	0,00572585	0,13158054
TC0600012038.hg.1	MCM3	4172	0,40879522	6,42433558	2,88897746	0,00576803	0,13200207
TC0100010553.hg.1	FIRRM	55732	0,44891425	4,9810415	2,88895311	0,00576841	0,13200207
TC1400007094.hg.1	KLHDC2	23588	0,71856699	6,84374372	2,88850087	0,00577547	0,13200207
TC1500009446.hg.1	LEO1	123169	0,48192854	6,87332423	2,88705985	0,00579802	0,13200207
TC0400012969.hg.1	SETD7	80854	-0,26364172	6,72009667	-2,88681132	0,00580192	0,13200207
TC1400007491.hg.1	PALS1	64398	-0,31453783	5,55732425	-2,88669433	0,00580376	0,13200207
TC0400012386.hg.1	HPF1	54969	0,51443873	7,02592729	2,88662566	0,00580484	0,13200207
TC1400007310.hg.1	KIAA0586	9786	0,72769653	8,10175697	2,88649605	0,00580687	0,13200207
TC0600014357.hg.1	LRP11	84918	-0,2730439	5,15623461	-2,88608595	0,00581331	0,13200207
TC1300009026.hg.1	PCDH8	5100	-0,24885238	4,52560626	-2,88593812	0,00581564	0,13200207
TC1000010022.hg.1	PIP4K2A	5305	0,79726893	10,7596445	2,88486987	0,00583245	0,13222137
TC1700010195.hg.1	SLC46A1	113235	-0,28625349	6,99927344	-2,88403851	0,00584557	0,13224574
TC1900011932.hg.1	ALKBH6	84964	0,45791197	7,13512431	2,8838948	0,00584784	0,13224574
TC2200009180.hg.1	RABL2B	11158	0,42048128	7,7746384	2,88294731	0,00586284	0,13242268
TC0600011127.hg.1	H1-2	3006	0,43814376	8,99745066	2,88159126	0,00588435	0,13270201
TC0100006903.hg.1	AADACL3	126767	-0,29966694	5,09735294	-2,88126339	0,00588957	0,13270201
TC2000006799.hg.1	DSTN	11034	1,0967469	7,47953204	2,87998389	0,00590995	0,1329911
TC1200010569.hg.1	ASB8	140461	0,4174691	9,06138749	2,87955557	0,00591679	0,1329911
TC0700007178.hg.1	SEPTIN7	989	0,43508361	8,4630276	2,87887548	0,00592767	0,13301088
TC0300009706.hg.1	POLR2H	5437	0,45753587	8,98903146	2,87860042	0,00593207	0,13301088
TC0600014101.hg.1	MICA	100507436	0,37543967	6,78637317	2,87704573	0,00595702	0,1331229

TC1500010261.hg.1	BNC1	646	-0,2212409	4,55816675	-2,87655197	0,00596496	0,1331229
TC0300010082.hg.1	FAM157A	728262	-0,66498499	7,74543112	-2,87650676	0,00596569	0,1331229
TC1100009231.hg.1	FOXR1	283150	-0,21536508	4,15249512	-2,87621827	0,00597034	0,1331229
TC1100007787.hg.1	CD6	923	0,44491326	6,99146157	2,87604711	0,00597309	0,1331229
TC1900007100.hg.1	MAST1	22983	-0,23105692	5,00366517	-2,87529155	0,00598528	0,13323385
TC1000009287.hg.1	FOXI2	399823	-0,25896865	5,9472378	-2,87367538	0,00601144	0,13365495
TC2200008898.hg.1	TTLL1	25809	0,44353198	5,91544427	2,872348	0,00603299	0,13397303
TC1500010897.hg.1	CHRNB4	1143	-0,35241787	5,67348565	-2,86942347	0,00608074	0,1348713
TC0500009321.hg.1	MAT2B	27430	0,55565186	8,70987993	2,86880025	0,00609096	0,13493601
TC1900006655.hg.1	MRPL54	116541	-0,34023316	7,35753592	-2,86762862	0,00611022	0,13509651
TC0300014095.hg.1	TM4SF19	116211	0,35670315	5,17404362	2,86685367	0,00612299	0,13509651
TC0100006818.hg.1	PGD	5226	-0,4094134	5,98085203	-2,86659659	0,00612723	0,13509651
TC0200011509.hg.1	MYT1L	23040	-0,1873852	5,36196288	-2,86658297	0,00612746	0,13509651
TC1700009670.hg.1	ALOXE3	59344	-0,26800055	5,07428271	-2,86603453	0,00613651	0,13513496
TC0100010674.hg.1	GPR52	9293	0,60802324	8,18614216	2,86400731	0,0061701	0,13546026
TC0200012959.hg.1	NFU1	27247	0,51481774	7,07086346	2,86381803	0,00617325	0,13546026
TC0200016553.hg.1	DAPL1	92196	-0,28948116	4,84854157	-2,863816	0,00617328	0,13546026
TC0200016666.hg.1	LHCGR	3973	-0,2119613	4,68807297	-2,8597709	0,00624085	0,13659492
TC1600008155.hg.1	PLEKHG4	25894	-0,33982385	5,53337512	-2,85954194	0,00624469	0,13659492
TC0900008653.hg.1	MORN5	254956	-0,2807931	4,99700145	-2,85939441	0,00624717	0,13659492
TC1700006667.hg.1	USP6	9098	-0,22677018	6,70967587	-2,85818537	0,00626752	0,13687781
TC1500008232.hg.1	ISG20	3669	0,62171252	10,2678905	2,85502528	0,00632099	0,13788259
TC0400008165.hg.1	ATOH1	474	-0,34010103	5,42670896	-2,85454959	0,00632907	0,13789616
TC0300009892.hg.1	ATP13A5-AS1	100874218	-0,25308378	4,62951688	-2,85410727	0,0063366	0,13789754
TC0X00007557.hg.1	MED12	9968	0,53591276	8,69264797	2,85364513	0,00634447	0,13790644
TC0500008229.hg.1	PAM	5066	0,6625094	5,800371	2,84960293	0,00641372	0,13924791
TC1100012960.hg.1	DNHD1	144132	-0,48738955	7,36203636	-2,84766859	0,00644711	0,13980849
TC0200011188.hg.1	AGAP1	116987	0,60856692	5,45218736	2,84677549	0,00646258	0,13997965
TC0100018142.hg.1	SH3BP5L	80851	-0,25920768	6,83641244	-2,84579444	0,00647961	0,14011022
TC0900011582.hg.1	NIBAN2	64855	-0,58235394	6,17096011	-2,84555506	0,00648378	0,14011022
TC0800010258.hg.1	KAT6A	7994	0,54597191	8,16328925	2,84413664	0,00650849	0,14047997
TC0100013650.hg.1	A3GALT2	127550	-0,3990622	4,95586145	-2,84272698	0,00653314	0,14080142
TC0200008556.hg.1	ZAP70	7535	1,29942794	8,22097008	2,84198639	0,00654612	0,14080142
TC0100006602.hg.1	ACTRT2	140625	-0,30677011	4,81057737	-2,84109741	0,00656174	0,14080142
TC1500010712.hg.1	GOLGA8N	643699	0,8463768	9,46923265	2,84094043	0,0065645	0,14080142
TC1900007508.hg.1	ZNF430	80264	0,37669393	7,79221809	2,84076109	0,00656766	0,14080142

TC0800006496.hg.1	MYOM2	9172	1,02795733	5,63873713	2,84063487	0,00656988	0,14080142
TC1700008734.hg.1	AMZ2	51321	0,54313637	8,45667734	2,84001825	0,00658074	0,14080142
TC0300008839.hg.1	ACP3	55	-0,85620575	4,9975873	-2,8390384	0,00659804	0,14080142
TC0100015786.hg.1	POGZ	23126	0,48020826	8,85074719	2,83865021	0,00660491	0,14080142
TC1800006448.hg.1	TYMS	7298	0,62424935	5,52440039	2,83851448	0,00660731	0,14080142
TC2200007271.hg.1	NCF4	4689	-0,66644617	8,30614077	-2,83830607	0,006611	0,14080142
TC0600013528.hg.1	LATS1	9113	0,74924681	9,41525178	2,83741962	0,00662672	0,14080142
TC0200010795.hg.1	SLC11A1	6556	-1,18220519	8,50839978	-2,83579727	0,00665557	0,14080142
TC0200008632.hg.1	RPL31	6160	0,19400051	11,8209217	2,83550214	0,00666083	0,14080142
TC0100010060.hg.1	RPS27	6232	0,19702032	11,5592389	2,83541331	0,00666241	0,14080142
TC0900009139.hg.1	FCN2	2220	-0,26846767	6,66359746	-2,83514443	0,00666721	0,14080142
TC1600011237.hg.1	PABPN1L	390748	-0,2794789	6,5756223	-2,83407322	0,00668636	0,14080142
TC1100007017.hg.1	TMEM86A	144110	-0,34274031	6,89014092	-2,83393661	0,0066888	0,14080142
TC0200012608.hg.1	PSME4	23198	0,60854774	8,04235153	2,83387012	0,00668999	0,14080142
TC0100018261.hg.1	GSTM4	2948	0,55894707	6,06057896	2,83374242	0,00669228	0,14080142
TC0300008482.hg.1	ADPRH	141	-0,437549	6,16161462	-2,83318311	0,0067023	0,14080142
TC0300006669.hg.1	LSM3	27258	0,72795413	6,82782933	2,83273522	0,00671034	0,14080142
TC1400009980.hg.1	CCDC88C	440193	0,67728747	7,56688392	2,83272755	0,00671048	0,14080142
TC0100018496.hg.1	PYGO2	90780	0,46980797	7,44449628	2,83263218	0,00671219	0,14080142
TC0200014404.hg.1	MCM6	4175	0,68591919	5,5352749	2,83193001	0,00672481	0,14080142
TC2200008914.hg.1	EFCAB6	64800	-0,22005189	4,63793826	-2,83109452	0,00673986	0,14080142
TC1400010270.hg.1	ANKRD9	122416	-0,29591595	5,66551304	-2,83105259	0,00674062	0,14080142
TC1900011926.hg.1	ZNF91	7644	0,37546371	7,81267927	2,83080782	0,00674503	0,14080142
TC0300011849.hg.1	ABI3BP	25890	-0,30980664	4,74546198	-2,83060853	0,00674863	0,14080142
TC0500013373.hg.1	SRA1	10011	-0,38081286	8,48977793	-2,82988275	0,00676174	0,14080142
TC1100007818.hg.1	DAGLA	747	-0,32961411	6,87574014	-2,82966036	0,00676576	0,14080142
TC0100007747.hg.1	GJB3	2707	-0,18517423	4,59286228	-2,82918172	0,00677443	0,14080142
TC0100018489.hg.1	VPS72	6944	0,58088859	7,70101655	2,82824884	0,00679135	0,14080142
TC0600007557.hg.1	MUC22	100507679	-0,29315739	5,53909398	-2,82814059	0,00679331	0,14080142
TC1100007293.hg.1	LDLRAD3	143458	-0,40705071	6,65113107	-2,82659034	0,00682152	0,14080142
TC0500007395.hg.1	HSPB3	8988	-0,26965377	5,32204659	-2,82651521	0,00682289	0,14080142
TC0200010582.hg.1	FASTKD2	22868	0,48446821	6,13445509	2,82640489	0,0068249	0,14080142
TC1100007414.hg.1	LINC02687	399886	-0,30237073	5,07083613	-2,82627208	0,00682733	0,14080142
TC1800007960.hg.1	ZBTB14	7541	0,73852578	6,19689849	2,82553763	0,00684074	0,14080142
TC1500008554.hg.1	LRRK1	79705	-0,37951115	8,16952535	-2,82549299	0,00684155	0,14080142
TC0100016252.hg.1	LMX1A	4009	-0,29738933	6,97886175	-2,82521593	0,00684662	0,14080142

TC1100008049.hg.1	BANF1	8815	0,38498547	9,30714194	2,8246555	0,00685688	0,14080142
TC2000008975.hg.1	C20orf173	140873	-0,30321249	5,48035019	-2,82457833	0,00685829	0,14080142
TC0100011215.hg.1	LGR6	59352	0,74553073	5,32874064	2,82397967	0,00686927	0,14080142
TC0500009250.hg.1	UBLCP1	134510	0,63633366	8,17843046	2,82373659	0,00687373	0,14080142
TC1400010772.hg.1	DIO2	1734	-0,26531257	5,49640715	-2,82339687	0,00687997	0,14080142
TC0300014041.hg.1	RUVBL1	8607	0,61729362	7,26118641	2,82331052	0,00688156	0,14080142
TC2100007474.hg.1	PRMT2	3275	0,57828954	8,70571579	2,8225113	0,00689626	0,14094619
TC1900009352.hg.1	SEMA6B	10501	-0,2736716	6,04825823	-2,82191987	0,00690716	0,14101297
TC0100015950.hg.1	ENTREP3	10712	-0,28209348	6,5602986	-2,82107258	0,00692281	0,14111331
TC2200009257.hg.1	TCN2	6948	-0,52733178	6,03872121	-2,82082662	0,00692735	0,14111331
TC0300009497.hg.1	TMEM212	389177	-0,27885779	4,94169423	-2,81973207	0,00694762	0,14129296
TC1900009405.hg.1	FUT3	2525	-0,31831433	6,08194272	-2,81915803	0,00695827	0,14129296
TC1400008945.hg.1	RALGAPA1	253959	0,49529723	8,20744141	2,8184239	0,00697192	0,14129296
TC1500008703.hg.1	CYFIP1	23191	-0,38701924	7,02173782	-2,81792471	0,00698121	0,14129296
TC2100007949.hg.1	MIS18A	54069	0,57822204	7,10261167	2,81654602	0,00700694	0,14129296
TC1400009568.hg.1	SLC8A3	6547	-0,36007733	5,52759056	-2,81608158	0,00701562	0,14129296
TC0900010311.hg.1	FAM27E3	100131997	0,417352	6,48001935	2,81604671	0,00701627	0,14129296
TC0200008675.hg.1	IL18RAP	8807	1,63208558	7,47123124	2,81589108	0,00701919	0,14129296
TC1600011550.hg.1	BEAN1-AS1	101927726	-0,24599507	4,85021992	-2,81563931	0,0070239	0,14129296
TC0900011603.hg.1	ENG	2022	-0,39958008	7,57560158	-2,81517097	0,00703268	0,14129296
TC0600010954.hg.1	KIF13A	63971	-0,36236474	6,64277639	-2,81505048	0,00703494	0,14129296
TC0700012506.hg.1	PAX4	5078	-0,25236487	6,51399031	-2,81369208	0,00706046	0,14129296
TC0500009649.hg.1	RMND5B	64777	-0,3958292	7,40267661	-2,81323092	0,00706915	0,14129296
TC0900012029.hg.1	ENTPD2	954	-0,28487627	5,89232841	-2,81305964	0,00707238	0,14129296
TC0600011491.hg.1	HLA-DRB5	3127	0,94257585	7,35487747	2,81299842	0,00707353	0,14129296
TC1100013012.hg.1	STX3	6809	-0,67280533	5,85840572	-2,81288077	0,00707575	0,14129296
TC0300011055.hg.1	C3orf84	646498	-0,24372478	4,83341949	-2,81265501	0,00708001	0,14129296
TC0100006624.hg.1	SMIM1	388588	-0,24768818	7,31382004	-2,81234662	0,00708583	0,14129296
TC0600014374.hg.1	PHF10	55274	0,49935735	8,9985712	2,81230762	0,00708657	0,14129296
TC1800009286.hg.1	RPL17-C18orf32	100526842	0,18748312	11,5252438	2,81217237	0,00708912	0,14129296
TC1300007032.hg.1	SERP2	387923	-0,24375034	4,72318334	-2,81133662	0,00710493	0,14129468
TC1600006453.hg.1	PDIA2	64714	-0,36895825	6,80397633	-2,81126821	0,00710622	0,14129468
TC1700010927.hg.1	CDC27	996	0,35257363	9,0790621	2,81069092	0,00711716	0,14129468
TC1000009452.hg.1	VENTX	27287	-0,33626032	6,84605498	-2,8096351	0,00713721	0,14129468
TC1900011799.hg.1	LILRB1	10859	-0,61159609	8,04799101	-2,80950093	0,00713976	0,14129468
TC1200010842.hg.1	PPP1R1A	5502	-0,26741658	6,71806331	-2,80949937	0,00713979	0,14129468

TC0200012252.hg.1	STRN	6801	0,35343075	8,77843871	2,80934418	0,00714274	0,14129468
TC1500010404.hg.1	MESP1	55897	-0,30362428	7,66614855	-2,80862298	0,00715647	0,14131398
TC1700006735.hg.1	SLC2A4	6517	-0,21541449	5,78579455	-2,80832776	0,0071621	0,14131398
TC1600008868.hg.1	CDH15	1013	-0,26801308	6,4130401	-2,80808537	0,00716672	0,14131398
TC0900010854.hg.1	NUTM2F	54754	-0,30891344	6,24098132	-2,80768818	0,00717431	0,14131398
TC0700013013.hg.1	ZNF467	168544	-0,25457881	6,97235392	-2,80631238	0,00720063	0,14154532
TC1200007251.hg.1	RESF1	55196	0,43429394	8,68473202	2,80489847	0,00722778	0,14154532
TC0X00009580.hg.1	CFP	5199	-0,45354805	7,53523386	-2,80473325	0,00723096	0,14154532
TC1700012317.hg.1	NPB	256933	-0,23690642	7,70605377	-2,80411154	0,00724293	0,14154532
TC1200010109.hg.1	LDHB	3945	0,63815855	8,30879817	2,80393584	0,00724632	0,14154532
TC0100017663.hg.1	ACTA1	58	-0,2624201	7,70183683	-2,80378607	0,00724921	0,14154532
TC0600011927.hg.1	SUPT3H	8464	0,71197597	7,27796204	2,80360626	0,00725268	0,14154532
TC0100017878.hg.1	ZP4	57829	-0,20372587	4,17026892	-2,80338504	0,00725695	0,14154532
TC0600012647.hg.1	FBXL4	26235	0,46806464	7,0035574	2,80323147	0,00725991	0,14154532
TC1500007645.hg.1	MAP2K5	5607	0,69923914	8,23665135	2,80278144	0,00726861	0,14154532
TC0300008361.hg.1	SIDT1	54847	0,86099321	7,63468913	2,80263759	0,00727139	0,14154532
TC0600007636.hg.1	RNF5	6048	0,2916251	7,80578877	2,80214796	0,00728087	0,14154532
TC0100016206.hg.1	SH2D1B	117157	1,73817661	6,59648845	2,80190118	0,00728565	0,14154532
TC1000006782.hg.1	ECHDC3	79746	-0,40599644	5,18698685	-2,80100187	0,00730309	0,1416105
TC0600011661.hg.1	STK38	11329	0,42285723	10,0168254	2,80093806	0,00730433	0,1416105
TC1000012485.hg.1	CC2D2B	387707	-0,24208527	3,89500493	-2,8005216	0,00731242	0,1416188
TC1700009750.hg.1	TMEM220	388335	0,53929162	5,28919625	2,79964726	0,00732944	0,14176844
TC1200007835.hg.1	COQ10A	93058	0,51051143	7,70013	2,79933662	0,0073355	0,14176844
TC2200008800.hg.1	DNAJB7	150353	-0,18332117	4,03898239	-2,797853	0,00736448	0,14188545
TC0300013447.hg.1	RFC4	5984	0,50812846	7,70807769	2,79785254	0,00736449	0,14188545
TC0100010144.hg.1	RXFP4	339403	-0,22589051	5,39055445	-2,7974953	0,00737148	0,14188545
TC1600011374.hg.1	SPN	6693	0,53560538	9,01433456	2,79707596	0,0073797	0,14188545
TC0X00009980.hg.1	CXorf65	158830	0,63678293	8,06251549	2,79704171	0,00738037	0,14188545
TC0500011759.hg.1	ATG12	9140	0,45021596	8,19480183	2,79537124	0,0074132	0,14188545
TC1600007440.hg.1	ALDOA	226	-0,35704214	9,10894331	-2,7949211	0,00742207	0,14188545
TC0300006894.hg.1	CMC1	152100	1,22868063	7,89873455	2,79481209	0,00742421	0,14188545
TC0300011051.hg.1	USP19	10869	-0,41792772	7,00943955	-2,79465273	0,00742736	0,14188545
TC0100018556.hg.1	BLACAT1	101669762	-0,32590525	4,96232519	-2,79456992	0,00742899	0,14188545
TC2200009353.hg.1	SUN2	25777	0,38481515	8,68706831	2,79454179	0,00742955	0,14188545
TC0800011479.hg.1	SYBU	55638	-0,28362026	4,57517898	-2,79433106	0,0074337	0,14188545
TC0400011548.hg.1	LEF1	51176	0,91275864	9,08051677	2,79321575	0,00745575	0,14210207

TC0500007122.hg.1	BRIX1	55299	0,50932238	6,86235309	2,79191036	0,00748163	0,14210207
TC0900012202.hg.1	KIAA2026	158358	0,44564921	9,10080247	2,79123533	0,00749505	0,14210207
TC1700008151.hg.1	NFE2L1	4779	0,40000146	9,32827057	2,79122064	0,00749534	0,14210207
TC2000008722.hg.1	ACSS1	84532	0,32441111	8,26552587	2,79111059	0,00749753	0,14210207
TC0100007191.hg.1	PLA2G2F	64600	-0,29642209	5,55513435	-2,79081022	0,00750351	0,14210207
TC0400010580.hg.1	GNPDA2	132789	0,57750926	5,95675914	2,79065832	0,00750654	0,14210207
TC0500012459.hg.1	PDGFRB	5159	0,69883504	5,63029776	2,79065592	0,00750658	0,14210207
TC1400007555.hg.1	PLEKHD1	400224	-0,33222695	4,97372889	-2,78851785	0,00754928	0,14265361
TC0500009673.hg.1	ZNF454	285676	-0,22407172	4,54547371	-2,78818366	0,00755598	0,14265361
TC2000009053.hg.1	TTI1	9675	0,51662336	7,50821306	2,78740741	0,00757155	0,14265361
TC0500012372.hg.1	LARS1	51520	0,53223266	6,28587806	2,78736846	0,00757233	0,14265361
TC0X00008945.hg.1	PRKX	5613	0,55248142	7,95297474	2,78688418	0,00758206	0,14265361
TC0700010392.hg.1	FERD3L	222894	-0,33536937	6,39052089	-2,78660167	0,00758774	0,14265361
TC0100007505.hg.1	WDTC1	23038	0,41253011	8,87618729	2,78650123	0,00758977	0,14265361
TC0X00011198.hg.1	DNASE1L1	1774	-0,36779551	7,33171374	-2,78549599	0,00761002	0,14288893
TC1000010758.hg.1	RTKN2	219790	-0,20762741	3,95454016	-2,78473469	0,00762539	0,14303221
TC1700012309.hg.1	METTL23	124512	0,67641238	8,38557097	2,78386629	0,00764296	0,14312802
TC1000010916.hg.1	NPFFR1	64106	-0,34482966	5,22001994	-2,78371665	0,00764599	0,14312802
TC1900011620.hg.1	ZNF837	116412	-0,27731904	6,65382051	-2,78327377	0,00765497	0,14314043
TC0100013428.hg.1	MAP3K6	9064	-0,27953825	5,67807255	-2,78291996	0,00766215	0,14314043
TC1700008378.hg.1	MSI2	124540	0,54182467	6,83376472	2,78176193	0,00768569	0,14343521
TC1100006726.hg.1	SMPD1	6609	0,54045417	5,97493381	2,78029115	0,00771569	0,14366683
TC2200007186.hg.1	ISX	91464	-0,30339316	6,18514175	-2,78016165	0,00771834	0,14366683
TC1600009076.hg.1	RPL3L	6123	-0,40386345	7,63327497	-2,78001021	0,00772143	0,14366683
TC1900009254.hg.1	SGTA	6449	0,36410618	7,94454803	2,77848367	0,0077527	0,14410353
TC0200015330.hg.1	C2orf66	401027	-0,26119224	5,1020112	-2,77543365	0,00781553	0,14512534
TC0X00009842.hg.1	SPIN2A	54466	0,36638303	7,62406107	2,77412352	0,00784266	0,14548293
TC1700011262.hg.1	SEPTIN4	5414	-0,37295834	5,2094521	-2,77305207	0,00786491	0,14563531
TC0500007881.hg.1	JMY	133746	0,58685271	6,90923592	2,77230018	0,00788057	0,14563531
TC1000008423.hg.1	RPP30	10556	0,64773108	8,46828151	2,77226365	0,00788133	0,14563531
TC0300009353.hg.1	PPM1L	151742	0,59103593	4,87042931	2,77188166	0,00788929	0,14563531
TC0600009783.hg.1	UST	10090	0,67835983	5,6384173	2,7710261	0,00790715	0,14563531
TC2000007005.hg.1	CST7	8530	0,94338326	8,00130607	2,77101559	0,00790737	0,14563531
TC0100010887.hg.1	C1orf21	81563	1,49730753	6,66169508	2,77084771	0,00791088	0,14563531
TC2000008200.hg.1	PCED1A	64773	0,38462667	8,98922459	2,77070169	0,00791394	0,14563531
TC0300014094.hg.1	DYNLT2B	255758	0,68388971	7,47467893	2,76935988	0,00794205	0,1459122

TC0600012061.hg.1	CILK1	22858	0,55532517	7,09046558	2,76922985	0,00794478	0,1459122
TC1000011081.hg.1	ZNF503	84858	-0,29505628	7,37887953	-2,76849188	0,00796028	0,14605178
TC1100009433.hg.1	HYLS1	219844	0,4932614	4,83316533	2,76415099	0,00805205	0,14737603
TC1000010926.hg.1	TBATA	219793	-0,28288161	5,58704492	-2,76366929	0,0080623	0,14737603
TC1100007826.hg.1	BEST1	7439	-0,45465776	6,17280006	-2,76362035	0,00806334	0,14737603
TC1300008696.hg.1	MRPS31	10240	0,60149961	6,98095937	2,76225913	0,00809235	0,14737603
TC0600010991.hg.1	MBOAT1	154141	-0,81399428	7,73759534	-2,76223171	0,00809294	0,14737603
TC0800010558.hg.1	TOX	9760	0,70844741	8,02458794	2,76219854	0,00809365	0,14737603
TC0300011047.hg.1	IMPDH2	3615	0,35334187	9,27912212	2,76212652	0,00809518	0,14737603
TC1200012644.hg.1	PA2G4	5036	0,5691612	9,38060939	2,76207573	0,00809627	0,14737603
TC1100007776.hg.1	MS4A8	83661	-0,1713381	6,0290343	-2,76123994	0,00811414	0,14755596
TC1600008329.hg.1	HP	3240	-0,26040625	5,23148206	-2,76026848	0,00813496	0,14778908
TC2200006907.hg.1	SGSM1	129049	0,41129168	4,58997161	2,75980005	0,00814501	0,14782641
TC1100007837.hg.1	SCGB1D1	10648	-0,17765642	4,90595445	-2,7590715	0,00816068	0,14796533
TC1600011526.hg.1	PRSS53	339105	-0,30470568	6,55087481	-2,75643737	0,00821754	0,14885025
TC0300007367.hg.1	KLHDC8B	200942	-0,24222354	5,64233946	-2,75588619	0,00822948	0,14892061
TC2000008198.hg.1	CPXM1	56265	-0,23796557	5,68842896	-2,75481324	0,00825278	0,14892859
TC1200008126.hg.1	CPSF6	11052	0,39050118	5,97432307	2,75462891	0,00825679	0,14892859
TC0X00007034.hg.1	NYX	60506	-0,3100314	6,72527561	-2,75462364	0,0082569	0,14892859
TC1600010421.hg.1	MT1G	4495	-0,28763315	8,32300998	-2,75341519	0,00828323	0,14892859
TC0100014514.hg.1	SERBP1	26135	0,2278075	10,6072559	2,75337003	0,00828421	0,14892859
TC2200009033.hg.1	TBC1D22A-AS1	642757	-0,28435441	4,90218834	-2,75307988	0,00829055	0,14892859
TC0100012452.hg.1	TNFRSF18	8784	0,26527073	7,34965635	2,75303804	0,00829146	0,14892859
TC0900011236.hg.1	INIP	58493	0,5944913	7,33505068	2,75225929	0,00830848	0,14892859
TC1200008370.hg.1	RLIG1	91298	0,46076697	7,35574265	2,75225217	0,00830864	0,14892859
TC1700009939.hg.1	PEMT	10400	-0,20787161	6,98175883	-2,75155131	0,00832398	0,14892859
TC1700011131.hg.1	MBTD1	54799	0,53161593	8,11612837	2,75105092	0,00833496	0,14892859
TC1900009442.hg.1	TNFSF14	8740	0,34864701	6,61321919	2,75084586	0,00833946	0,14892859
TC2200009246.hg.1	ZNRF3	84133	-0,2974785	5,8151272	-2,75071442	0,00834234	0,14892859
TC0600011805.hg.1	USP49	25862	-0,31971711	6,09475187	-2,7506947	0,00834277	0,14892859
TC0700010728.hg.1	HERPUD2	64224	0,93340899	8,89422264	2,74879514	0,00838459	0,1495305
TC0800010410.hg.1	PXDNL	137902	-0,24447031	7,32732533	-2,74831986	0,00839508	0,14957323
TC1200012855.hg.1	VSIG10	54621	-0,31858583	5,28484984	-2,74771627	0,00840842	0,1496666
TC1300007884.hg.1	TPP2	7174	0,55872397	7,29611237	2,74707427	0,00842263	0,14977527
TC1200010640.hg.1	NCKAP5L	57701	-0,3005528	6,14271122	-2,74606444	0,00844503	0,14991248
TC2000006535.hg.1	OXT	5020	-0,30325934	7,55179309	-2,74599489	0,00844657	0,14991248

TC0500008157.hg.1	LNPEP	4012	0,55423754	10,289086	2,74560065	0,00845534	0,14992396
TC0600008099.hg.1	POLH	5429	0,64484247	7,54094825	2,74457976	0,00847806	0,15018279
TC0300013877.hg.1	EPHB1	2047	-0,52829728	5,23029117	-2,74334638	0,00850559	0,15048207
TC2200008203.hg.1	DDT	1652	-0,26709241	7,03982892	-2,74253911	0,00852366	0,15048207
TC2000009209.hg.1	KCNS1	3787	-0,26557538	5,07000362	-2,74241959	0,00852633	0,15048207
TC0X00010399.hg.1	BEX1	55859	-0,23255377	4,84396591	-2,74236599	0,00852753	0,15048207
TC1600007952.hg.1	OGFOD1	55239	0,59403626	8,011477	2,73755285	0,00863602	0,15199202
TC0X00009680.hg.1	DGKK	139189	0,26417923	4,04429271	2,73714152	0,00864535	0,15199202
TC1900009854.hg.1	NOTCH3	4854	-0,39142986	6,77585987	-2,73696795	0,00864929	0,15199202
TC1000010006.hg.1	DNAJC1	64215	0,45029806	6,98652678	2,73693247	0,0086501	0,15199202
TC1800006655.hg.1	TXNDC2	84203	-0,32907532	6,60582303	-2,73675024	0,00865423	0,15199202
TC1900007344.hg.1	B3GNT3	10331	-0,34066417	7,50710128	-2,73516188	0,00869038	0,15230033
TC1200007881.hg.1	INHBE	83729	-0,29460826	5,57789343	-2,73512319	0,00869126	0,15230033
TC0400008345.hg.1	SGMS2	166929	-0,84817852	4,25398468	-2,73475929	0,00869957	0,15230033
TC0400009519.hg.1	SNX25	83891	0,78270216	7,41942436	2,73453179	0,00870476	0,15230033
TC0100008752.hg.1	TYW3	127253	0,50470549	6,66093743	2,73403754	0,00871605	0,15235366
TC1900006574.hg.1	IZUMO4	113177	-0,40327459	6,09879414	-2,73286079	0,008743	0,15261891
TC0900009673.hg.1	MLLT3	4300	0,60056431	9,13265446	2,73265363	0,00874775	0,15261891
TC1000011736.hg.1	STN1	79991	0,54964102	7,75947376	2,73150278	0,00877419	0,15287724
TC1500010727.hg.1	CCNDBP1	23582	0,35770148	9,13705798	2,73128907	0,00877911	0,15287724
TC0X00010029.hg.1	RPS4X	6191	0,16423075	12,2842	2,73040663	0,00879944	0,15308703
TC0800006475.hg.1	DLGAP2	9228	-0,2673673	6,55124545	-2,72949103	0,00882058	0,15331049
TC0500009861.hg.1	SLC6A3	6531	-0,21139365	6,47334345	-2,72820041	0,00885046	0,15348642
TC1500010792.hg.1	MCTP2	55784	0,82835005	7,17791288	2,72812234	0,00885227	0,15348642
TC0800012142.hg.1	MROH6	642475	-0,32132156	6,52941676	-2,72753727	0,00886585	0,15348642
TC1300007484.hg.1	PIBF1	10464	0,6081789	6,11265478	2,72729945	0,00887138	0,15348642
TC1200010606.hg.1	WNT10B	7480	-0,28284381	7,03236017	-2,72726236	0,00887224	0,15348642
TC0100009474.hg.1	MAGI3	260425	-0,18878818	4,48101894	-2,72580291	0,00890622	0,15393013
TC1100012229.hg.1	POGLUT3	143888	0,23856256	5,17428576	2,72524309	0,00891929	0,15401188
TC0500013149.hg.1	SRD5A1	6715	-0,287436	5,44764118	-2,72432489	0,00894075	0,15420769
TC1300009993.hg.1	BIVM	54841	0,38348602	4,90177397	2,72398434	0,00894873	0,15420769
TC0500007552.hg.1	LOC100421561	100421561	-0,42310324	8,35336474	-2,72333582	0,00896393	0,15420769
TC1900008134.hg.1	CYP2B6	1555	-0,28460353	4,07682043	-2,72333247	0,00896401	0,15420769
TC1200012813.hg.1	B4GALNT1	2583	-0,37608054	7,24277116	-2,7226594	0,00897982	0,15433588
TC0300010002.hg.1	LINC00885	401109	-0,24925315	5,69623025	-2,72230221	0,00898821	0,15433665
TC0100007540.hg.1	THEMIS2	9473	-0,39923042	7,63241343	-2,7205324	0,00902993	0,15461129

TC1900012052.hg.1	GARIN5B	284418	-0,28058742	5,78459668	-2,72049561	0,0090308	0,15461129
TC0100017072.hg.1	ELK4	2005	0,26691994	11,4420745	2,72039398	0,0090332	0,15461129
TC0100007117.hg.1	ARHGEF10L	55160	-0,6772432	6,33187429	-2,72020441	0,00903768	0,15461129
TC0300008853.hg.1	TMEM108	66000	-0,22254721	4,77406638	-2,71913582	0,00906298	0,15490059
TC1300006619.hg.1	ATP12A	479	-0,20072968	4,52634699	-2,71877742	0,00907147	0,15490254
TC1900007679.hg.1	PLEKHF1	79156	0,46260982	7,34796626	2,71752999	0,00910111	0,15499153
TC1200010559.hg.1	VDR	7421	-0,555331	8,64446643	-2,71716577	0,00910978	0,15499153
TC1500007619.hg.1	SMAD6	4091	-0,30916316	4,24288917	-2,71712134	0,00911084	0,15499153
TC1500010887.hg.1	CLN6	54982	-0,3351642	6,81838482	-2,71696674	0,00911452	0,15499153
TC0300008271.hg.1	BBX	56987	0,48416832	8,08139968	2,71679429	0,00911863	0,15499153
TC0500013414.hg.1	THOC3	84321	0,66432257	7,39741009	2,7147253	0,00916807	0,15568861
TC0200008984.hg.1	RABL2A	11159	0,56781406	6,90349576	2,71417364	0,00918129	0,15571498
TC0100006681.hg.1	RNF207	388591	-0,30743446	6,9029525	-2,7139575	0,00918648	0,15571498
TC1400008764.hg.1	GZMB	3002	0,50523833	8,9132676	2,71350499	0,00919734	0,15575626
TC1800007101.hg.1	KIAA1328	57536	0,43259599	7,91947066	2,71235255	0,00922507	0,15608272
TC2000010010.hg.1	BLCAP	10904	-0,31089486	7,34840924	-2,71171948	0,00924033	0,15619792
TC0200009938.hg.1	GORASP2	26003	0,44148526	7,64482571	2,71078306	0,00926295	0,15639719
TC0500008055.hg.1	ADGRV1	84059	-0,20796741	3,86134215	-2,71037337	0,00927286	0,15639719
TC1700012449.hg.1	CSHL1	1444	-0,2491633	6,07392193	-2,70995351	0,00928303	0,15639719
TC0700010182.hg.1	PMS2	5395	0,52861496	8,67304317	2,70975707	0,00928779	0,15639719
TC2000007142.hg.1	BPIFB4	149954	-0,2313956	4,11032214	-2,70948275	0,00929444	0,15639719
TC1000010925.hg.1	PRF1	5551	1,1533532	9,01393395	2,70731545	0,00934715	0,15700981
TC0700006520.hg.1	MAFK	7975	-0,40375131	6,43132031	-2,70682584	0,0093591	0,15700981
TC1200010591.hg.1	CCNT1	904	0,47435105	8,09436551	2,70661759	0,00936418	0,15700981
TC2000009966.hg.1	FKBP1A-SDCBP2	100528031	-0,20891746	7,51368696	-2,70659066	0,00936484	0,15700981
TC0300008962.hg.1	FOXL2NB	401089	-0,29281604	6,028618	-2,7061324	0,00937604	0,15705507
TC1300008417.hg.1	GPR12	2835	-0,21304287	3,83232124	-2,70426296	0,00942186	0,15751606
TC0200010255.hg.1	PMS1	5378	0,42319482	4,959773	2,70407071	0,00942658	0,15751606
TC0800008194.hg.1	NECAB1	64168	-0,18169256	4,15970263	-2,70368928	0,00943596	0,15751606
TC0700011969.hg.1	MCM7	4176	0,47704787	6,05607111	2,70362	0,00943766	0,15751606
TC0500010628.hg.1	ANXA2R	389289	0,66899995	8,9656823	2,70273688	0,00945941	0,1576779
TC0600007555.hg.1	MUCL3	135656	-0,23171446	6,3630401	-2,70253348	0,00946443	0,1576779
TC0X00009498.hg.1	FUNDC1	139341	0,28567165	5,46798932	2,70187406	0,00948071	0,15780679
TC1400007860.hg.1	FLRT2	23768	-0,1901704	4,61601145	-2,70123484	0,00949651	0,15790509
TC1600010439.hg.1	PSME3IP1	80011	0,72554686	7,18097384	2,70055367	0,00951338	0,15790509
TC0300008703.hg.1	PODXL2	50512	-0,21170088	7,99281495	-2,70007177	0,00952533	0,15790509

TC0300013296.hg.1	DNAJC19	131118	0,58624495	8,72036088	2,69982487	0,00953146	0,15790509
TC1400008677.hg.1	SLC7A7	9056	-0,75973457	6,55833595	-2,69888078	0,00955492	0,15790509
TC1600009198.hg.1	NLRC3	197358	0,55917109	8,02534082	2,69869568	0,00955953	0,15790509
TC1200010850.hg.1	TESPA1	9840	0,72840372	7,73030952	2,69794853	0,00957814	0,15790509
TC1100008071.hg.1	TMEM151A	256472	-0,25168558	6,32645924	-2,69789156	0,00957956	0,15790509
TC0100009658.hg.1	SRGAP2C	653464	-0,37625378	8,82017861	-2,69787868	0,00957988	0,15790509
TC0400006731.hg.1	LOC389199	389199	-0,26392355	5,84650703	-2,6976122	0,00958653	0,15790509
TC1100008385.hg.1	RELT	84957	-0,45306404	8,52033564	-2,69751266	0,00958902	0,15790509
TC1700010571.hg.1	MED1	5469	0,46347194	8,39827836	2,69750654	0,00958917	0,15790509
TC0600008780.hg.1	CASP8AP2	9994	0,85147068	7,11836812	2,69658892	0,0096121	0,15814178
TC0100015401.hg.1	IGSF3	3321	-0,23677494	4,64209568	-2,6959355	0,00962846	0,15827002
TC0300013784.hg.1	CAV3	859	-0,29151444	5,96739496	-2,69525313	0,00964558	0,15841038
TC0100018045.hg.1	SMYD3	64754	0,55015817	7,11137787	2,69482722	0,00965627	0,15844519
TC1900010466.hg.1	LGI4	163175	-0,29038292	5,8983685	-2,69354417	0,00968856	0,1588339
TC0200008801.hg.1	SULT1C4	27233	-0,20647511	4,53654818	-2,6929356	0,00970391	0,15894449
TC1200012584.hg.1	TAPBPL	55080	0,35231055	9,08105097	2,69218746	0,00972281	0,15911298
TC1700012354.hg.1	VAMP2	6844	0,3626408	8,47259769	2,69091039	0,00975514	0,15933643
TC1200009649.hg.1	CRACR2A	84766	0,50978826	7,09141665	2,69060865	0,0097628	0,15933643
TC1200010653.hg.1	CERS5	91012	0,68874753	8,25008928	2,69039451	0,00976824	0,15933643
TC0600010210.hg.1	UNC93A	54346	-0,23007165	5,54151808	-2,69018407	0,00977358	0,15933643
TC0800008629.hg.1	RAD21-AS1	644660	-0,22403463	5,34463701	-2,68994799	0,00977958	0,15933643
TC1600007990.hg.1	RSPRY1	89970	0,77705895	7,86454379	2,68943449	0,00979264	0,15936689
TC1500008317.hg.1	CRTC3	64784	0,41684273	7,82802951	2,68919646	0,0097987	0,15936689
TC0100008336.hg.1	SCP2	6342	0,52011423	8,63933304	2,68853125	0,00981565	0,15938992
TC1900006647.hg.1	CACTIN-AS1	404665	-0,4480074	6,4247085	-2,68838637	0,00981935	0,15938992
TC1100011811.hg.1	CCDC90B	60492	0,79666951	7,1782811	2,68811862	0,00982618	0,15938992
TC1900009417.hg.1	MLLT1	4298	-0,30229535	7,54119193	-2,68778815	0,00983462	0,15938992
TC0700010162.hg.1	FBXL18	80028	-0,30244818	6,65502087	-2,68742202	0,00984398	0,15940177
TC1200009892.hg.1	OLR1	4973	-1,03208014	5,40692651	-2,68493372	0,00990781	0,15988254
TC1200010264.hg.1	IPO8	10526	0,61979859	6,82721573	2,68460453	0,00991628	0,15988254
TC2000008279.hg.1	PCNA	5111	0,62355571	8,6321383	2,68438444	0,00992195	0,15988254
TC0100009344.hg.1	SARS1	6301	0,29745931	8,05760455	2,68427315	0,00992481	0,15988254
TC1700007016.hg.1	TRPV2	51393	0,27855292	10,0628841	2,68392562	0,00993377	0,15988254
TC0700011167.hg.1	NUPR2	389493	-0,2013018	6,77675164	-2,68384154	0,00993594	0,15988254
TC0400007799.hg.1	SLC4A4	8671	0,54857558	5,10072282	2,68379929	0,00993703	0,15988254
TC0100007463.hg.1	HMGN2	3151	0,34396853	9,07857834	2,68329567	0,00995003	0,15988254

TC0900012187.hg.1	KCNT1	57582	-0,37662025	6,01507361	-2,68285946	0,0099613	0,15988254
TC0200011686.hg.1	ITGB1BP1	9270	0,59235284	5,75656426	2,68259403	0,00996816	0,15988254
TC0400007128.hg.1	TBC1D19	55296	0,61542369	6,6585414	2,68256704	0,00996886	0,15988254
TC1000010509.hg.1	AGAP4	119016	0,27126464	9,70500696	2,68200291	0,00998347	0,1599779

Supplementary Table 6. List of differentially expressed genes in T cells between post-treatment and pre-treatment with belimumab.

Affymetrix ID	Gene Symbol	Entrez	logFC	AveExpr	t	P.Value	adj.P.Val	B
TC0500008785.hg.1	EGR1	1958	1,40195791	7,43336618	4,9073897	1,09E-05	0,20109879	-1,03496989
TC0800009856.hg.1	EGR3	1960	1,00216141	6,69851648	4,09476393	0,00015996	0,99996468	-1,95598813
TC1200012573.hg.1	RHNO1	83695	-0,97083546	5,66051933	-4,07756713	0,000169	0,99996468	-1,97534086
TC0100014769.hg.1	MCOLN3	55283	0,88511019	4,16876028	3,67479905	0,00059557	0,99996468	-2,42371146
TC0100018263.hg.1	GSTM1	2944	0,4239253	5,77406366	3,61597483	0,00071238	0,99996468	-2,48818398
TC0100009364.hg.1	CSF1	1435	0,7705598	5,4437603	3,59540008	0,0007582	0,99996468	-2,51065919
TC0500009599.hg.1	TSPAN17	26262	-0,48360054	6,93692801	-3,53817783	0,00090092	0,99996468	-2,57295078
TC0100012469.hg.1	MXRA8	54587	-0,43620159	4,86061143	-3,52476592	0,0009379	0,99996468	-2,58750322
TC0800010360.hg.1	PRKDC	5591	-0,4266025	7,26919715	-3,52158406	0,00094689	0,99996468	-2,59095293
TC0700008747.hg.1	HBP1	26959	-0,4983978	9,32369731	-3,43830449	0,00121349	0,99996468	-2,68085693
TC1200008113.hg.1	NUP107	57122	-0,35215583	6,20104689	-3,35178069	0,00156543	0,99996468	-2,77342601
TC0600011463.hg.1	NEU1	4758	0,89240242	8,76441406	3,32453288	0,00169502	0,99996468	-2,80238891
TC1700011210.hg.1	COIL	8161	-0,44864408	5,14717815	-3,25691905	0,00206189	0,99996468	-2,87384508
TC0600012123.hg.1	DST	667	0,88628908	5,07307204	3,20339637	0,00240432	0,99996468	-2,92997187
TC2000009890.hg.1	ANKEF1	63926	0,35906522	4,54698726	3,13480775	0,00292187	0,99996468	-3,00130013
TC0500011255.hg.1	MTX3	345778	-0,60506619	4,94114636	-3,12178464	0,0030313	0,99996468	-3,01476464
TC0200006891.hg.1	RHOB	388	1,08115065	8,89262857	3,12011612	0,00304559	0,99996468	-3,01648786
TC1000007889.hg.1	SUPV3L1	6832	-0,46909923	4,88476361	-3,06953389	0,00351028	0,99996468	-3,06852416
TC0300010933.hg.1	FYCO1	79443	-0,48483752	7,3232712	-3,06490853	0,00355594	0,99996468	-3,07326247
TC0300014049.hg.1	ACAD11	84129	-0,38049106	4,75449635	-3,0604353	0,00360063	0,99996468	-3,07784169
TC0200015349.hg.1	HSPD1	3329	1,0431638	7,89926611	3,05221481	0,00368413	0,99996468	-3,08624863
TC0200012318.hg.1	CDKL4	344387	0,21426924	4,16516392	3,04575449	0,00375102	0,99996468	-3,09284783
TC1900009604.hg.1	COL5A3	50509	-0,2248374	6,52819898	-3,03247183	0,00389213	0,99996468	-3,10639484
TC1500010463.hg.1	PRC1	9055	-0,93008585	5,72398165	-3,02309529	0,00399473	0,99996468	-3,11594064
TC1700006480.hg.1	TIMM22	29928	0,31745515	6,57260699	3,01935208	0,00403639	0,99996468	-3,11974739
TC1200007653.hg.1	NR4A1	3164	0,69860617	8,42809903	2,98959702	0,0043823	0,99996468	-3,14992477
TC0600011315.hg.1	ZFP57	346171	-0,55697952	4,63720629	-2,98396122	0,00445086	0,99996468	-3,15562383
TC1000007895.hg.1	TSPAN15	23555	0,39394392	4,2928451	2,97785906	0,00452623	0,99996468	-3,16178842
TC1100009969.hg.1	APBB1	322	-0,55479072	6,37219647	-2,93852157	0,0050414	0,99996468	-3,20137491
TC0X00008401.hg.1	RAB33A	9363	0,38807214	6,16987961	2,93300755	0,00511783	0,99996468	-3,20690235
TC1900008496.hg.1	PPP1R15A	23645	0,66077614	10,2642896	2,92804454	0,00518755	0,99996468	-3,21187285
TC0900011454.hg.1	ZBTB26	57684	-0,61689807	5,17691587	-2,92032655	0,00529773	0,99996468	-3,21959386
TC0200013323.hg.1	CD8A	925	-0,71403398	7,21326631	-2,91722539	0,00534261	0,99996468	-3,22269326

TC1100009744.hg.1	DUSP8	1850	-0,29815779	5,7251692	-2,91556392	0,0053668	0,99996468	-3,22435308
TC0200014609.hg.1	RBM43	375287	-0,37446563	5,95057025	-2,90862136	0,00546899	0,99996468	-3,23128342
TC0300008530.hg.1	ARGFX	503582	-0,2368303	4,93753072	-2,899313	0,00560885	0,99996468	-3,24056185
TC2000008741.hg.1	ABHD12	26090	-0,25853794	6,245859	-2,88342091	0,00585534	0,99996468	-3,25636668
TC0100018323.hg.1	IER5	51278	0,67814994	6,3772309	2,87860232	0,00593204	0,99996468	-3,26114974
TC1200010618.hg.1	TUBA1A	7846	0,58998178	9,27712964	2,84171793	0,00655083	0,99996468	-3,29762044
TC0X00010515.hg.1	CAPN6	827	-0,19935311	3,95155691	-2,81755442	0,00698811	0,99996468	-3,32137495
TC0700006632.hg.1	OCM	654231	-0,27693922	5,51318852	-2,80835017	0,00716167	0,99996468	-3,3303942
TC2200009364.hg.1	CPT1B	1375	-0,3469104	7,55970717	-2,80834938	0,00716169	0,99996468	-3,33039498
TC0600011459.hg.1	HSPA1L	3305	0,24685562	5,97577489	2,79486226	0,00742323	0,99996468	-3,34358162
TC0300006513.hg.1	GRM7	2917	-0,26142618	5,41891819	-2,7833483	0,00765346	0,99996468	-3,3548112
TC0800007866.hg.1	MCMDC2	157777	-0,18921686	3,89298577	-2,77561134	0,00781186	0,99996468	-3,36234255
TC0800011116.hg.1	RBM12B	389677	-0,33004273	4,92669807	-2,76487594	0,00803666	0,99996468	-3,37277318
TC2200008827.hg.1	TOB2	10766	0,29813854	7,93493646	2,73829554	0,0086192	0,99996468	-3,39850069
TC0600007869.hg.1	CMTR1	23070	-0,35245911	6,99003283	-2,72785877	0,00885839	0,99996468	-3,40856388
TC2200006833.hg.1	BCR	613	-0,45601324	6,11055783	-2,71438928	0,00917612	0,99996468	-3,42151864
TC1100007819.hg.1	MYRF	745	-0,24173413	6,86631937	-2,71217911	0,00922925	0,99996468	-3,42364081
TC1500009621.hg.1	GTF2A2	2958	0,38350668	7,32290771	2,70068906	0,00951002	0,99996468	-3,43465731
TC1300006690.hg.1	POLR1D	51082	0,36631204	6,59849297	2,69934415	0,0095434	0,99996468	-3,43594502
TC0X00010374.hg.1	NXF5	55998	-0,19412762	4,37199957	-2,69003479	0,00977737	0,99996468	-3,44484819
TC0X00010278.hg.1	NAP1L3	4675	0,21732283	4,34890282	2,68821514	0,00982372	0,99996468	-3,44658635

Supplementary Table 7. List of differentially miRNAs in T cells between post-treatment and pre-treatment with belimumab.

Probe.Set.Name	Accession	Symbol	Sequence.Type	logFC	AveExpr	t	P.Value	adj.P.Val	B
MIMAT0019846_st	MIMAT0019846	hsa-miR-4726-3p	miRNA	0,20511005	1,51269287	3,55179593	0,00088185	0,80352678	-1,77518521
MIMAT0019831_st	MIMAT0019831	hsa-miR-4718	miRNA	-0,23515833	1,56004083	-3,53836716	0,00091796	0,80352678	-1,79614862
MIMAT0026480_st	MIMAT0026480	hsa-miR-153-5p	miRNA	-0,22886215	1,53405256	-3,53218194	0,00093506	0,80352678	-1,80579387
MIMAT0019213_st	MIMAT0019213	hsa-miR-3162-3p	miRNA	-0,21572044	1,65021017	-3,12729487	0,00302362	0,98035236	-2,42106831
MIMAT0026741_st	MIMAT0026741	hsa-miR-1251-3p	miRNA	-0,19385884	1,49252341	-3,07626785	0,00348756	0,98035236	-2,49606903
MIMAT0019034_st	MIMAT0019034	hsa-miR-4419b	miRNA	-0,26908504	1,61256501	-3,07611653	0,00348903	0,98035236	-2,49629049
MIMAT0004923_st	MIMAT0004923	hsa-miR-875-3p	miRNA	-0,25159873	1,6058463	-3,03620039	0,00389787	0,98035236	-2,5545128
MIMAT0004601_st	MIMAT0004601	hsa-miR-145-3p	miRNA	-0,22848951	1,52930604	-2,976104	0,00459885	0,98035236	-2,64140667
MIMAT0003300_st	MIMAT0003300	hsa-miR-631	miRNA	0,21546136	1,60013346	2,96114653	0,00479078	0,98035236	-2,66288755
MIMAT0027476_st	MIMAT0027476	hsa-miR-6788-5p	miRNA	0,18846594	1,58805336	2,88779281	0,00584507	0,98035236	-2,76736204
MIMAT0000448_st	MIMAT0000448	hsa-miR-136-5p	miRNA	0,19688595	1,50951602	2,88571917	0,0058778	0,98035236	-2,77029402
MIMAT0022928_st	MIMAT0022928	hsa-miR-376a-2-5p	miRNA	0,19707653	1,5211866	2,8750928	0,0060482	0,98035236	-2,78530005
MIMAT0002813_st	MIMAT0002813	hsa-miR-493-5p	miRNA	0,20453286	1,57589027	2,84705863	0,00652004	0,98035236	-2,82473616
MIMAT0015043_st	MIMAT0015043	hsa-miR-3168	miRNA	-0,19260531	1,53884095	-2,81397387	0,00712077	0,98035236	-2,87098857
MIMAT0025474_st	MIMAT0025474	hsa-miR-6509-5p	miRNA	-0,26422489	1,70955989	-2,81258743	0,00714703	0,98035236	-2,87291992
MIMAT0005906_st	MIMAT0005906	hsa-miR-1255a	miRNA	-0,20274532	1,58993966	-2,81162332	0,00716534	0,98035236	-2,8742626
MIMAT0004795_st	MIMAT0004795	hsa-miR-574-5p	miRNA	-0,23752824	1,6589725	-2,79291854	0,00752935	0,98035236	-2,90025862

MIMAT0019933_st	MIMAT0019933	hsa-miR-4776-3p	miRNA	0,2106271	1,57433559	2,7750182	0,00789366	0,98035236	-2,92504024
MIMAT0005904_st	MIMAT0005904	hsa-miR-1253	miRNA	-0,28763819	1,64069105	-2,76428164	0,00811992	0,98035236	-2,93985853
MIMAT0019938_st	MIMAT0019938	hsa-miR-4779	miRNA	-0,23660451	1,64723831	-2,76309099	0,00814538	0,98035236	-2,94149971
MIMAT0004499_st	MIMAT0004499	hsa-miR-26a-1-3p	miRNA	-0,21676393	1,5613243	-2,74142483	0,00862168	0,98035236	-2,97128974
MIMAT0000728_st	MIMAT0000728	hsa-miR-375	miRNA	-0,16441817	1,56548816	-2,73685404	0,00872539	0,98035236	-2,97755627
MIMAT0002827_st	MIMAT0002827	hsa-miR-515-3p	miRNA	-0,15694718	1,54147944	-2,70279307	0,00953519	0,98035236	-3,02405267
MIMAT0002881_st	MIMAT0002881	hsa-miR-509-3p	miRNA	0,28178606	1,5775027	2,68471278	0,00999263	0,98035236	-3,04858844

Supplementary Table 8. List of differentially miRNAs in B cells between post-treatment and pre-treatment with belimumab.

Probe.Set.Name	Accession	Symbol	Sequence.Type	logFC	AveExpr	t	P.Value	adj.P.Val	B
MIMAT0019809_st	MIMAT0019809	hsa-miR-4708-5p	miRNA	-0,3158212	1,64740258	-3,64067799	0,00067499	0,99434628	-1,63568023
MIMAT0021025_st	MIMAT0021025	hsa-miR-5003-5p	miRNA	-0,28264254	1,6430962	-3,50708931	0,00100761	0,99434628	-1,84485533
MIMAT0019743_st	MIMAT0019743	hsa-miR-4667-5p	miRNA	-0,26610943	1,68288136	-3,35931623	0,00155656	0,99434628	-2,07255688
MIMAT0017392_st	MIMAT0017392	hsa-miR-3200-5p	miRNA	-0,26511495	1,68494597	-3,30502958	0,00182203	0,99434628	-2,15513772
MIMAT0026476_st	MIMAT0026476	hsa-miR-215-3p	miRNA	0,27101859	1,55025746	3,20241224	0,00244518	0,99434628	-2,30953882
MIMAT0030980_st	MIMAT0030980	hsa-miR-8053	miRNA	0,21828701	1,55767161	3,17484226	0,00264415	0,99434628	-2,35062436
MIMAT0027453_st	MIMAT0027453	hsa-miR-6776-3p	miRNA	0,20984558	1,54897422	3,10130383	0,00325215	0,99434628	-2,45934929
MIMAT0004681_st	MIMAT0004681	hsa-miR-26a-2-3p	miRNA	-0,22225427	1,57645184	-3,0752522	0,00349744	0,99434628	-2,49755541
MIMAT0019720_st	MIMAT0019720	hsa-miR-4654	miRNA	-0,29863135	1,60569756	-3,04543194	0,0037995	0,99434628	-2,54108295
MIMAT0023704_st	MIMAT0023704	hsa-miR-6079	miRNA	0,29639933	1,7062022	2,94563943	0,00499764	0,99434628	-2,68509503
MIMAT0012734_st	MIMAT0012734	hsa-miR-711	miRNA	-0,20558592	1,63466086	-2,87988087	0,00597086	0,99434628	-2,77854248
MIMAT0000102_st	MIMAT0000102	hsa-miR-105-5p	miRNA	-0,2200193	1,55188239	-2,86421948	0,00622731	0,99434628	-2,80062207
MIMAT0005937_st	MIMAT0005937	hsa-miR-1279	miRNA	-0,23592769	1,59760931	-2,7749408	0,00789527	0,99434628	-2,92514719
MIMAT0016867_st	MIMAT0016867	hsa-miR-4316	miRNA	-0,22351614	1,53718138	-2,74907221	0,0084507	0,99434628	-2,96079109
MIMAT0019937_st	MIMAT0019937	hsa-miR-4778-3p	miRNA	-0,15464916	1,50409103	-2,74798323	0,00847486	0,99434628	-2,96228716
MIMAT0019928_st	MIMAT0019928	hsa-miR-4773	miRNA	0,2263886	1,63515638	2,74266324	0,00859378	0,99434628	-2,9695908
MIMAT0016883_st	MIMAT0016883	hsa-miR-4251	miRNA	0,24637243	1,65279208	2,72500721	0,00899957	0,99434628	-2,99376862
MIMAT0022266_st	MIMAT0022266	hsa-miR-548ar-3p	miRNA	0,17617891	1,50930653	2,71457915	0,00924746	0,99434628	-3,00800388
MIMAT0011157_st	MIMAT0011157	hsa-miR-2114-3p	miRNA	0,18116755	1,54380513	2,69192302	0,00980784	0,99434628	-3,03881602

Supplementary Table 9. List of differentially miRNAs in myeloid cells between post-treatment and pre-treatment with belimumab.

Probe.Set.Name	Accession	Symbol	Sequence.Type	logFC	AveExpr	t	P.Value	adj.P.Val	B
MIMAT0018352_st	MIMAT0018352	hsa-miR-3937	miRNA	0,72934292	1,97385119	3,98075268	0,00023643	0,50392876	-0,59617932
MIMAT0018959_st	MIMAT0018959	hsa-miR-4441	miRNA	0,38884875	1,70382617	3,64517645	0,00066587	0,50392876	-1,22385093
MIMAT0007883_st	MIMAT0007883	hsa-miR-1909-3p	miRNA	0,8516397	2,08741401	3,60909771	0,0007425	0,50392876	-1,29009718
MIMAT0028121_st	MIMAT0028121	hsa-miR-7112-5p	miRNA	0,34168503	1,68163543	3,56878198	0,00083813	0,50392876	-1,36380379
MIMAT0027460_st	MIMAT0027460	hsa-miR-6780a-5p	miRNA	0,40256792	1,7911657	3,45755451	0,00116691	0,50392876	-1,56531509
MIMAT0031119_st	MIMAT0031119	hsa-miR-1199-5p	miRNA	0,3158925	1,59326969	3,45583746	0,00117284	0,50392876	-1,56840387
MIMAT0018939_st	MIMAT0018939	hsa-miR-4424	miRNA	-0,2547842	1,58066145	-3,30640034	0,00181483	0,57410083	-1,8344867
MIMAT0016925_st	MIMAT0016925	hsa-miR-500b-5p	miRNA	-0,30628239	1,75258221	-3,28808827	0,00191331	0,57410083	-1,86670491
MIMAT0019711_st	MIMAT0019711	hsa-miR-4649-5p	miRNA	0,77122937	2,26491786	3,27195936	0,00200423	0,57410083	-1,89500894
MIMAT0015065_st	MIMAT0015065	hsa-miR-3185	miRNA	1,39677369	2,88587096	3,21278108	0,00237408	0,61203697	-1,99825936
MIMAT0015061_st	MIMAT0015061	hsa-miR-3181	miRNA	0,28981212	1,58460703	3,02547442	0,00401515	0,69457025	-2,31848347
MIMAT0015079_st	MIMAT0015079	hsa-miR-3195	miRNA	0,86281827	2,04240394	3,01801011	0,0040987	0,69457025	-2,33102629
MIMAT0019765_st	MIMAT0019765	hsa-miR-4680-3p	miRNA	-0,1981782	1,58360497	-3,00646551	0,00423115	0,69457025	-2,35039115
MIMAT0000270_st	MIMAT0000270	hsa-miR-181a-3p	miRNA	-0,31937097	1,5968113	-2,99633618	0,00435065	0,69457025	-2,3673475
MIMAT0019011_st	MIMAT0019011	hsa-miR-4479	miRNA	0,35621984	1,60366733	2,9926828	0,00439452	0,69457025	-2,37345522
MIMAT0004948_st	MIMAT0004948	hsa-miR-885-3p	miRNA	0,51755011	2,09783477	2,99181488	0,00440501	0,69457025	-2,3749056
MIMAT0016899_st	MIMAT0016899	hsa-miR-4264	miRNA	-0,19968907	1,4935729	-2,91929364	0,0053683	0,69457025	-2,49523773
MIMAT0019911_st	MIMAT0019911	hsa-miR-4762-3p	miRNA	-0,19099467	1,53747736	-2,90553755	0,00557186	0,69457025	-2,51786841
MIMAT0003235_	MIMAT000323	hsa-miR-570-3p	miRNA	0,24314193	1,6037816	2,89336708	0,0057579	0,6945702	-2,5378379

st	5				7		3	5	
MIMAT0019037_st	MIMAT0019037	hsa-miR-4501	miRNA	-0,24503025	1,66401463	-2,87147048	0,00610733	0,69457025	-2,57364064
MIMAT0015066_st	MIMAT0015066	hsa-miR-3065-5p	miRNA	0,19325571	1,61268078	2,85867228	0,00632057	0,69457025	-2,59449139
MIMAT0025855_st	MIMAT0025855	hsa-miR-6723-5p	miRNA	0,37886907	1,74981747	2,85280482	0,00642063	0,69457025	-2,60403189
MIMAT0027634_st	MIMAT0027634	hsa-miR-6867-5p	miRNA	-0,21547653	1,58130411	-2,81617839	0,0070792	0,69457025	-2,66331766
MIMAT0004493_st	MIMAT0004493	hsa-miR-20a-3p	miRNA	-0,21767097	1,56357946	-2,81205695	0,0071571	0,69457025	-2,66995963
MIMAT0005572_st	MIMAT0005572	hsa-miR-1225-5p	miRNA	0,45349341	1,87385914	2,78558434	0,00767669	0,69457025	-2,71247915
MIMAT0022977_st	MIMAT0022977	hsa-miR-4632-5p	miRNA	0,8494832	2,56106097	2,77355511	0,00792415	0,69457025	-2,7317179
MIMAT0018108_st	MIMAT0018108	hsa-miR-3681-5p	miRNA	-0,20868557	1,51252215	-2,77204632	0,0079557	0,69457025	-2,7341273
MIMAT0022263_st	MIMAT0022263	hsa-miR-548aq-5p	miRNA	-0,19603214	1,55060453	-2,77171815	0,00796258	0,69457025	-2,73465124
MIMAT0005586_st	MIMAT0005586	hsa-miR-1231	miRNA	0,59104166	2,01394957	2,76063966	0,00819802	0,69457025	-2,75231627
MIMAT0019208_st	MIMAT0019208	hsa-miR-3074-5p	miRNA	0,14117389	1,52574139	2,74183071	0,00861252	0,69457025	-2,7822062
MIMAT0027498_st	MIMAT0027498	hsa-miR-6799-5p	miRNA	0,81161991	2,43838696	2,72311649	0,00904405	0,69457025	-2,81181767
MIMAT0027469_st	MIMAT0027469	hsa-miR-6784-3p	miRNA	-0,22909601	1,65771857	-2,71337503	0,00927648	0,69457025	-2,82718066
MIMAT0027621_st	MIMAT0027621	hsa-miR-6769b-3p	miRNA	0,19051692	1,53424797	2,70526496	0,00947417	0,69457025	-2,83994409
MIMAT0019964_st	MIMAT0019964	hsa-miR-4792	miRNA	0,22896278	1,57702702	2,7000908	0,00960231	0,69457025	-2,84807431
MIMAT0019764_st	MIMAT0019764	hsa-miR-4680-5p	miRNA	0,21306302	1,62594851	2,69839724	0,0096446	0,69457025	-2,85073326
MIMAT0012734_st	MIMAT0012734	hsa-miR-711	miRNA	0,19247498	1,63466086	2,69622067	0,0096992	0,69457025	-2,85414897

Supplementary Table S10. Baseline demographic and clinical characteristics of non-Belimumab treated SLE cohort

Characteristics	Non-Belimumab treated SLE patients (n=5)	
	**Baseline**	**6 months-treatment**
**Age, mean (SD), years**	36.4 (10.4)	37.6 (10.1)
**Female, n (%)**	80	80
**Duration of SLE disease, mean (SD), years**	12.4 (4)	13.6 (3.6)
**SLE disease activity**		
**Total SLEDAI-2K score, mean (SD)**	11 (3.3)	2.6 (4.7)
**PGA, mean (SD)**	1.3 (1.92)	0.72 (0.71)
**Clinical manifestations, (%)**		
**Musculoskeletal**	3 (60)	0 (0)
**Mucocutaneous**	3 (60)	0 (0)
**Cardiorespiratory**	1 (20)	0 (20)
**Haematological**	3 (60)	1 (20)
**Renal involvement n, %**	0 (0)	0 (0)
**Immunological profile**		
**Anti-dsDNA antibodies positive, n (%)**	5 (100)	2 (40)
**Low complement (C3 and/orC4), n (%)**	3 (60)	2 (40)
**Treatment at baseline, n**		
**Glucocorticoids, n (%)**<br>**Daily prednisone dose, mean (SD), mg/day**	5 (100)<br>12.5 (2.8)	5 (100)<br>3.5 (2.7)
**Antimalarial agents, n (%)**	5 (100)	5 (100)
**Immunosuppressants, n (%)**	5 (100)	5 (100)
**Mycophenolate mofetil**	5 (100)	5 (100)

Abbreviations: SLE= Systemic lupus erythematosus; SLEDAI-2K= Systemic Lupus Erythematosus Disease Activity Index 2000; PGA= physician's global assessment of disease activity. Reference ranges are as follows: anti-double-stranded DNA antibodies, <15 IU per milliliter; serum C3 (mg/dL), 85 to 110; serum C4 (mg/dL), 10 to 40.
